# Activity-dependent regulation of microglia numbers by pyramidal cells during development shape cortical functions

**DOI:** 10.1126/sciadv.adq5842

**Published:** 2025-02-19

**Authors:** Sanjana Kumaraguru, James Morgan, Fong Kuan Wong

**Affiliations:** ^1^ Division of Developmental Biology and Medicine, Faculty of Biology, Medicine and Health, University of Manchester, Manchester M13 9PT, UK.; ^2^ Lydia Becker Institute of Immunology and Inflammation, Faculty of Biology, Medicine and Health, University of Manchester, UK.

## Abstract

Beyond their role as immune sentinels, microglia are actively involved in establishing and maintaining cortical circuits. Alteration in microglial numbers has been associated with abnormal behaviors akin to those observed in neurodevelopmental disorders. Consequently, establishing the appropriate microglial numbers during development is crucial for ensuring normal cortical function. Here, we uncovered a dynamic relationship between pyramidal cells and microglia that tunes microglial numbers and development through distinct phases of mouse postnatal development. Changes in pyramidal cell activity during development induce differential release of activity-dependent proteins such as Activin A, which, in turn, adjusts microglial numbers accordingly. Decoupling of this relationship not only changes microglial numbers but has a long-term consequence on their role as synaptic organizers, which ultimately affects cortical function. Our findings reveal that microglia adapt their numbers to changes in pyramidal cell activity during a critical time window in development, consequently adjusting their numbers and function to the demands of the developing local circuits.

## Teaser

Decoupling of microglia and pyramidal cell interaction disrupts microglial number and function and induce cognitive deficits.

## INTRODUCTION

Microglia are resident immune cells in the parenchyma of the central nervous system (CNS) (*1*). Recent studies have revealed the diverging roles microglia play during normal brain development and later in adulthood beyond their remit as immune sentinels and effectors (*2*, *3*). Their early presence in the CNS has allowed them to have a coarse impact on the cytoarchitecture of the brain through the modulation of neuronal numbers and positioning (*4*–*7*). Later, microglia are known to induce more subtle changes within the brain as they are known to influence cortical circuit construction and maintenance through their roles as synaptic organizers (*8*).

The various roles of microglia during normal brain development arise from their ability to sense and react to changes in their local environment. Microglia express genes that encode proteins that detect environmental changes—“sensome genes”—during early development (*9*, *10*). Information from the local environment, in the form of cell surface molecules or secreted proteins, is processed by microglia, which, in turn, elicits a feedback response that can modulate brain development (*2*). Consequently, it is expected that even transient changes in microglial numbers during development can have long-lasting impacts on brain function (*11*, *12*). Perturbations in microglial number and development in rodents have been associated with changes in behavior akin to those observed in neurodevelopmental disorders such as autism spectrum disorder (*11*, *13*, *14*).

This impact, however, is not unidirectional. Recent studies have also provided insights into how differences in brain regions, such as the cerebral cortex, cerebellum and striatum, can shape and specify the number and development of microglia, leading to regional heterogeneity (*15*–*19*). More subtle heterogeneity has also been observed within the cerebral cortex, where various areas have different distributions of microglia (*20*, *21*). However, it is unclear how this heterogeneity arises during development.

The formation of cortical areas occurs early during development (*22*). During this time, the cerebral cortex is wrought with dynamic changes (*21*–*23*), including global fluctuations in neuronal numbers and region-specific changes in neuronal activity (*23*–*25*). Neuronal activity is critical for the development and maturation of functional circuits. As the activity of different cortical areas varies during development, they develop at different rates (*24*, *26*). Microglia can sense these changes (*10*, *27*), but it is currently unknown whether these fluctuations affect microglial numbers in the cerebral cortex. In this study, we investigated the role of pyramidal cells in regulating microglial numbers and function. We found that microglia are sensitive to changes in neuronal activity during a critical window of postnatal development in mice. Our experiments reveal a previously unknown mechanism through which activity-dependent secretion by pyramidal cells can fine-tune the development of microglial cells in the cerebral cortex.

## RESULTS

### Microglia developmental trajectories vary across cortical areas

**Fig. 1.**
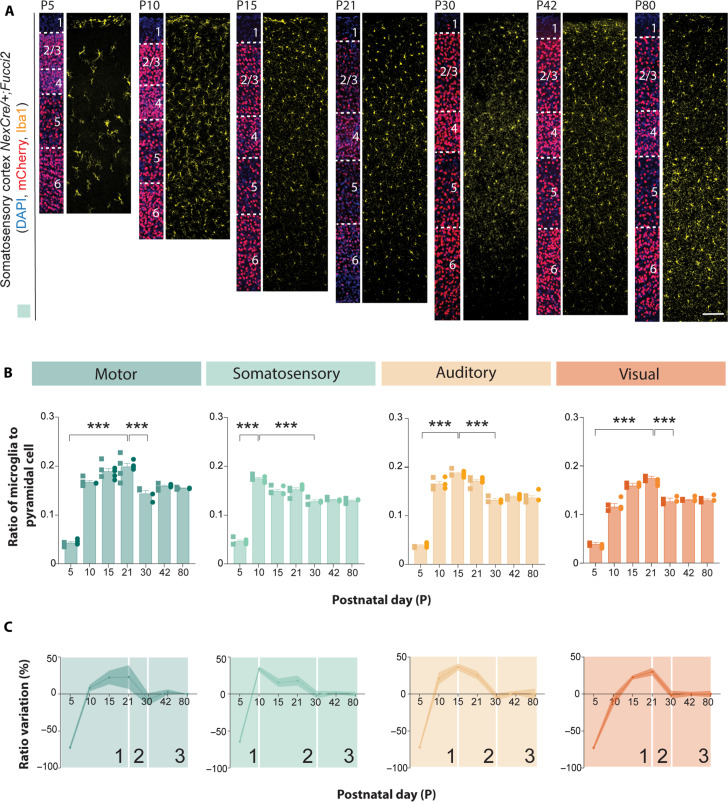
Sequential microglia developmental trajectories across cortical areas. (**A**) Coronal sections through the primary somatosensory cortex of *Nex^Cre/+^;Fucci2* mice at different developmental stages following immunohistochemistry against mCherry (red) and Iba1 (yellow). DAPI is shown for counterstaining (blue). (**B**) Ratio of microglia (Iba1+) to pyramidal cells (mCherry+) at different developmental stages in the motor (dark green), somatosensory (light green), auditory (yellow), and visual cortices (orange) [one-way ANOVA with Tukey multiple comparison, motor: *F* = 87.04, ***P < 0.0001 (P5 versus P21 and P21 versus P30), somatosensory: *F* = 249.9, ***P < 0.0001 (P5 versus P10 and P10 versus P30), auditory: *F* = 276.2, ***P < 0.0001 (P5 versus P15 and P15 versus P30), and visual: *F* = 335.4, ***P < 0.0001 (P5 versus P21 and P21 versus P30), *n* = 4 to 8 mice across different developmental stages). (**C**) Temporal percentage variation in ratio of microglia to pyramidal cells in motor (dark green), somatosensory (light green), auditory (yellow), and visual (orange) cortices. Data in (B) are shown as bar graphs, and the adjacent data points indicate the average cell density in each animal where square data points (left) indicate male mice whereas circle data points (right) indicate female mice. Scale bar, 100 μm.

To investigate whether microglia from various cortical areas develop differently during mouse postnatal development, we stained for the macrophage marker Iba1 in brain sections obtained from *NexCre/+* mice crossed with the reporter mice, *Fucci2* (Fig. 1A and fig. S1A). In these mice, Cre expression is controlled by the *NeuroD6* locus, which labels all cortical pyramidal cells. By comparing the changes in microglial numbers to those of pyramidal cells by examining their ratios after the completion of pyramidal cell death (*28*), we could compare alterations in microglial numbers across cortical areas despite differences in cortical thickness and developmental stages.

We observed an overall similar microglial developmental profile as previously reported (*29*, *30*). This profile can be divided into three stages (Fig. 1C). Phase 1 reflects the rapid increase in the microglia to pyramidal cells ratio due to microglia proliferation (Fig. 1 and figs. S1 and S2, A and C). This period is followed by phase 2, with a decline in the ratio due to the reduction in microglial proliferation (Fig. 1 and figs. S1 and S2A) and/or an increase in microglial cell death. This phase is accompanied by an increase in microglial cells that express homeostatic markers such as P2RY12 (fig. S2, B and D). By postnatal day (P) 30, the ratios of microglia to pyramidal cells are stabilized as the number of microglia is maintained at a steady state (phase 3, Fig. 1C and fig. S1) (*30*). Unexpectedly, unlike previous reports of sex differences in cortical microglia numbers (*10*, *31*), we did not observe any significant differences in microglia to pyramidal cell ratio between male and female pups at time points in which we have sufficient numbers of animals to properly power this comparison (Fig. 1B and table S1).

A closer examination of microglia developmental profiles revealed subtle but distinct differences across cortical areas (Fig. 1, B and C). For instance, the somatosensory cortex (S1) has the shortest proliferation phase (Fig. 1, light green, phase 1), followed by the auditory (Fig. 1, yellow), visual (Fig. 1, orange), and motor cortices (Fig. 1, dark green). Phase 2 is, in turn, much longer in the somatosensory cortex compared to the other cortices. These subtle changes are also reflected by the density of microglia expressing the proliferating marker Ki67 (fig. S2, A and C) and homeostatic marker such as P2RY12 (fig. S2, B and D). However, by P30, cortical microglia from different areas reach a steady state, and their numbers are maintained throughout adulthood (*30*). Together, these experiments revealed that each cortical area has a unique microglial developmental profile reminiscent of the order of maturation of the cortical regions during development and does not follow a rostral-to-caudal development profile (*24*, *26*).

### Bidirectional manipulation of pyramidal cell activity regulates microglia numbers

**Fig. 2.**
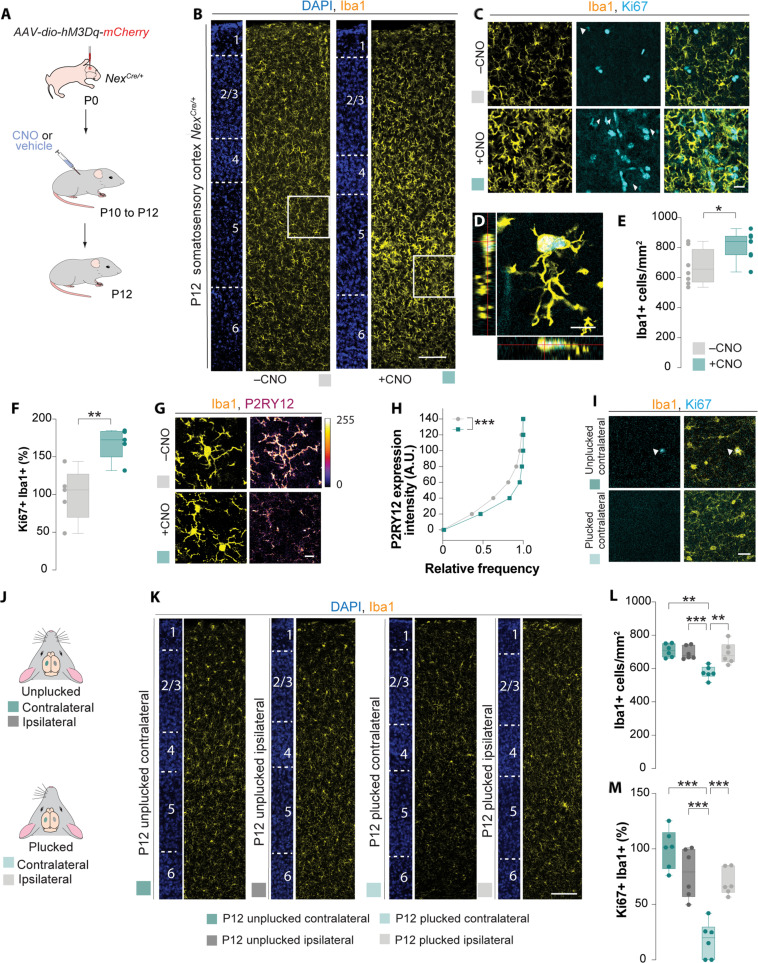
Modulation of pyramidal cell activity regulates microglial number. (**A** and **J**) Schematic of experimental design. (**B**, **C**, **D**, and **G**) Coronal sections through S1BF of *Nex^Cre/+^* mice at P12 injected with *hM3Dq-mCherry* virus followed by vehicle or CNO treatment immunostained for Iba1 (yellow), Ki67 (cyan), and P2RY12 (fire lookup table). DAPI is shown for counterstaining (blue). White box showed amplified area in (C). White arrowheads depict Ki67+ Iba1+ cells. (D) Maximum projection intensity of Iba1+ cells together with the corresponding orthogonal sections. (**E**) Quantification of Iba1+ cells at P12. Two-tailed Student’s unpaired *t* test, *P = 0.0155, *n* = 8. (**F**) Quantification of the percentage of Ki67+ Iba1+ cells in L2/3 at P12. Two-tailed Student’s unpaired *t* test, **P = 0.0059, *n* = 5. (**H**) Cumulative distribution of P2RY12 intensity in layer 2/3 S1BF in Iba1+ cells at P12. Kolmogorov-Smirnov test, ***P < 0.0001; *n* = 199 (vehicle) and 183 cells (CNO) from four mice from each group. A.U., arbitrary units. (**I** and **K**) Coronal sections through S1BF of contralateral and ipsilateral hemispheres of CD1 with unplucked or plucked whiskers immunostained for Iba1 (yellow) and Ki67 (cyan). DAPI is shown for counterstaining (blue). White arrowheads depict Ki67+ Iba1+ cells. (**L**) Quantification of Iba1+ cells at P12. One-way ANOVA with Tukey multiple comparisons, *F* = 10.40, **P = 0.0015 (contralateral unplucked versus contralateral plucked) and ***P = 0.0004 and **P = 0.002 (ipsilateral plucked versus contralateral plucked), *n* = 6. (**M**) Quantification of percentage Ki67+ Iba1+ cells at P12. One-way ANOVA with Tukey multiple comparisons, *F* = 23.52, ***P < 0.0001 (contralateral unplucked versus contralateral plucked, ipsilateral unplucked versus contralateral plucked) and ***P = 0.0003 (ipsilateral plucked versus contralateral plucked), *n* = 6. For box plots, data points indicate the average cell density in each animal. Scale bars, 100 μm [(B) and (K)], 20 μm [(C) and (G)], and 10 μm [(D) and (I)].

As cortical areas exhibit different levels of neuronal activity during early mouse postnatal development (*24*, *32*), we examined whether changes in pyramidal cell activity can modulate microglial development. To this end, we took a chemogenetic approach based on designer receptors exclusively activated by designer drugs (DREADDS) to transiently increase neuronal activity after the period of developmental programmed neuronal cell death (*28*, *33*). As the use of DREADDS requires the use of ligands such as clozapine-*N*-oxide (CNO), we first confirmed that injection of CNO alone has no impact on microglial numbers (fig. S3). Next, we injected the S1 of P0 *NexCre/+* mice with adeno-associated viruses (AAVs) encoding mutant G protein–coupled receptors (hM3Dq) to enhance neuronal activity following administration of the ligand CNO (Fig. 2A and fig. S4A). We have previously shown that 4 days of altered neuronal activity during the critical period of interneuron development was sufficient to modulate their survival (*28*, *34*). Consequently, to determine whether changes in neuronal activity can affect microglial numbers acutely, we administered CNO to pups twice daily between P10 and P12, after the completion of neuronal cell death in order not to alter neuronal numbers (*25*). As no sex differences were observed in our experiments (Fig. 1B), we combined both male and female datasets for all subsequent analyses. We evaluated the impact of this manipulation on the distribution of microglia at P12 (Fig. 2, B to E, and fig. S4). We found that elevation of neuronal activity increased microglial density (Fig. 2E) despite the reduction in proliferating microglia and/or increase in microglial cell death during this phase of microglia development (phase 2, fig. S2A). We observed a significant increase in microglia density in layer 6 (fig. S4B). Next, we determined whether similar regulation of microglial numbers by pyramidal cell activity is extended to other cortical areas. To this end, we injected the V1 of P0 *NexCre/+* mice with hM3Dq. Here, we observed a similar increase in microglia density with no impact on microglial distribution (fig. S5). Together, this suggests that changes in pyramidal cell activity can regulate microglial numbers independent of their cortical regions.

To confirm the impact of altered neuronal activity on microglial numbers, we used an alternative nonviral methodology to alter levels of neuronal activity. The whisker-plucking paradigm has been previously shown to reduce neuronal activity in the somatosensory cortex without inducing large-scale changes in the cortical cytoarchitecture (*35*, *36*). In brief, we plucked the whiskers from the right side of the snout between P10 and P12 and evaluated the distribution of microglia using Iba1 staining (Fig. 2, J to M). A significant decrease in microglia density was observed in the contralateral hemisphere of the whisker-plucked mice, which coincides with the region having reduced neuronal activity (Fig. 2L) without affecting its distribution (fig. S4C). This observation suggests that bidirectional manipulation of neuronal activity through viral or nonviral manipulations can alter microglial density during early mouse postnatal development.

We next investigated whether neuronal activity can modulate other aspects of microglial development by examining the expression of genes that are up-regulated or down-regulated upon maturation (*37*). We first determined whether elevation of pyramidal cell activity could alter the proportion of proliferating microglia in the somatosensory cortex by staining for the proliferating marker Ki67 (Fig. 2, C and D). We observed a significant increase in the percentage of Ki67+ Iba1+ cells when we elevated neuronal activity (Fig. 2F), suggesting that elevation of neuronal activity can slow the decline in microglia proliferation. A complementary decrease in the percentage of proliferating microglia was also observed when we dampened neuronal activity (Fig. 2, I and M). We also observed an increase in Ki67+ staining in non-Iba1+ cells when we increased neuronal activity (Fig. 2C). This increase in Ki67+ Iba1− cells could be attributed to oligodendrocyte and endothelial cells as previous studies have shown that these cells are also sensitive to changes in neuronal activity (*38*, *39*). We wondered whether astrocytes too are similarly sensitive to changes in neuronal activity. To this end, we stained for the astrocytic marker, Sox9 (*40*) (fig. S6). Similar to other cell types, transient increase in neuronal activity during the second week of mouse postnatal development significantly increased astrocytic density (fig. S6B) but not their distribution (fig. S6C).

We next examined the expression of P2RY12 in L2/3 of the somatosensory cortex (Fig. 2G). In contrast to the increase in proliferating microglia, we observed a significant reduction in the P2RY12 immunofluorescence intensity following activating DREADDS (Fig. 2H). Together, this demonstrates that alterations in neuronal activity alter microglial numbers during the second week of mouse postnatal development and potentially alter their developmental trajectories (phases 1 and 2).

### Manipulation of pyramidal cell activity during development has a long-term impact on microglial numbers

We next investigated whether these acute manipulations have long-term consequences on microglia. To this end, we used the same chemogenetic approach that we have previously used (*28*, *34*) and injected pups twice daily from P10 to P13 with CNO. We examined the distribution of microglia at P42 (fig. S7A). We observed a significant increase in microglial density at P42 when we increased neuronal activity during the second week of mouse postnatal development (fig. S7, C and D) but not their distribution (fig. S7E). In addition, we also reconstructed microglia in three dimensions (3D) (fig. S7B) and observed a significant reduction in cellular volume (fig. S7F) and reduced complexity based on Scholl analysis (fig. S7G). This suggests that acute manipulation of neuronal activity during the second week of mouse postnatal development has a long-term impact on microglial numbers and morphology.

We next investigated whether alteration in neuronal activity during different phases of microglia development can have a similar long-term impact on microglial numbers. To test this, we injected the S1 of *NexCre/+* mice with AAVs encoding hM3Dq to modify the activity of pyramidal cells during the three previously described phases: Phase 1 (P7 to P10), phase 2 (P15 to P18), and phase 3 (P28 to P31) (Fig. 1 and fig. S8A). An increase in neuronal activity in phases 1 and 2 led to a significant increase in microglial numbers (fig. S8, B, C, E, and F) but not in phase 3 (fig. S8, H and I). Regardless, of the time frame of CNO manipulation, no impact on microglial distribution was observed (fig. S8, D, G, and J). This suggests that, once microglial numbers are stabilized (phase 3), alterations in pyramidal cell activity have no impact on microglial numbers (fig. S8, H and I). Therefore, cortical microglia appear sensitive to changes in neuronal activity during a limited time window during mouse postnatal development.

### Microglia are sensitive to changes in patterns of spontaneous activity during mouse postnatal development

**Fig. 3.**
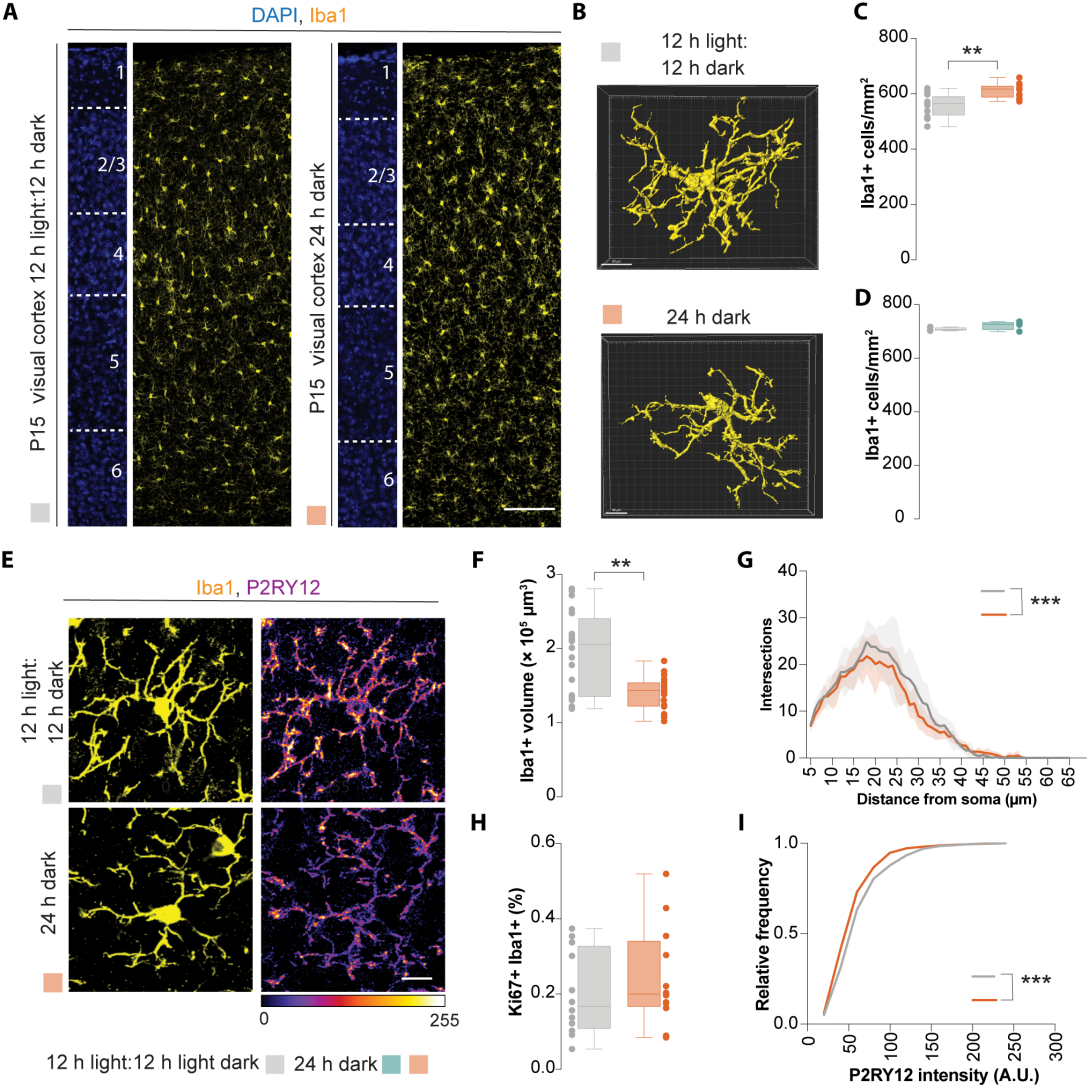
Delayed in changes of spontaneous activity patterns alters microglia numbers. (**A** and **E**) Coronal sections through the visual cortex of CD1 mice at P15 reared in standard light: dark cycle (12-hour light:12-hour dark) or in complete darkness (24-hour dark) immunostained for Iba1 (yellow) and P2RY12 (fire lookup table). DAPI is shown for counterstaining (blue). (**B**) 3D microglia reconstruction in mice. (**C**) Quantification of Iba1+ cells in the visual cortex at P15. Two-tailed Student’s unpaired *t* test, **P = 0.0010, *n* = 12. (**D**) Quantification of Iba1+ cells in the somatosensory cortex at P15. Two-tailed unpaired Student’s *t* test, *n* = 4. (**F**) Quantification of Iba1+ cell volume at P15. Mann-Whitney test, **P = 0.0024 (control: *n* = 20 cells, complete darkness *n* = 19 cells). (**G**) Scholl analysis of microglia. Two-way ANOVA [*F_Group_*(1,336) = 12.36, ***P = 0.0005), *n* = 4. (**H**) Quantification of the percentage of Ki67+ Iba1+ cells at P15. Two-tailed unpaired Student’s *t* test. (**I**) Cumulative distribution of P2RY12 intensity in layer 2/3 visual cortex in Iba1+ cells at P15. Kolmogorov-Smirnov test, ***P < 0.0001; *n* = 668 cells (control) and 711 cells (complete darkness) from four mice from each group. For box plots, the adjacent data points indicate the average cell density in each animal. Scale bars, 100 μm (A), 50 μm (B), and 10 μm (E).

We have shown that modulation of neuronal activity alters microglial numbers during a critical developmental window in a similar manner across all cortical areas. Coincidentally, this period of mouse postnatal development is marked by changes in the patterns of spontaneous activity (*23*, *24*). We hypothesized that, because cortical microglia are influenced by changes in neuronal activity (*27*), they should be able to detect and react to these changes. To test this hypothesis, we shifted our analysis to the visual cortex for the following reasons: It has been previously shown that dark-rearing pups for up to 3 days after eye opening delays the decorrelation of network activity (*41*), whereas this effect has not been reported in the somatosensory cortex using the whisker-plucking paradigm (*42*). Furthermore, we have shown that increasing neuronal activity in the visual cortex leads to a similar increase in microglial density as seen in the somatosensory cortex (fig. S5). Consequently, to study the potential impact of altered patterns of spontaneous activity on microglia numbers, we reared our pups in either standard light-dark cycles (e.g., 12-hour light followed by 12-hour dark) or in constant darkness (e.g., 24-hour dark). We then examined the distribution of microglia in the visual cortex 3 days after eye opening (~P15). We observed a significant increase in microglial numbers in the visual cortex (Fig. 3, A and C), but not in the somatosensory cortex (Fig. 3D), of mice reared in constant darkness compared to controls. In addition, we did not observe any changes in microglial distribution (fig. S9A).

We next reconstructed the morphology of microglia in 3D (Fig. 3B) and observed a reduction in microglial volume (Fig. 3F) and complexity based on Scholl analysis (Fig. 3G). We evaluated the percentage of proliferating microglia in L2/3 of the visual cortex but did not observe a significant difference (Fig. 3H). In addition, we assessed the intensity of P2RY12 expression as an indication of microglia maturation (*37*) and observed a substantial decrease in immunofluorescence intensity (Fig. 3I). Last, we also examined the long-term impact of delayed decorrelated activity in the visual cortex during development. To this end, we reared our pups in either standard light-dark cycles or in constant darkness up to 3 days after eye opening (~P15), before rearing them in normal light-dark cycles. We next evaluated the distribution of microglia in the adult brain (~4 months old). Here, we observed a significant increase in microglia density but not their distribution (fig. S9, B and C). Together, this suggests that microglia are tuned to the changes in neuronal activity when pups are reared in complete darkness, possibly through changes in spontaneous activity (*41*). These changes can alter microglial numbers and specific aspects of their development.

### Pyramidal cells regulate microglia numbers via activin signaling

**Fig. 4.**
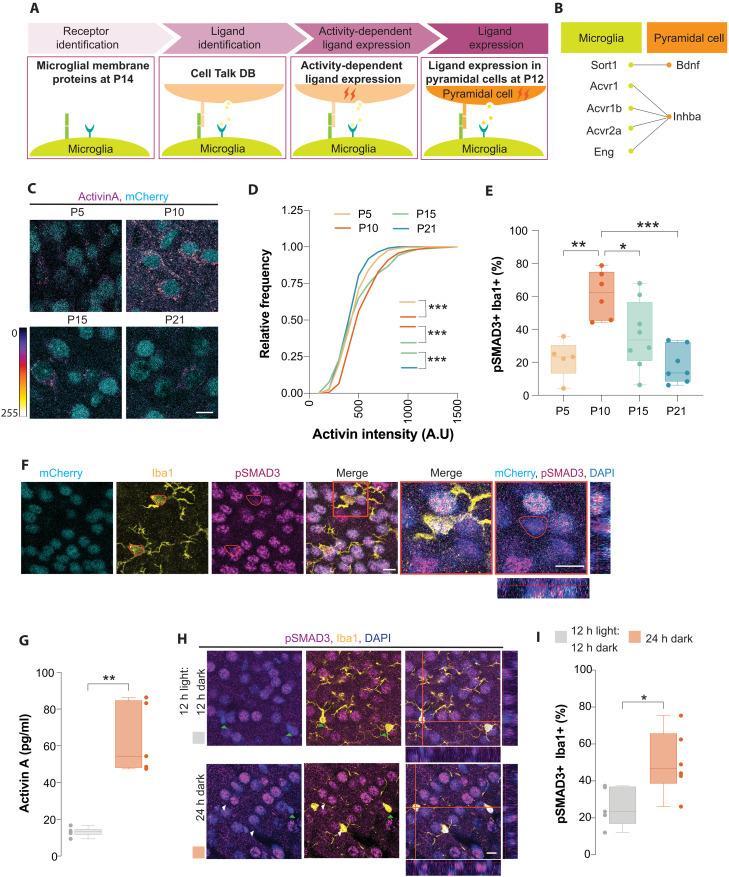
Changes in spontaneous activity pattern alter activity-dependent Activin A secretion. (**A**) Schematic of experimental design. (**B**) List of potential candidates. (**C**) Coronal sections through the primary visual cortex of *Nex^Cre/+^;Fucci2* mice at different developmental stages following immunohistochemistry against Activin A (fire lookup table) and mCherry (cyan). (**D**) Cumulative distribution of Activin A intensity in layer 2/3 visual cortex at different developmental time window. Kruskal-Wallis test, ***P < 0.0001; P5: *n* = 668 cells, P10: *n* = 366 cells, P15: *n* = 257 cells, and P21: n = 236 cells from four mice from each group. (**E**) Quantification of the percentage of pSMAD3+ Iba1+ cells in L2/3 visual cortex at P5 (*n* = 5), P10 (*n* = 6), P15 (*n* = 8), and P21 (*n* = 7). One-way ANOVA with Tukey multiple comparison (*F* = 9.575, *P = 0.035, **P = 0.002, ***P = 0.0003). (**F** and **H**) Coronal sections through the primary visual cortex of *Nex^Cre/+^;Fucci2* (F) and CD1 (H) mice at P10 following immunohistochemistry against Iba1 (yellow), pSMAD3 (magenta), and mCherry (cyan). DAPI is shown for counterstaining (blue). Last two panels in (F) are higher magnification of Iba1+ pSMAD3+ cells highlighted in the red box. Final panel is accompanied by the orthogonal *z*-stack view of pSMAD3+ cell. In (H), green arrowheads depict Iba1+ pSMAD3− cells, and white arrowheads depict Iba1+ pSMAD3+ cells. (**G**) Quantification of Activin A levels using ELISA from visual cortices. Mann-Whitney test, **P = 0.0043, control: *n* = 6; complete darkness: *n* = 5. (**I**) Quantification of the percentage of pSMAD3+ Iba1+ cells in layer 2/3 visual cortex. Two-tailed unpaired Student’s *t* test, *P = 0.025, *n* = 5. For box plots, data points indicate the average cell density or Activin A levels in each animal. Scale bars, 10 μm.

We developed a bioinformatic pipeline using published transcriptome datasets that are specific to the mouse brain and when possible the cerebral cortex to identify the molecular mechanism through which pyramidal cells regulate microglia numbers and development (Fig. 4A) (*37*, *43*–*47*). First, we identified 1317 genes coding for potential receptors and membrane proteins expressed by microglia at P14 (*37*). We then identified potential ligands of these receptors and membrane proteins using the ligand-receptor databases, CellTalkDB (*46*) and CellPhoneDB in the adult brain (*47*). We narrowed the number of potential microglial genes to 121 using this approach (table S2). Since neuronal activity is critical in regulating microglial numbers, we further reduced the list of possible candidates to genes whose expression is known to be modulated by changes in pyramidal cell activity (*45*). Finally, we further narrowed down our list of ligand-receptor pairs to include only genes expressed by pyramidal cells at P12 (*43*). This process identified five potential ligand-receptor pairs (Fig. 4B). Out of this list, we decided to focus on the Activin A signaling pathway, a member of the transforming growth factor–β (TGF-β) family, as four of five of these microglial receptors are Activin A receptors.

To confirm the validity of our analysis, we first examined the expression of Activin A at different developmental time points by measuring immunofluorescence intensity of pyramidal cells located in the L2/3 of the visual cortex (Fig. 4C and fig. S10). We observed that the expression of Activin A increased transiently and peaked in the visual cortex before eye opening (~P12) (Fig. 4, C and D). Notably, the expression of Activin A downstream signaling proteins, such as pSMAD3, also transiently increased and peaked in L2/3 microglia before eye opening (Fig. 4, E and F). The decrease in Activin A levels in the visual cortex after eye opening corresponds to the time window in which pyramidal cells are known to have decorrelated firing (*41*). We therefore hypothesized that changes in spontaneous activity patterns alter the activity-dependent secretion of Activin A. Consequently, any manipulations that perturb patterns of spontaneous activities may have a direct consequence on Activin A levels. To test this idea, we measured the levels of Activin A in the visual cortex isolated from pups reared under normal conditions and in constant darkness using enzyme-linked immunosorbent assay (ELISA). We observed a significant increase in Activin A levels in animals reared in constant darkness (Fig. 4G). Consistently, we observed a substantial increase in the percentage of microglia expressing pSMAD3 in the L2/3 of the visual cortex of animals reared in constant darkness (Fig. 4, H and I). This experiment suggests that rearing mice in complete darkness after eye opening can modulate the levels of Activin A within the brain parenchyma, which, in turn, can be detected by microglia.

### Deregulation of Activin A signaling alters microglia number and function

To determine whether altering Activin A expression during development can modulate microglial cells, we generated a conditional knockdown strategy using Cre-dependent AAV vectors that express microRNA-based short hairpin RNA (shRNA) against *Inhba,* which encodes for Activin A. We first confirmed the efficacy of shRNAs in down-regulating *Inhba* in vivo (fig. S11). We next examined whether knocking down Activin A in pyramidal cells would interfere with its role in noncell autonomous modulation of microglial numbers and development. Here, we injected either the control or *Inhba* shRNA into the visual cortex of *NexCre/+* mice and reared the animals in a standard light-dark cycle or constant darkness (fig. S12A). We found the down-regulation of *Inhba* in pyramidal cells was sufficient to prevent the increase in microglial numbers induced by dark rearing (fig. S12, B and D) but not alter their distribution (fig. S12F). Consistently, we also observed a rescue in the expression levels of P2RY12 (fig. S12, C and H). We also studied the impact of knocking down *Inhba* in pyramidal cells on microglia morphology. By reconstructing L2/3 microglia from the visual cortex in 3D (fig. S12B), we observed that the knockdown of *Inhba* in pups reared in constant darkness led to a restoration of microglia volume and complexity like those in a standard light-dark cycle (fig. S12, E and G). Together, these results strongly suggest pyramidal cells can regulate microglial numbers and development through activity-dependent Activin A release during mouse postnatal development.

**Fig. 5.**
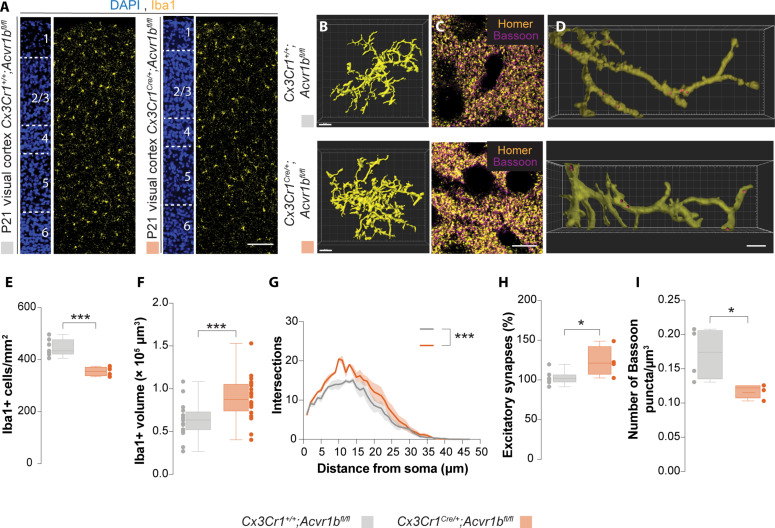
Altered Activin A signaling in macrophages induces changes in microglial numbers and function. (**A** and **C**) Coronal sections through the primary visual cortex of *Cx3Cr1^+/+^;Acvr1b*^*f/f*`^ and *Cx3Cr1^Cre/+^;Acvrb^f/f^* mice at P21 following immunohistochemistry for (A) Iba1 (yellow) and (C) Homer (yellow) and Bassoon (magenta). DAPI is shown for counterstaining (blue). (**B** and **D**) 3D morphological reconstruction of (B) microglia and (D) microglia processes from the primary visual cortex at P21. Red circles depict encased Bassoon puncta within a microglia process. (**E**) Quantification of Iba1+ cells in the visual cortex at P21. Two-tailed unpaired Student’s *t* test, ***P = 0.0007. Control: *n* = 7; mutant: *n* = 4. (**F**) Quantification of Iba1+ cell volume at P21 from four mice per group. Two-tailed unpaired Student’s *t* test, ***P = 0.0009, *n* = 20 cells. (**G**) Scholl analysis of microglia. Two-way ANOVA [*F_Group_*(1,282) = 64.42, ***P < 0.0001), *n* = 4. (**H**) Quantification of the percentage of colocalized excitatory synapses in L2/3 at P21. Two-tailed unpaired Student’s *t* test, *P = 0.04. Control: *n* = 7; mutant: *n* = 4. (**I**) Quantification of Bassoon puncta encased within an Iba1+ process at P21. Two-tailed unpaired Student’s *t* test, *P = 0.04, *n* = 4. For box plots, the adjacent data points indicate the average cell density, percentage of excitatory synapses, or number of Bassoon puncta encased within an Iba1+ process in each animal. Scale bars, 100 μm (A), 30 μm (B), 10 μm (C), and 3 μm (D).

Next, we investigated the consequences of decoupling pyramidal cell regulation of microglial numbers and development. To this end, we abolished activin signaling in all macrophages, including microglial cells, by targeting *Acvr1b*. This gene encodes an activin co-receptor required for all intracellular signal transduction downstream of Activin A in *Cx3Cr1+* cells (*Cx3Cr1^Cre/+^;Acvr1b^f/f^*) (*48*). We first confirmed that Acvr1b is deleted specifically in microglia by examining the colocalization of Iba1 and Acvr1b signal after immunofluorescence staining (fig. S13). We then examined the distribution of microglia in the visual cortex and somatosensory cortex. Here, we observed a significant decrease in microglial numbers (Fig. 5, A and E, and fig. S15) but without alteration in their distribution (figs. S14 and S15) at P21 when developmental synaptic pruning has completes (*2*). To further confirm the role of Activin A in regulating microglia numbers, we depleted microglia using the CSF1R inhibitor PLX5622 in *Cx3Cr1^Cre/+^;Acvr1b^f/f^* mice during the time window in which microglia are sensitive to changes in neuronal activity. To this end, we injected once daily PLX5622 (50 mg/kg), subcutaneously between P10 and P17 (fig. S16B). We first confirmed the efficacy of PLX5622 in depleting microglia in our treatment regime. In our hands, we observed a 60% decrease in microglia density when postnatal pups were treated for 1 week (fig. S16C). Next, we determined the impact of altered Activin A signaling in microglia repopulation in the cerebral cortex. It has been previously shown that, by 3 weeks after the completion of CSF1R inhibitor treatment, the remaining microglia is able to repopulate the cerebral cortex to similar levels prior to depletion (*49*). Similar to previous works, we observed a return to baseline levels of microglia, 3 weeks after PLX5622 treatment in control (*Cx3Cr1^+/+^;Acvr1b^f/f^*) mice (fig. S16F). However, for the *Cx3Cr1^Cre/+^;Acvr1b^f/f^* mutant mice, a reduction in microglia density compared to controls persisted at 3 weeks after the last PLX5622 treatment.

We next reconstructed microglia in 3D and observed an increase in microglial volume (Fig. 5, B and F). Furthermore, we also observed an increase in microglial complexity via Scholl analysis in conditional mutants (Fig. 5G). This result complements the data from animals reared in constant darkness (Fig. 3, F and G). Next, we investigated the impact of decoupling of pyramidal cell–microglia interactions during development on microglia function. To this end, we evaluated the number of excitatory synapses in L2/3 of the visual cortex by staining for the presynaptic protein, Bassoon, and the postsynaptic protein, Homer (Fig. 5C). Here, we observed a significant increase in the percentage of colocalized pre- and postsynaptic puncta in the conditional mutants, suggesting an increase in excitatory synapses when we prevent the regulation of microglia by pyramidal cells (Fig. 5H). We hypothesized that this increase in excitatory synapses is due to an alteration in the capacity of microglia to engulf excitatory synapses. To study this, we reconstructed L2/3 microglia processes in the visual cortex in 3D and examined the number of presynaptic puncta encased within the microglial processes (Fig. 5D). We observed a reduction in the number of Bassoon+ puncta/μm^3^ encased within the microglial processes when we decoupled the regulation of microglia development by pyramidal cells (Fig. 5I).

### Decoupling of microglia-pyramidal communication leads to behavior alterations

**Fig. 6.**
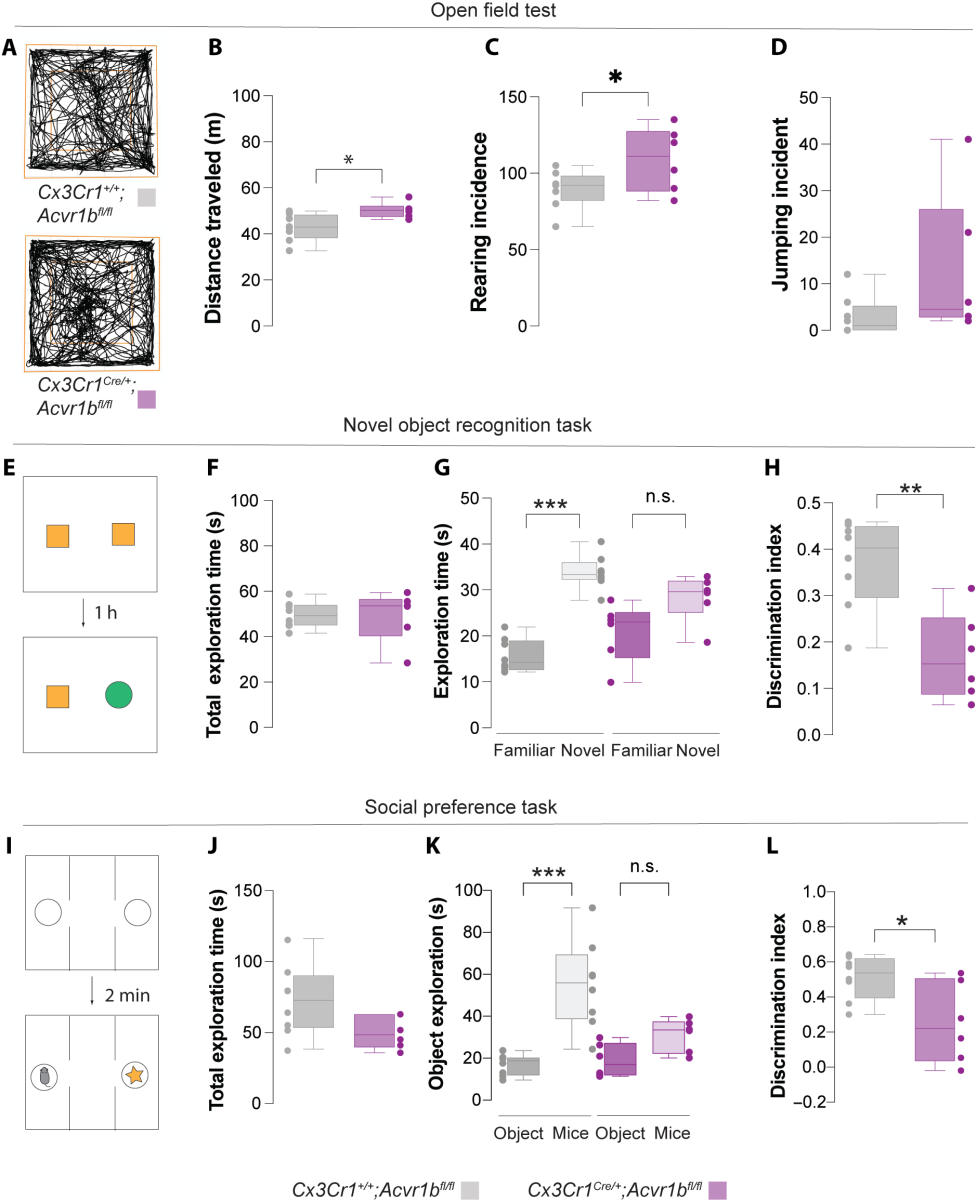
Decoupling of pyramidal cell–microglia communication alters cortical function. (**A**) Representative track for the open field task. (**B**) Total distance traveled in the open field arena with 10-min interval. Two-tailed unpaired Student’s *t* test, *P = 0.017. Control: *n* = 8; mutant: *n* = 6. (**C**) Number of rearing incidences in the open field arena with 10-min interval. Two-tailed unpaired Student’s *t* test, *P = 0.048. Control: *n* = 8; mutant: *n* = 6. (**D**) Number of jumping incidences in the open field arena with 10-min interval. (**E** and **I**) Schematic of experimental design. (**F**) Total object exploration time in the open field arena with 6-min interval. Control: *n* = 8; mutant: *n* = 6. (**G**) Familiar and novel objects exploration time. One-way ANOVA with Tukey multiple comparison (*F* = 22.58, ***P < 0.0001). Control: *n* = 8; mutant: *n* = 6. n.s., not significant. (**H**) Discrimination index for novel objects. Two-tailed unpaired Student’s *t* test, **P = 0.021. Control: *n* = 8; mutant: *n* = 6. (**J**) Total stimulus exploration time in the open field arena with 6-min interval. Control: *n* = 8; mutant: *n* = 6. (**K**) Social and inanimate object stimuli exploration time. One-way ANOVA with Tukey multiple comparison (*F* = 14.41, ***P < 0.0001). Control: *n* = 8; mutant: *n* = 6. (**L**) Discrimination index of stimulus. Two-tailed unpaired Student’s *t* test, *P = 0.018. Control: *n* = 8; mutant: *n* = 6. For box plots, data points indicate the average measurement for each animal.

We next sought to determine whether decoupling of pyramidal cell–microglia communication through microglia-specific ablation of Activin A signaling have a long-term consequence on cortical function. To this end, we performed a battery of behavioral tests on control (*Cx3Cr1^+/+^;Acvr1b^f/f^*) and *Cx3Cr1^Cre/+^;Acvr1b^f/f^* conditional mutant mice aged between 2 and 4 months old. We first examined the spontaneous locomotor activity using an open field test where mice were placed in an arena and observed for 10 min (fig. S17A). Although both genotypes showed no difference in their preferences to the outer or inner zones (fig. S17C), conditional mutant mice showed higher locomotor activity than controls (Fig. 6, A and B). We also studied other behaviors such as rearing and jumping incidences (Fig. 6, C and D). Although a trend toward an increase in incidence of jumping was observed in conditional mutants, it is not significantly different between genotypes (Fig. 6D). Conditional mutants demonstrated a significantly higher incidence of rearing (Fig. 6C), limited to male conditional mutants (fig. S18). No other sex differences were observed in all our behavioral tests and parameters.

We next subjected the animals to a light-dark task. Here, mice were placed in an arena consisting of two chambers—a light chamber and a dark chamber that is connected by a small archway (fig. S17D)—and observed for 10 min. Consistent with our results from the open field tasks (fig. S17C), no differences in genotypes were observed in the amount of time the mice spent in either chamber (fig. S17E) nor entries into the dark chamber (fig. S17F). This, together with the open field results, suggests that ablation of Activin A signaling in microglia do not lead to alterations in anxiety-related behavior.

We next studied the impact of dysregulation of pyramidal cells and microglia on memory tasks such as the novel object recognition task. Here, mice were first habituated in the arena with two similar objects for 10 min (familiar objects). After 1 hour, these mice were reintroduced into the arena with one familiar object together with a novel object and observed for a further 6 min (testing phase, Fig. 6E). Both control and conditional mutant mice showed no differences in total object exploration time, suggesting that both genotypes have similar levels of motivation to perform the task (Fig. 6F). Notably, conditional mutant mice demonstrated no preference in exploring either object whereas the control mice preferentially explored the novel object during the testing phase (Fig. 6, G and H). This highlights that ablation of Activin A signaling in microglia can lead to a deficit in short-term memory.

We next tested the mice in a three-chambered social preference task (Fig. 6, I to L). Mice were habituated to the apparatus for 10 min, followed by a 6-min testing period in which a novel mouse of similar age and sex were placed into one of the perforated Plexiglas cylinders in the outer chamber, whereas the other contained an inanimate object (Fig. 6I). Similar to the novel object recognition task, no differences in active exploration time of the stimuli were observed between genotypes (Fig. 6J). Control mice showed a preference for the stimulus animal, but notably, the conditional mutants did not (Fig. 6, K and L). Together, these results suggest that dysregulation of pyramidal cells and microglia signaling during development alters microglial function and how cortical circuits are built during development leading to hyperactivity, memory deficits, and social impairments.

## DISCUSSION

Our study highlights the pivotal role of pyramidal cell activity in establishing microglia numbers and function during a critical window in mouse postnatal development. Using both viral and nonviral approaches, we observed a bidirectional regulation of microglia numbers and development following alterations in pyramidal cell activity. We found that changes in spontaneous activity patterns during the first few weeks of postnatal development can alter microglial numbers and development through changes in activity-dependent Activin A secretion. Together, this suggests that the maturation of cortical areas, driven by changes in spontaneous activity, is instructive in microglia development and the establishment of their final numbers. The decoupling of this instructive role of pyramidal cells led to dysregulation in microglial function and numbers in the cerebral cortex, ultimately affecting cortical function. Our findings illuminate the fundamental role of pyramidal cells in sculpting microglia distribution and function in the mouse cerebral cortex.

It has been previously shown that microglia populate the cerebral cortex following a rostral-to-caudal gradient during development (*50*). In this study, we observed subtle differences in microglial developmental profiles independent of the rostral-to-caudal gradient. We show that the microglial developmental profile is reminiscent of the maturational order of cortical areas (*24*, *26*). Cortical areas known to develop and mature first, such as the somatosensory cortex, contain microglia that mature first compared to neighboring areas, such as the motor, auditory, and visual cortices.

This in sync development between microglia and maturation of cortical areas supports the idea that establishing microglia identity and function involves two key components: a developmentally regulated core transcriptional program (*10*, *51*, *52*) and an environmentally driven transcriptional program (*10*, *53*). While the core transcriptional program is critical in specifying the early identity of microglia, the environmentally driven program ensures the functional relevance of microglia to a specific phase of development and its local niches (*15*–*19*). Here, we demonstrate that the environmental cues also consist of the dynamic changes in neuronal activity during the first few weeks of mouse postnatal development (*23*, *24*, *26*). Our study indicates that microglia can sense these changes in neuronal activity, including spontaneous activity patterns, and adjust their developmental rate accordingly. The subtle variation in the rate of microglia development from different cortical areas evidences this. As microglia are known to perform a plethora of functions during development beyond their roles as immune sentinel and effectors (*2*, *8*), it is conceivable that the local environment provides instructive cues to shape microglial development to meet the demands of their local niches.

The types of signals that microglia receive can be broadly divided into two categories. First, broad, generic cues shape microglia’s development in an all-or-none manner. This includes microglia survival cues such as CSF-1 (colony-stimulating factor 1) and IL-34 (interleukin-34) and maturational cues such as TGF-β (*54*–*57*). Such cues ensure that mature microglia in the brain parenchyma can perform global functions by acting as immune sentinels and effectors. However, recent works have also highlighted substantial microglial heterogeneity between cortical areas (*20*, *21*). Consequently, each cortical area would have differing cellular compositions and activity demands that might not be sufficiently met with such global instructive cues (*15*–*19*). The presence of a second set of more localized cues would instead provide spatial and temporal modulation that would help shape microglia development to meet the demands of the local niches at the time. For instance, a recent work has uncovered the spatial cues within the cerebral cortex that help organize the laminar distribution of microglia at the end of the second week of mouse postnatal development (*58*). Through the expression of pyramidal cell layer–specific cues, the pyramidal cell–microglia interaction not only redistributes microglia across the laminar but also regulates their gene expression/states to meet specific laminar demands.

Our work provides evidence that these localized cues go beyond the spatial information. We provide a mechanism in which the local environment can shape microglia development dynamically along the temporal axis. Harnessing activity-dependent secretion of proteins such as Activin A (*45*, *59*) offers a mechanism for the convergence of two independent systems to ensure that their developments are perfectly aligned. Activin A is a member of the TGF-β signaling pathway (*48*). Unlike most TGF-β family proteins, the expression and secretion of Activin A is activity dependent (*45*, *59*). Consequently, changes in the frequency and amplitude of neuronal activity can affect Activin A secretion. We have demonstrated that delaying the transition from synchronous to decorrelated activity in the visual cortex alters Activin A levels in this region (Fig. 4G). Consequently, it is conceivable that, during brain development, marked changes in spontaneous activities coupled with the relatively short half-life of Activin A (*23*, *24*, *60*) provide a dynamic input to regulate specific transcriptional programs in microglia.

Although the inhibition of Activin A expression in pyramidal cells can suppress alterations in microglial numbers and maturation in our hands, other activity-dependent cues likely regulate microglia development. Different manipulations of neuronal activity can have diverging impacts on microglia development. While manipulations of neuronal activity that involve dramatic changes, such as chemogenetics and whisker plucking, lead to an alteration in the percentage of proliferating microglia (Fig. 2, D and I), more subtle manipulations, such as by rearing in complete darkness after eye opening, did not (Fig. 3H). This suggests that there are potentially other cues that can modulate different aspects of microglia development that was not identified in our pipeline (Fig. 4). As neuronal activity is associated with the release of other neurotransmitters and mitogens such as ATP (adenosine 5′-triphosphate), glutamate, and GABA (γ-aminobutyric acid) (*61*, *62*), combinatorial temporal cues can drive specific transcriptional changes and tune microglia development to the demands of their local niches.

Emerging evidence has identified the role of microglia in synaptic wiring and function in neuronal circuits (*8*, *63*). The capacity of microglia to perform these functions relies on their ability to survey and respond to their environment (*9*, *10*). This is influenced by microglial density, their ramification indices and dynamics, and their gene expression profiles (*10*, *51*, *64*). Although we have shown that ablation of activin signaling in microglia is able to induce behavior impairments, recent works have also shown transient expression of *Cx3Cr1* in other cortical cells during development (*65*, *66*). This suggests that, in the constitutively expressed Cre in our *Cx3Cr1^Cre/+^;Acvr1b^f/f^* mice experiment, activin signaling is likely ablated in other cell types besides microglia. Although this proves to be a caveat in our work, others have nonetheless shown that changes in any of these microglial properties have been associated with behavioral impairments reminiscent of those observed in neurodevelopmental disorders (*11*, *13*, *14*). The reliance on the development of these properties to changes in neuronal activity ensures that microglia will grow with the changing demands of the nascent cortical circuits. For instance, in mouse models of epilepsy and humans with temporal epilepsy, increased microglia densities have been reported in the affected regions (*67*). As microglia in the adult brain have been shown to modulate neuronal activity negatively (*63*), changes in microglial numbers are likely to be a precautionary measure to ensure that the circuits do not remain hyperactive. Consequently, in mouse models where microglia have been removed either pharmacologically or genetically, these mice have been associated with worsening seizures and severity outcomes (*68*).

Notably, cortical microglia behave similarly to other cortical cell types in the cortex, such as interneurons (*28*, *34*, *69*), astrocytes (*70*, *71*), oligodendrocytes (*39*), and endothelial cells (*38*). The numbers, morphologies, and gene expression profiles of these cells are modulated by neuronal activity. The ability of these cells to sense and adapt to the changes in neuronal activity highlights a potential common mechanism as to how cortical cellular ratios are established during development and potentially how they are maintained during evolution. The dependency of these cortical ratios on changes in neuronal activity could also be a weakness during brain development. Changes in levels, patterns of spontaneous activities, and functional connectivity are common threads observed in most neurodevelopmental disorders (*72*–*75*). Consequently, in addition to the cell autonomous modifications that genetic mutations may induce, there is also a secondary noncell autonomous set of changes induced by altered neuronal activity that can further contribute to the behavior impairments typically observed in neurodevelopmental disorders. Understanding how these primary and secondary impairments may contribute to neurodevelopmental disorders will be vital in understanding how dysregulation of brain development may contribute to neurodevelopmental disorders.

## MATERIALS AND METHODS

### Mice

The mouse lines *Nex^Cre/+^* (*Neurod6^tm1(cre)Kan^*) (*76*), Fucci2 mice (*RCL^Fucci2aR/+,^* provided by R. L. Mort) (*77*), *Cx3Cr1^Cre/+^* (JAX: 025524) (*78*), *Acvr1b*^*f/f*^ (JAX: 036934) (*79*), and CD1 (Envigo) were used in this study. All experiments were performed following the guidelines of the University of Manchester Biological Services Facility and in accordance and approval with the European regulations and Home Office personal and project licenses under the UK animals (Scientific Procedures 1986 Act). Unless specified, both male and female mice were used indiscriminately throughout this study. To generate *Nex^Cre/+^;Fucci2* mice, *Nex^Cre/Cre^* mice were crossed with Fucci2 mice. To generate *Cx3Cr1^Cre/+^;Acvr1b^f/f^* mice, *Acvr1b^f/f^* mice were crossed with *Cx3Cr1^Cre/+^;Acvr1b^f/+^* animals. Animals were housed in groups of up to five littermates and maintained under standard, temperature-controlled, laboratory conditions. Unless otherwise stated, mice were kept on a 12-hour/12-hour light/dark cycle and received water and food ad libitum. For dark-rearing experiments, mice are kept in ventilated completely light-tight cabinets from postnatal day 1 and up to 3 days after eye opening and received water and food ad libitum with specified light conditions.

### Whisker plucking

P10 pups were anesthetized using isoflurane. All whiskers were plucked from the right side of the snout by applying a small tension to the base of the vibrissae with care to prevent damage to the whisker follicle. The pups were then placed in a warmed recovery chamber and returned to their mothers. The plucked whiskers are checked daily to ensure no regrowth and replucked if necessary, until P12.

### Behavior procedures

We used 2- to 4-month-old mice for the behavioral experiments. Behavior analyses were performed by as a test battery in the following order: open field, light-dark box, novel object recognition, and social preference tests. Before the start of the test battery, mice were handled for three consecutive days by the same experimenter. On each day of the test, mice were moved to the testing room at least 30 min prior to the onset of behavioral testing. All testing took place between 9:00 a.m. to 2:00 p.m., the first half of the light phase.

### Open field

For the open field test, mice were placed in the center of a black Plexiglas arena (43 cm by 43 cm by 43 cm) under uniform dim red light (<50 lux). Mice were placed individually onto the center of the arena and were allowed to explore for 10 min. Free movement of the mice was analyzed by the tracking software ANY-maze (version 7.4, Stoelting Europe). Rearing and jumping incidences, however, were blindly recorded manually.

### Light-dark test

For the light-dark box test, the apparatus consists of light and dark chambers adhered to one another. Mice can move freely from one chamber to the other through a small opening in the center of the dark chamber. The illumination of the light chamber was 100 to 200 lux, whereas the dark chamber was completely blocked from light. Mice were first introduced into the light chamber, and the free movement between two chambers was recorded for 10 min via the tracking software ANY-maze (version 7.4, Stoelting Europe). However, if there was any discrepancy observed in the tracking, mice were then manually scored blindly through the recorded video.

### Novel object recognition task

For the novel object recognition, mice were placed individually in a Plexiglas arena (43 cm by 43 cm by 43 cm), similar to the open field arena, for 10 min, and exploration was tracked by the videotracking software ANY-maze (version 7.4, Stoelting Europe) for habituation. On the following day, mice were subjected to object habituation sessions in which two objects identical in shape, color, and odor (Duplo blocks and Pokémon figures) were introduced into the arena for 10 min. Objects were placed at least 5 cm away from the walls. The mouse was then returned to their home cages between sessions. One hour after the habituation session, one of the objects was replaced with a novel object. The mouse is then left in the arena for object exploration for 6 min. Time spent in active exploration of each object (defined by distance of snouts to object less than 2 cm) was scored during each session manually by a blinded observer from the video recording. Climbing onto the object (unless the mouse sniffed the object it had climbed on) did not qualify as active exploration. The arena and objects were cleaned with 70% ethanol between each session. To obtain the discrimination index, the difference between the time spent exploring the novel object (*T*_novel_) from the familiar object (*T*_familiar_) (*T*_novel_ − *T*_familiar_) is divided by the total time exploring the objects (*T*_novel_ + *T*_familiar_). Mice were excluded from the experiment if the time spent exploring the familiar object during habituation is less than 20 s.

### Social preference task

For the social preference task, the apparatus has three sequential chambers where each chamber was connected by a removable gate. During habituation, the mouse was placed into the center chamber and was allowed to move the whole apparatus for 10 min. After habituation, the mouse was placed onto a holding cage for 2 min. During this time, a novel mouse (approximately the same age and sex) and inanimate object were placed within a perforated plexiglass tube at the left and right side of the chambers. The time spent investigating each stimulus (e.g., sniffing the stimulus but not climbing on the Plexiglas tube) and the time spent in each chamber were measured manually by a blinded experimenter. To obtain the social preference discrimination index, the difference between the time spent exploring the mice (*T*_mice_) from the inanimate object (*T*_object_) (*T*_mice_ − *T*_object_) is divided by the total time spent exploring (*T*_mice_ + *T*_object_). Mice were excluded from the experiment if the total time spent exploring is less than 20 s.

### Intracranial injections

*pAAV8-hSyn-DiO-hM3D(Gq)-mCherry* were gifts from B. Roth (Addgene plasmid no. 44361) (*80*). P0 and P1 mice were anesthetized with isoflurane and mounted in a stereotaxic frame. For examining changes of neuronal activity on microglia, pups were injected with 600 nl of *pAAV8-hSyn-DiO-hM3D(Gq)-mCherry*. For investigating the role of Activin A signaling on microglia, pups were injected with 600 nl of *pAAV8-hSyn-DiO-hM3D(Gq)-mCherry* with either *pAAV8-EF1a-DIO-shLacZ-eGFP* or *pAAV8-EF1a-DIO-shInhba-EGFP* (BrainVTA). Injections were targeted for the somatosensory or visual cortices with an injection rate of 10 nl/s.

### Drugs

For DREADDs experiments, CNO (Tocris) was dissolved in 5% dimethyl sulfoxide (DMSO; Sigma-Aldrich) and then diluted with 0.9% saline to 1 mg/ml. Pups were injected with vehicle (0.05% DMSO) or CNO (1 g/10 ml) subcutaneously twice daily. For microglia depletion studies, PLX5622 (MedChemExpress) was dissolved in DMSO (Sigma-Aldrich) to 50 mg/ml. PLX5622 (50 mg/kg) was administered subcutaneously once daily.

### Histology

Mice were anesthetized with an overdose of sodium pentobarbital and transcardially perfused with saline followed by 4% paraformaldehyde. Brains were postfixed for 2 hours at 4°C. Brains were sectioned on a vibratome at 60 μm. All primary and secondary antibodies were diluted in phosphate-buffered saline (PBS) blocking solution containing 0.25% Triton X-100 and 2% bovine serum albumin. Sections were incubated overnight at 4°C with primary antibodies in blocking solution. Sections were washed three times with 1x PBS and then incubated with secondary antibodies diluted in blocking solution. Sections were then washed three times with 1x PBS and mounted with Mowiol-Dabco.

The following primary antibodies were used: goat anti-Activin A (1:100, R&D Systems, catalog no. AF338), mouse anti-Bassoon (1:500, Abcam, catalog no. AB82958), rabbit anti-Homer (1:500, Synaptic Systems, catalog no. 160 002), chicken anti-GFP (green fluorescent protein) (1:3000, Aves Lab, catalog no. GFP-1020), goat anti-Iba1 (1:500, Biotechne, catalog no. NB100-1028), guinea pig anti-Iba1 (1:500, Synaptic Systems, catalog no. 234 004), rat anti-RFP (red fluorescent protein) (1:500, Chromotek, catalog no. 5f8-100), rabbit anti-Ki67 (1:500, Abcam, catalog no. ab16667), rat anti-P2RY12 (1:100, BioLegend, catalog no. 848002), rabbit anti-pSMAD3 (1:200, Abcam, catalog no. ab52903), goat anti-Sox9 (1:200, Biotechne, catalog no. AF3075), and rabbit anti-Acvr1b (1:100, Thermo Fisher Scientific, catalog no. Ma5-38039).

### Activin A measurement

The visual cortex was dissected bilaterally in ice-cold PBS. The tissue was then homogenized, and the levels of Activin A in the mouse visual cortex were measured using the Activin A Quantikine colorimetric sandwich ELISA following the manufacturer’s instruction (R&D Systems). All brain homogenates were run in duplicate.

### Image acquisition and image analysis

Images used for analysis were obtained on the SP8 confocal microscope (Leica) using the LAS AF software. Samples from the same experimental litter were imaged and analyzed in parallel, using similar laser powers, photomultiplied gain, and detection filter settings. Cortical layers were identified based on their distinct histological characteristic. L1 was identified as a sparsely populated cell layer. The border between L2/3 and L4 was distinguished by the higher density of L4. L5 was identified as the layer basal to L4 and above L6, which contains less densely packed nuclei.

For analysis of cell density, tissue samples were imaged using a 10x objective, 0.4 numerical aperture (NA), and 2048 by 2048 pixels.

For analysis of synaptic density, tissue samples were imaged using a 40x oil immersion objective, 1.4 NA, and 1024 by 1024 pixels.

For analysis of microglia reconstruction, tissue samples (L2/3 of somatosensory and visual cortex) were imaged using a 40x objective, 1.4 NA, and 1024 by 1024 pixels with a step size of 0.2 μm to produce confocal stacks of ~10 to 15 μm.

### Computational pipeline to identify protein-protein interactions

A list of genes expressed by microglia at P14 was downloaded (*37*). Only genes expressed with a value of 5 fragments per kilobase of transcript per million mapped reads (FPKM) have been considered. To identify proteins localized to the membrane, a list of membrane proteins was identified using the Human Protein Atlas (https://proteinatlas.org).

A list of mouse and human ligand-receptor pairs was downloaded from the CellTalkDB (*46*) (https://github.com/ZJUFanLab/CellTalkDB) and CellPhoneDB (*47*) (https://github.com/ventolab/CellphoneDB). The corresponding ligand-receptor pairs are then filtered and identified.

To determine the activity-dependent ligand expression in pyramidal cells, we accessed the data published by Hrvatin *et al.* (*45*) (https://greenberg.hms.harvard.edu/gene-database/).

Last, to ensure that the ligands are expressed by pyramidal cells at P12, we downloaded the transcriptomic postnatal data of pyramidal cells from GEO DataSets, accession number GSE120161 (*43*).

### Quantification and statistical analysis

All of the statistical details of experiments can be found in the figure legend and in table S1 where all statistical test used, mean, SEM, exact value of *n*, and what *n* represents can be found. In all experiments, all data were obtained from at least two independent sets of experiment.

Unless otherwise stated, all results were plotted and tested for significance using Prism 10. The samples were tested for normality using the Shapiro-Wilk normality test. Paired comparisons were analyzed using two-tailed unpaired Student’s *t* test (normally distributed) and Mann-Whitney test (for not normally distributed). Multiple comparisons with a single variable were analyzed using one-way analysis of variance (ANOVA) with Dunnett’s post hoc test (comparing the mean of each column with the mean of a control column). Statistical significance was considered at *P* values ≤ 0.05.

Because of the small soma size and highly ramified nature of microglia, microglia cell density within cortical layers was quantified manually in a rectangular area, 551.5 μm wide at the pia surface. Cells were counted without using pseudocolor.

For analysis of microglia to pyramidal cell ratios in different cortical areas, cells were quantified using the Imaris 8 built-in spot detection algorithm. Microglia were identified as “spots” of 7 diameter, whereas pyramidal cell nuclei were identified as “spots” of 5 diameter.

To measure immunofluorescence intensity for activin and P2RY12, self-designed Cell Profiler (https://cellprofiler.org) pipelines were used. In brief, for P2RY12 fluorescence intensity, Iba1+ microglia in L2/3 were identified as primary objects using the adaptive minimum cross-entropy thresholding method. Any objects that are beyond the preset diameter range (40 to 10000 pixels) were excluded. P2RY12 fluorescence intensity was measured under this cell mask. For activin, nuclei positive for 4′,6-diamidino-2-phenylindole (DAPI) staining were identified as primary objects using the adaptive minimum cross-entropy thresholding method, and any objects that is beyond the preset diameter range (25 to 100 pixels) were excluded. A distance of 10 to 20 pixels away from the identified nuclei was identified as secondary objects. To measure the immunofluorescence intensity, a tertiary object was created where any overlap between the secondary object and the primary object was removed. Activin A intensity was measured under this subtracted cell mask.

For analysis of excitatory synapse analysis, single planes of L2/3 were analyzed using the Synapse Counter plugin for ImageJ (https://github.com/SynPuCo/SynapseCounter).

For analysis of microglia engulfment, confocal stacks were analyzed with the IMARIS software. All channels were subjected to background subtraction and Gaussian filtering. The 3D isosurfaces were created for Iba1+ microglia, and volume was quantified automatically. The postsynaptic Bassoon+ boutons were reconstructed as “spots” of 0.5 diameter. Using a built-in spot detection algorithm in Imaris and through manual inspections, spots that were located within the isosurface would be considered as engulfed synapses.

Scholl analyses were performed with SNT plugin in Fiji (ImageJ) (*81*).
