# Supplementary material for: Activity-dependent regulation of microglia numbers by pyramidal cells during development shape cortical functions
Source: Sci Adv. 2025 Feb 19;11(8):eadq5842. doi: 10.1126/sciadv.adq5842 (PMC11838000; doi:10.1126/sciadv.adq5842)
Supplement: Supplementary file 1 — Figs. S1 to S18 Tables S1 and S2 [file sciadv.adq5842_sm.pdf]

Science Advances
AAAS

Supplementary Materials for

Activity-dependent regulation of microglia numbers by pyramidal cells during development shape cortical functions

Sanjana Kumaraguru *et al.*

Corresponding author: Fong Kuan Wong, fongkuan.wong@manchester.ac.uk

Sci. Adv. **11**, eadq5842 (2025)
DOI: 10.1126/sciadv.adq5842

This PDF file includes:

Figs. S1 to S18
Tables S1 and S2

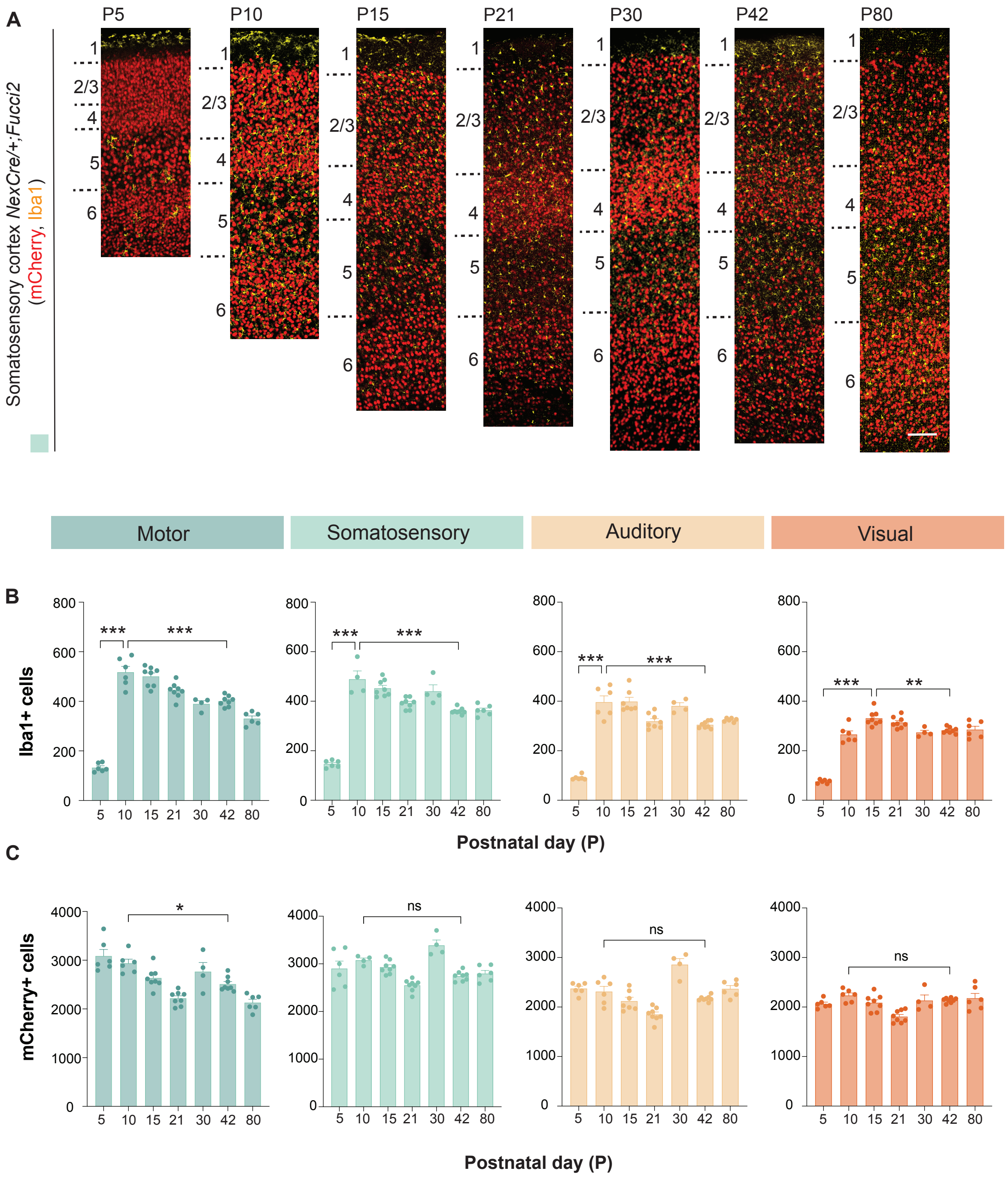

A
P5
P10
P15
P21
P30
P42
P80
1
2/3
4
5
6
Somatosensory cortex NexCre/+;Fucci2
(mCherry, Iba1)
Motor
Somatosensory
Auditory
Visual
B
Iba1+ cells
***
**
Postnatal day (P)
C
mCherry+ cells
*
ns
Postnatal day (P)

Figure S1. Sequential microglia developmental trajectories across cortical areas

(**A**) Coronal sections through the primary somatosensory cortex of $Nex^{Cre/+}$*;Fucci2* mice at different developmental stages following immunohistochemistry against mCherry (red), and Iba1 (yellow). (**B**, **C**) Total number of microglia (Iba1+) (**B**) and pyramidal cells (**C**) at different developmental stages in the motor (dark green), somatosensory (light green), auditory (yellow) and visual cortices (orange). (One-Way ANOVA with Tukey multiple comparison, Microglia: Motor: F=93.06, ***p <0.0001 (P5 *vs* P10 and P10 *vs* P42), Somatosensory: F = 71.91, ***p <0.0001 (P5 vs P10 and P10 vs P42), Auditory: F = 62.60, ***p <0.0001 (P5 *vs* P10) and ***p = 0.0002 (P10 *vs* P42), and Visual: F = 76.57, ***p <0.0001 (P5 *vs* P15) and **p = 0.0047 (P15 *vs* P42). Pyramidal cells: Motor: F=16.17, *p = 0.0151 (P10 *vs* P42), Somatosensory: F = 10.30, Auditory: F = 18.33, and Visual: F = 6.332. n = 4-8 mice across different developmental stages). For bar graphs, data points indicate the average cell density in each animal. Scale bar, 100 µm.

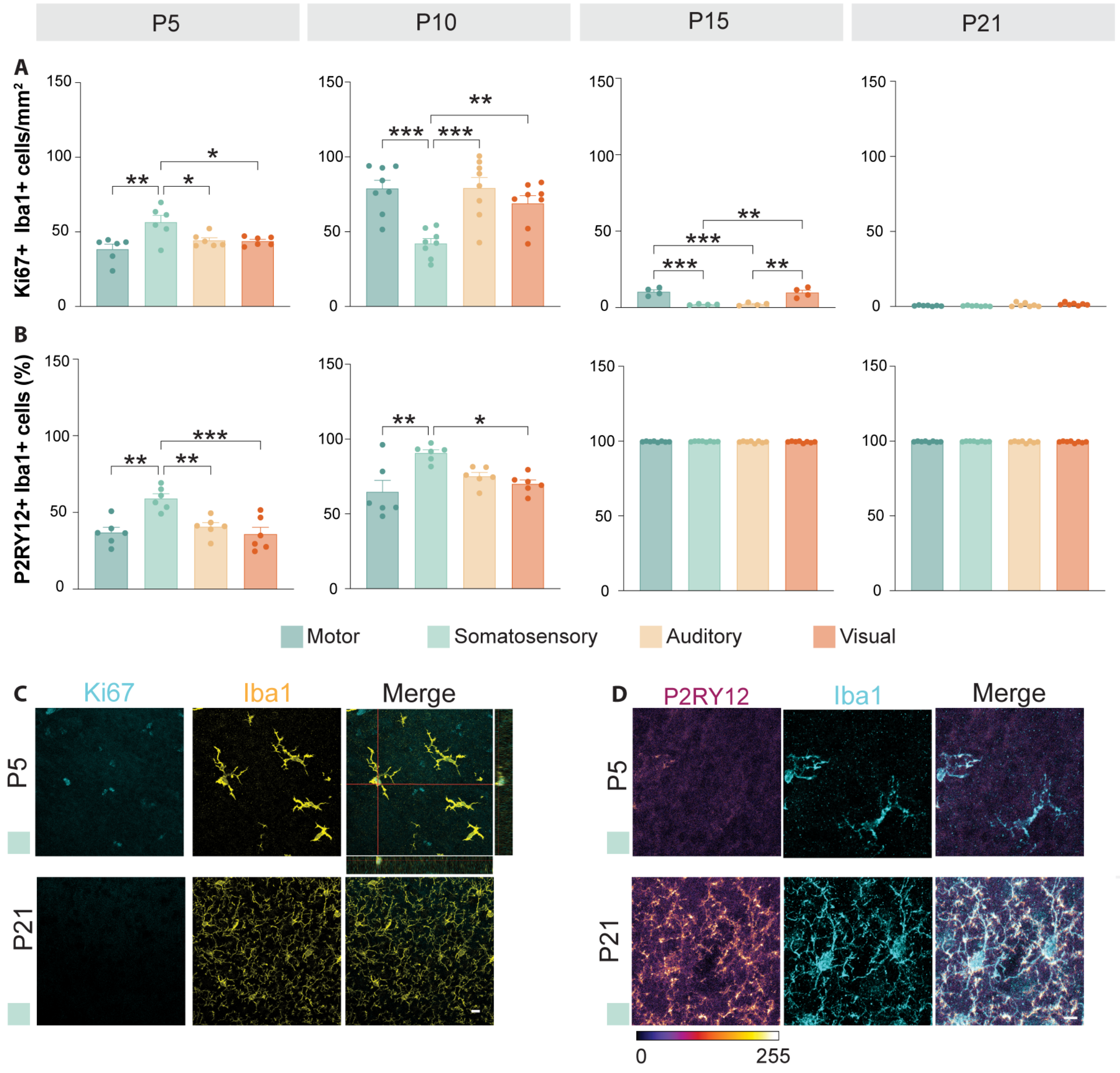
P5
P10
P15
P21
A
Ki67+ Iba1+ cells/mm²
150
100
50
0
B
P2RY12+ Iba1+ cells (%)
Motor
Somatosensory
Auditory
Visual
C
Ki67
Iba1
Merge
D
P2RY12
Iba1
Merge
0
255

Figure S2. Sequential microglia developmental trajectories across cortical areas impact on proliferation and maturation

(**A**, **B**) Quantification of Ki67+ Iba1+ cells (**A**) and P2RY12+ Iba1+ (**B**) at different developmental stages in the motor (dark green), somatosensory (green), auditory (yellow), and visual cortices (orange). One-way ANOVA with Tukey multiple comparison (Ki67: P5: F = 6.35, **p = 0.002 (motor *vs* somatosensory), *p = 0.045 (somatosensory *vs* auditory) and *p = 0.035 (somatosensory *vs* visual), P10: F = 10.35, ***p = 0.0003 (motor *vs* somatosensory), ***p = 0.0002 (somatosensory *vs* auditory) and **p=0.0081 (somatosensory *vs* visual), P15: F = 18.64, , ***p = 0.0007 (motor *vs* somatosensory), ***p = 0.0008 (motor *vs* auditory), **p = 0.0012 (somatosensory *vs* visual) and **p = 0.0014 (auditory *vs* visual), P21: F = 3.04), P2RY12: P5: F = 9.85, **p = 0.001 (motor *vs* somatosensory), **p = 0.006 (somatosensory *vs* auditory) and ***p = 0.0006 (somatosensory *vs* visual), P10: F = 6.51, **p = 0.0024 (motor *vs* somatosensory) and *p = 0.016 (somatosensory *vs* visual), P15: F = 2.01, P21: F = 0.93), n = 4-8 mice across different developmental stages). (**C**, **D**) Coronal sections through the primary somatosensory cortex of *Nex*$^{Cre/+}$*;Fucci2* mice at P5 (top) and P21 (bottom) following immunohistochemistry against (**C**) Ki67 (cyan), and Iba1 (yellow) and (**D**) P2RY12 (fire lookup table) and Iba1 (cyan) . Orthogonal z-stack view of Ki67+ and Iba1+ is illustrated for selected cells in P5. Scale bars: 10 µm.

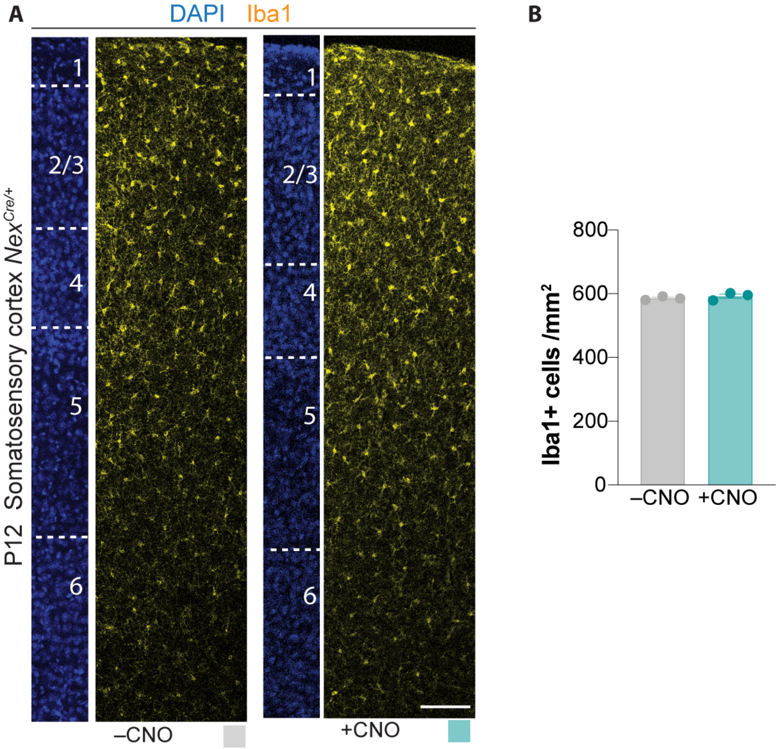
A
DAPI Iba1
P12 Somatosensory cortex Nex^Cre/+
1
2/3
4
5
6
−CNO
+CNO
B
800
600
400
200
0
Iba1+ cells /mm²
−CNO +CNO

Figure S3. CNO injection alone has no impact on microglial numbers

(**A**) Coronal sections through S1BF of *Nex*$^{Cre/+}$ mice at P12 injected with vehicle or CNO treatment immunostained for Iba1 (yellow). DAPI is shown for counterstaining (blue). (**B**) Quantification of Iba1+ cells at P12. Two-tailed Student's unpaired t-test, n = 3. Scale bar: 100 µm

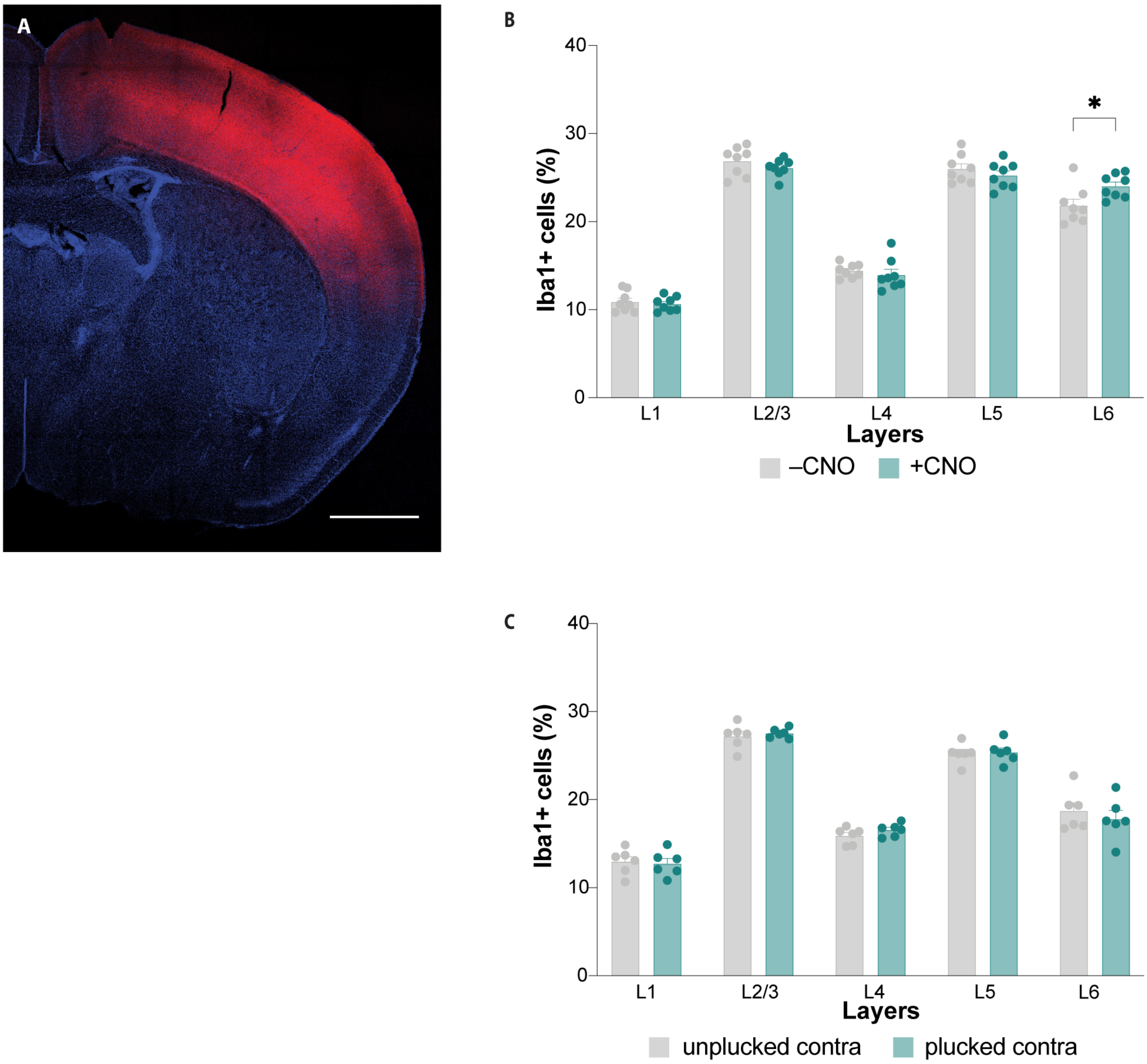
A
B
40
30
20
10
0
Iba1+ cells (%)
*
L1
L2/3
L4
L5
L6
Layers
–CNO
+CNO
C
40
30
20
10
0
Iba1+ cells (%)
L1
L2/3
L4
L5
L6
Layers
unplucked contra
plucked contra

Figure S4: Changes in neuronal activity has minimal impact on microglia distribution

(**A**) Coronal sections through the hemisphere of *Nex*$^{Cre/+}$mice injected with *hM3Dq-mCherry* at P12 immunostained against mCherry (red). DAPI is shown for counterstaining (blue). (**B**) Quantification of the distribution of Iba1+ cells treated with vehicle or CNO (Two-way ANOVA, $F_{treatment} = 2.97 \times 10^{-16}$, *$p$ = 0.014, n = 8). (**C**) Quantification of the distribution of Iba1+ cells of the contralateral hemisphere of CD1with whiskers plucked or unplucked (two-way ANOVA, $F_{treatment} = 0.00$, n = 6). Scale bar: 1000 µm.

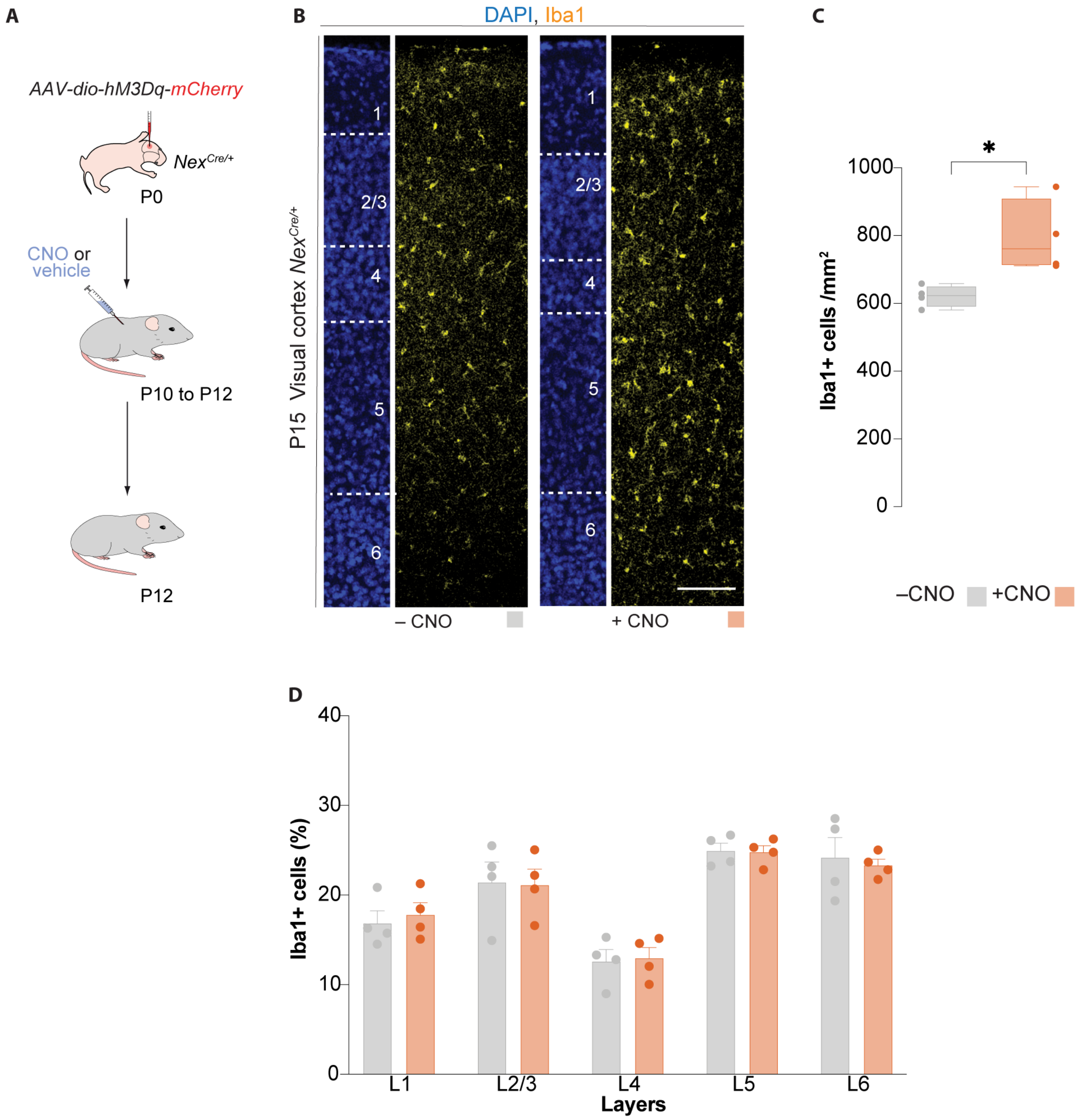

A
AAV-dio-hM3Dq-mCherry
Nex^Cre/+
P0
CNO or vehicle
P10 to P12
P12
B
DAPI, Iba1
P15 Visual cortex Nex^Cre/+
1
2/3
4
5
6
– CNO
+ CNO
C
*
Iba1+ cells /mm²
1000
800
600
400
200
0
–CNO
+CNO
D
Iba1+ cells (%)
40
30
20
10
0
L1
L2/3
L4
L5
L6
Layers

Figure S5. Modulation of pyramidal cell activity regulates microglial numbers in the visual cortex

(**A**) Schematic of experimental design. (**B**) Coronal sections through the primary visual cortex of *Nex*$^{Cre/+}$ mice at P12 injected with *hM3Dq-mCherry* virus followed by vehicle or CNO treatment immunostained for Iba1 (yellow). DAPI is shown for counterstaining (blue). (**C**) Quantification of Iba1+ cells at P12. Two-tailed unpaired Student's t-test, *p = 0.022, n = 4. (**D**) Quantification of the distribution of Iba1+ cells in the visual cortex at P12 (Two-way ANOVA, $F_{treatment} = 0.286 \times 10^{-17}$), n = 4. For box plots, the adjacent data points indicate the average cell density in each animal. Scale bar, 100 µm.

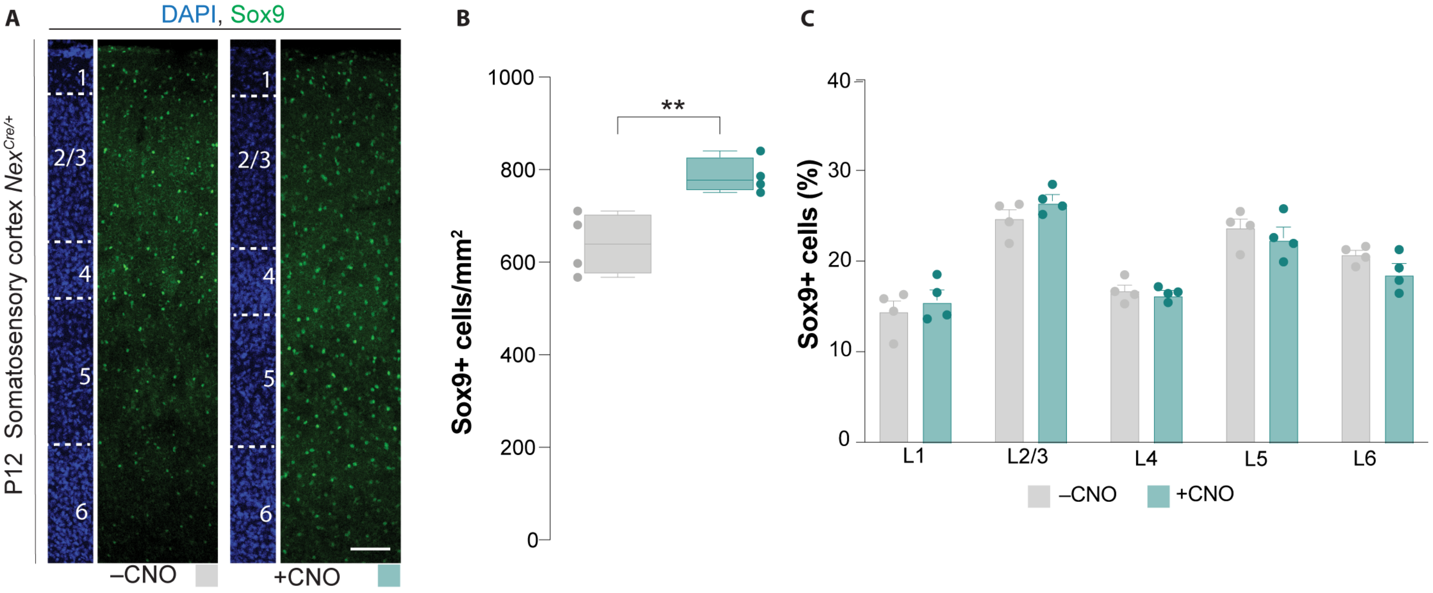
A
DAPI, Sox9
P12 Somatosensory cortex Nex^Cre/+
1
2/3
4
5
6
–CNO
+CNO
B
Sox9+ cells/mm²
1000
800
600
400
200
0
**
C
Sox9+ cells (%)
40
30
20
10
0
L1
L2/3
L4
L5
L6
–CNO
+CNO

Figure S6. Modulation of pyramidal cell activity regulates astrocyte number

(**A**) Coronal sections through the primary somatosensory cortex of *Nex*$^{Cre/+}$ mice at P12 injected with *hM3Dq-mCherry* virus followed by vehicle or CNO treatment immunostained for Sox9 (green). DAPI is shown for counterstaining (blue). (**B**) Quantification of Sox9+ cells in the somatosensory cortex of *Nex*$^{Cre/+}$ mice at P12. 2-tailed unpaired Student's t-test, $**p = 0.0092$, n = 4. (**C**) Quantification of the distribution of Sox9+ cells in the somatosensory cortex at P12. Two-way ANOVA ($F_{treatment} = 0.99$), n = 4. Scale bar, 100 µm.

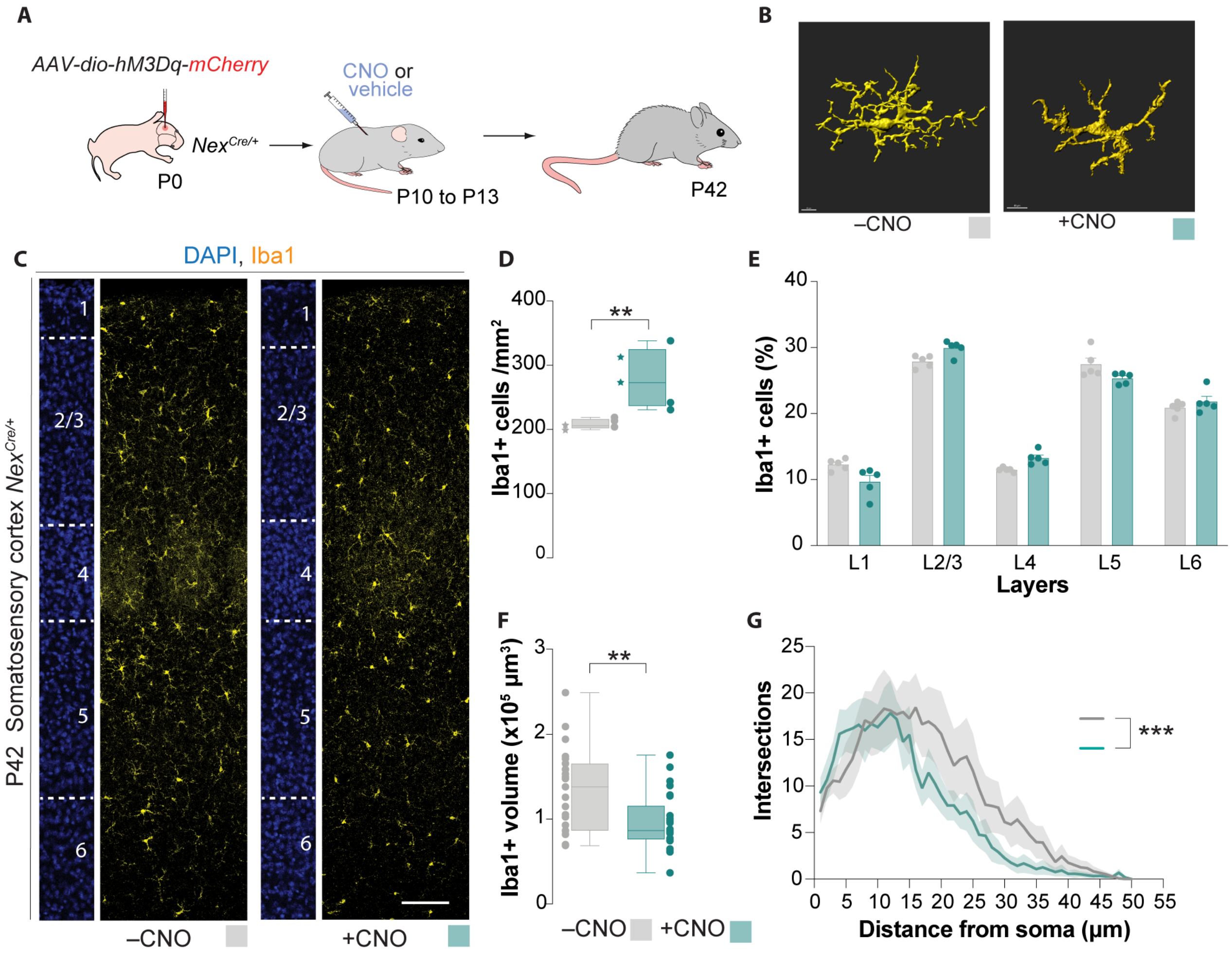
A
AAV-dio-hM3Dq-mCherry
Nex^Cre/+
P0
CNO or vehicle
P10 to P13
P42
B
–CNO
+CNO
C
DAPI, Iba1
P42 Somatosensory cortex Nex^Cre/+
1
2/3
4
5
6
–CNO
+CNO
D
Iba1+ cells /mm²
**
E
Iba1+ cells (%)
L1
L2/3
L4
L5
L6
Layers
F
Iba1+ volume (x10⁵ µm³)
**
–CNO
+CNO
G
Intersections
***
Distance from soma (µm)

Figure S7. Acute modulation of pyramidal cell activity has a long-term impact on microglial numbers

(**A**) Schematic of experimental design. (**B**) 3D reconstruction of microglia. (**C**) Coronal sections through the primary somatosensory cortex of $Nex^{Cre/+}$ mice at P42 injected with *hM3Dq-mCherry* virus followed by vehicle or CNO treatment immunostained for Iba1 (yellow). DAPI is shown for counterstaining (blue). (**D**) Quantification of Iba1+ cells at P42. Mann Whitney, $^{**}p$ = 0.0046, n = 5. (**E**) Quantification of the distribution of Iba1+ cells in the somatosensory cortex of $Nex^{Cre/+}$ mice at P42 (Two-way ANOVA, $F_{treatment}$=1.37x10^{-16}), n = 5. (**F**) Quantification of Iba1+ cell volume at P42 from 5 mice respectively. Two-tailed unpaired Student's t-test, $^{**}p$ = 0.0048. Control: n = 23 cells, CNO: n =25 cells. (**G**) Scholl analysis of microglia 2-way ANOVA (F_{Group} (1, 298) = 18.64, $^{***}p < 0.001$), n = 4. For box plots, the adjacent data points indicate the average cell density in each animal. For (D), star data points (left) indicate male mice while circle data (right) points indicate female mice. Scale bars, 100 µm (B) and 40 µm (C).

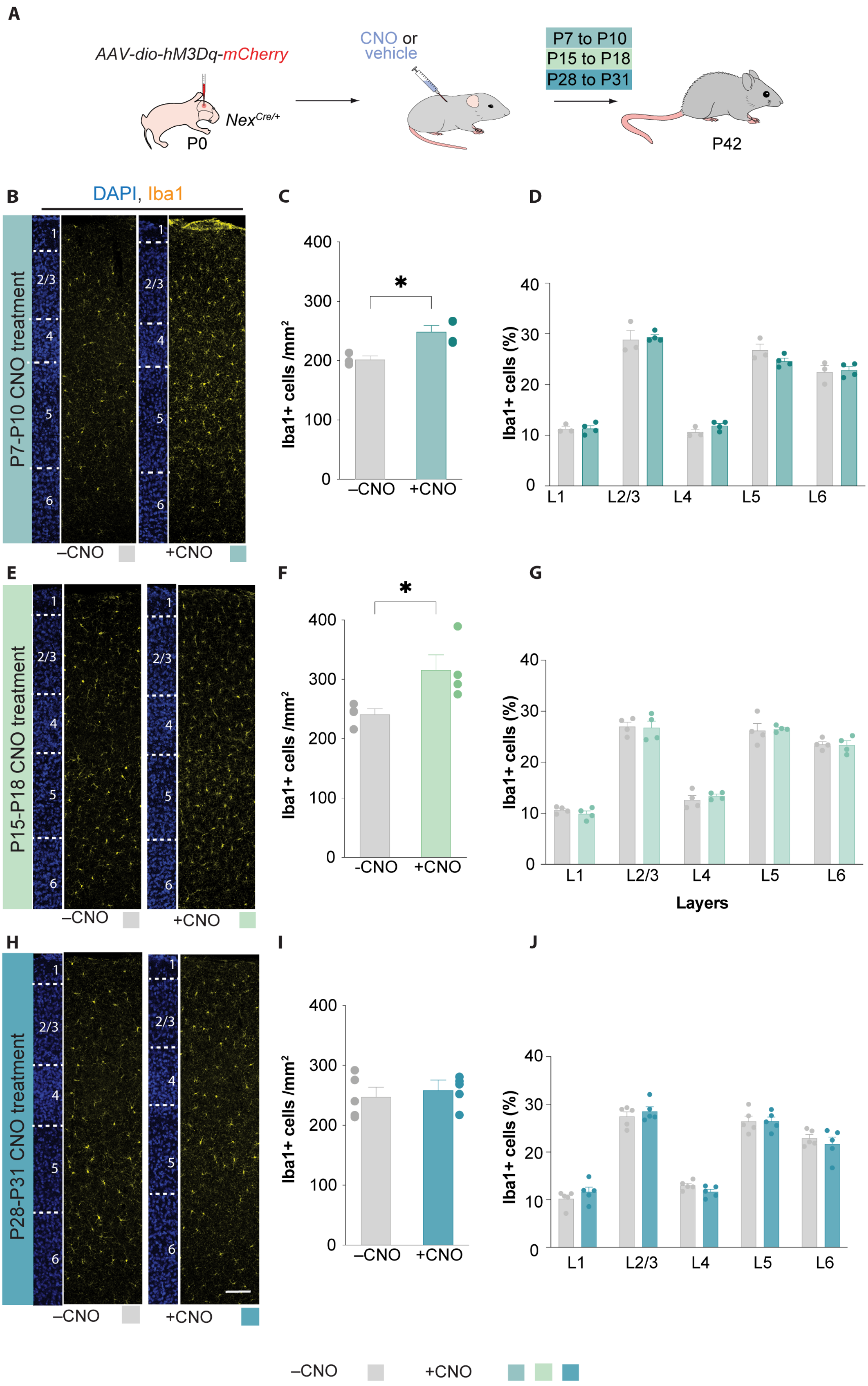
A
AAV-dio-hM3Dq-mCherry
Nex^Cre/+
P0
CNO or vehicle
P7 to P10
P15 to P18
P28 to P31
P42
B
DAPI, Iba1
P7-P10 CNO treatment
–CNO
+CNO
C
Iba1+ cells /mm²
*
D
Iba1+ cells (%)
L1
L2/3
L4
L5
L6
E
P15-P18 CNO treatment
F
G
Layers
H
P28-P31 CNO treatment
I
J

Figure S8. Modulation of pyramidal cell activity regulates microglial numbers during a critical window in development

(**A**) Schematic of experimental design (**B**, **E**, **H**) Coronal sections through the primary somatosensory cortex of *Nex*$^{Cre/+}$ mice at P42 injected with *hM3Dq-mCherry* virus followed by vehicle or CNO treatment between (**B**) P7 to P10, (**E**) P15 to P18 and (**H**) P28 to P31 immunostained for Iba1 (yellow). DAPI is shown for counterstaining (blue). (**C**) Quantification of Iba1+ cells at P42. Two-tailed unpaired Student's t-test, *$p = 0.015$, Control: n = 3, CNO: n = 4. (**D**) Quantification of the distribution of Iba1+ cells at P42, Two-way ANOVA, ($F_{treatment} = 1.45x10^{-17}$), Control: n =3, CNO: n = 4 (**F**) Quantification of Iba1+ cells at P42. Two-tailed unpaired Student's t-test, *$p = 0.033$, n = 4. (**G**) Quantification of distribution of Iba1+ cells at P42, Two-way ANOVA, ($F_{treatment} = 2.40x10^{-16}$), n = 4. (**I**) Quantification of Iba1+ cells at P42. Two-tailed unpaired Student's t-test, n = 5. (**J**) Quantification of distribution of Iba 1+ cells at P42.Two-way ANOVA, ($F_{treatment} = 1.90x10^{-16}$), n = 5. Scale bar, 100 µm.

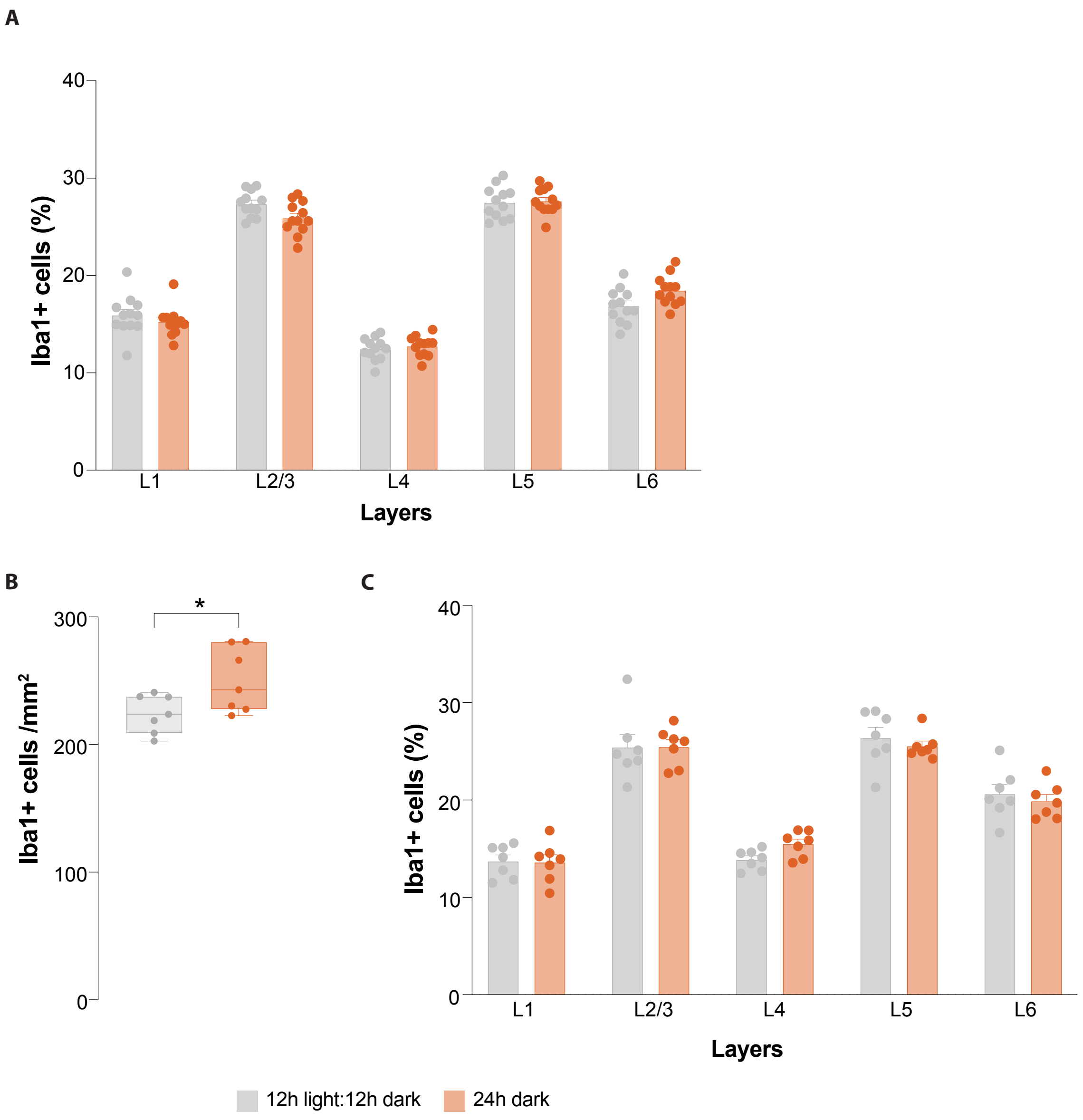
A
40
30
20
10
0
Iba1+ cells (%)
L1
L2/3
L4
L5
L6
Layers
B
300
200
100
0
Iba1+ cells /mm²
*
C
40
30
20
10
0
Iba1+ cells (%)
L1
L2/3
L4
L5
L6
Layers
12h light:12h dark
24h dark

Figure S9. Delayed in changes of spontaneous activity patterns does not alter microglia distribution

(**A**) Quantification of the distribution of Iba1+ cells in the visual cortex at P15. Two-way ANOVA, ($F_{treatment}$ = 9.03x10^{-16}), n = 12. (**B**) Quantification of Iba1+ cells at P80. Two-tailed unpaired Student's t-test, **p* = 0.038, n = 7. (**C**) Quantification of the distribution of Iba1+ cells in the visual cortex at P80. Two-way ANOVA, $F_{treatment}$ =0.00), n = 7.

A

Visual cortex *Nex*$^{Cre/+}$*;Fucci2*
(DAPI, mCherry, ActivinA)

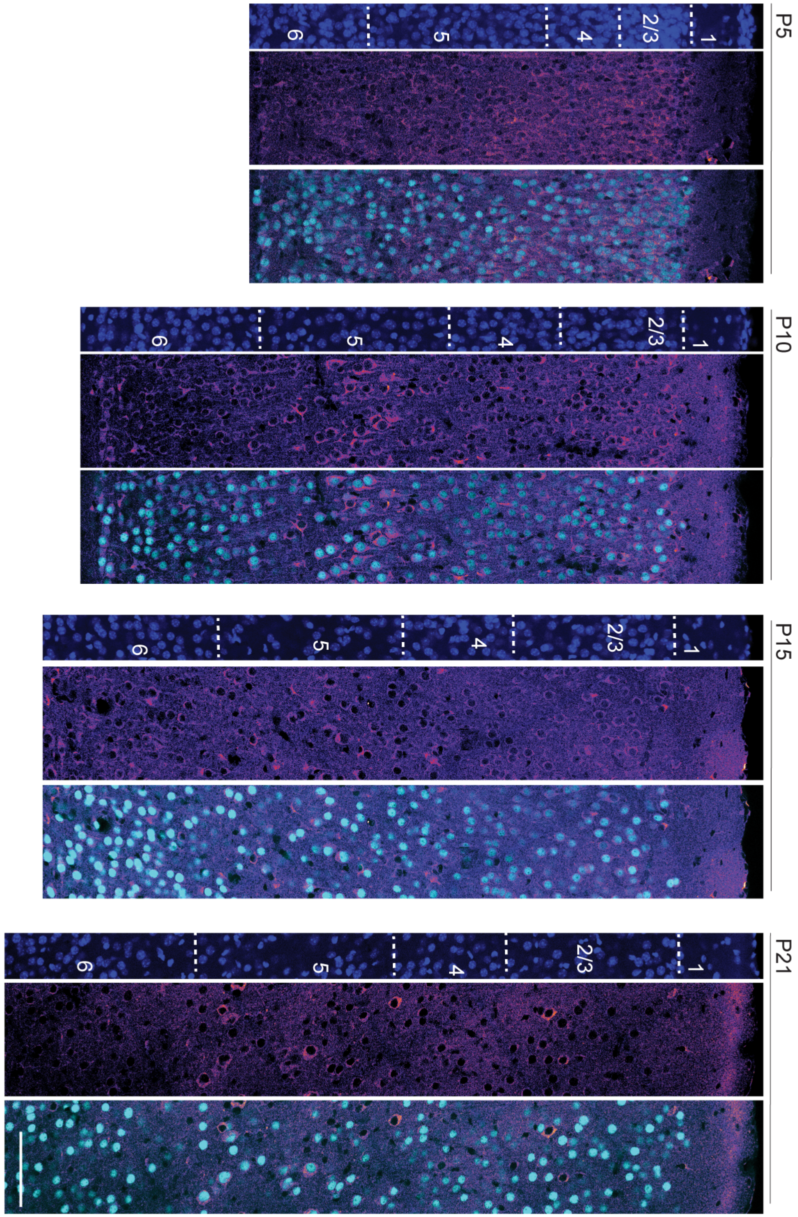

Figure S10. Expression of Activin A during development

(**A**) Coronal sections through the visual cortex of $\textit{Nex}^{Cre/+}$*;Fucci2* mice at different developmental stages following immunohistochemistry against mCherry (cyan), and Activin A (Fire look up table). DAPI is shown for counterstaining (blue). Scale bar: 100 µm.

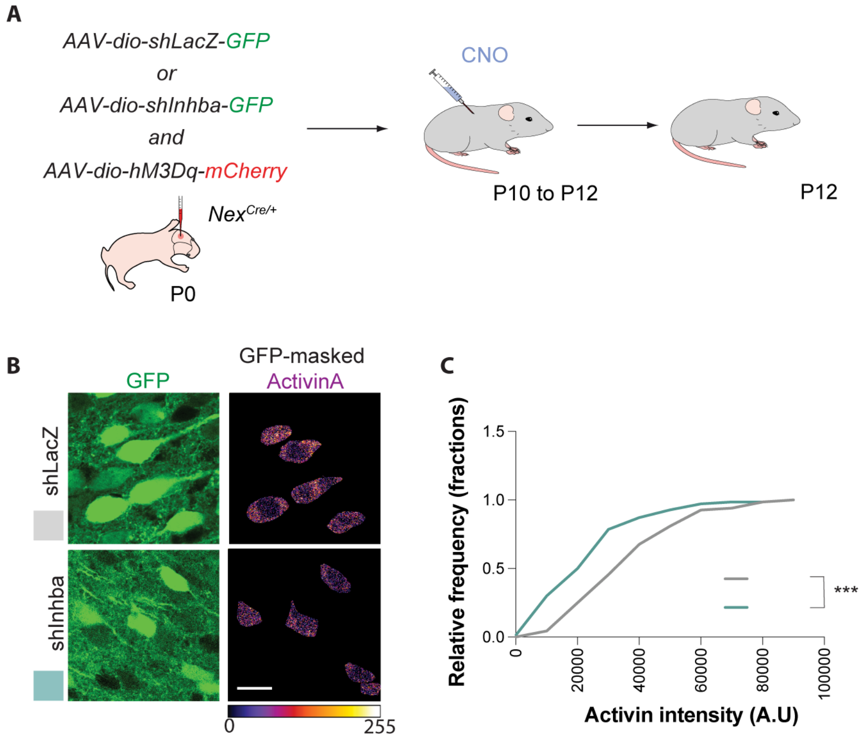

A
AAV-dio-shLacZ-GFP
or
AAV-dio-shInhba-GFP
and
AAV-dio-hM3Dq-mCherry
Nex^Cre/+
P0
CNO
P10 to P12
P12
B
GFP
GFP-masked
ActivinA
shLacZ
shInhba
0
255
C
Relative frequency (fractions)
1.5
1.0
0.5
0.0
0
20000
40000
60000
80000
100000
Activin intensity (A.U)
***

Figure S11. Validation of shRNA virus

(**A**) Schematic of experimental design. (**B**) Coronal sections through L2/3 primary somatosensory cortex of $Nex^{Cre/+}$ mice at P12 immunostained for GFP (green) and Activin A (fire lookup table masked by GFP). (**C**) Cumulative distribution of Activin A intensity in layer 2/3 somatosensory cortex in GFP+ cells in *shLacZ-GFP* (grey) and *shInhba-GFP* virus injected mice (green). Kolmogorov-Smirnov test, ****p*<0.0001; n = 68 cells (*shLacZ*) and 70 cells (*shInhba*) from 4 mice from each group. Scale bar, 10 µm.

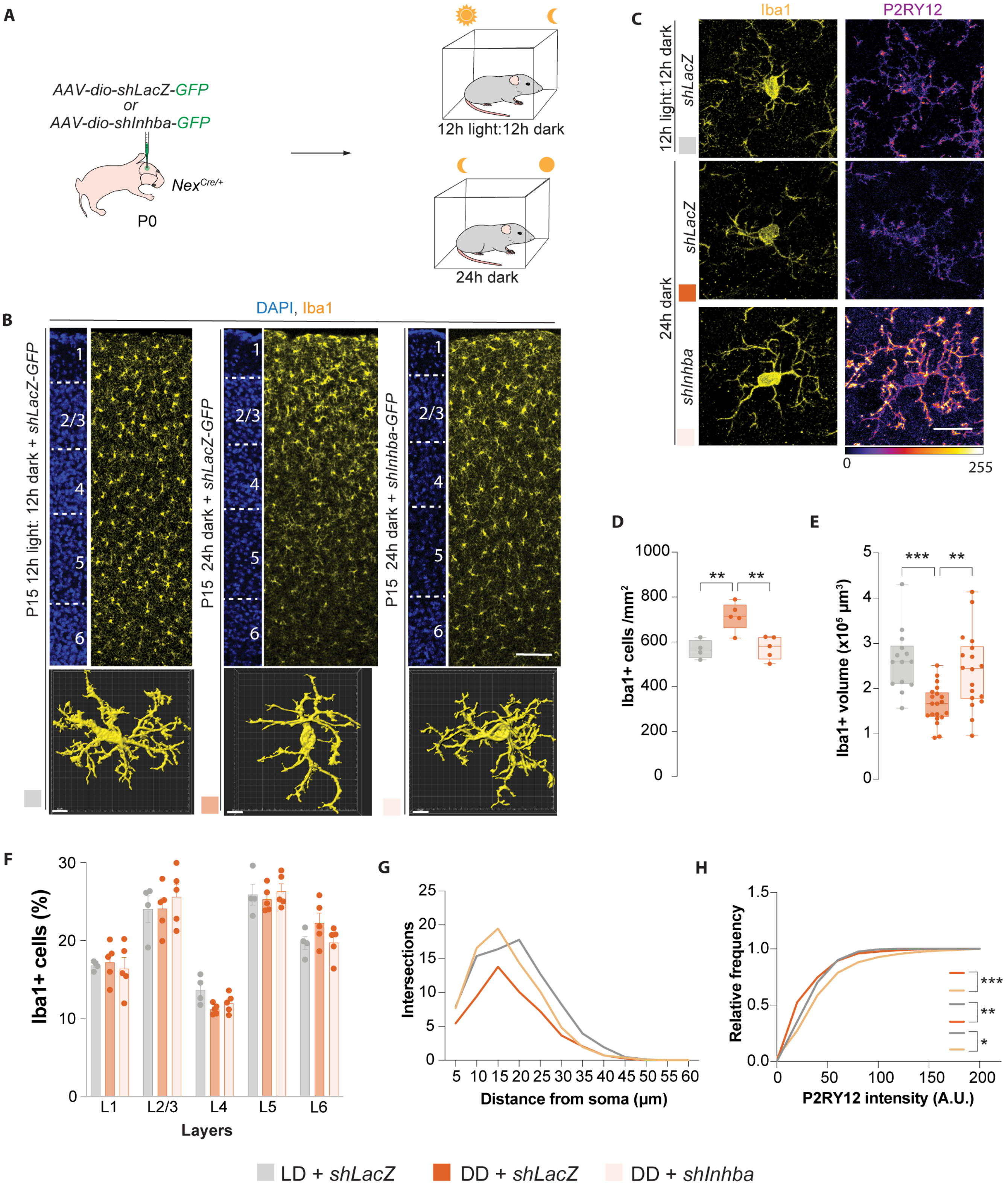

A
AAV-dio-shLacZ-GFP
or
AAV-dio-shInhba-GFP
Nex^Cre/+
P0
12h light:12h dark
24h dark
B
DAPI, Iba1
P15 12h light: 12h dark + shLacZ-GFP
P15 24h dark + shLacZ-GFP
P15 24h dark + shInhba-GFP
1
2/3
4
5
6
C
Iba1
P2RY12
12h light:12h dark
24h dark
shLacZ
shLacZ
shInhba
0
255
D
Iba1+ cells /mm²
E
Iba1+ volume (x10⁵ µm³)
F
Iba1+ cells (%)
L1
L2/3
L4
L5
L6
Layers
G
Intersections
Distance from soma (µm)
H
Relative frequency
P2RY12 intensity (A.U.)
LD + shLacZ
DD + shLacZ
DD + shInhba

Figure S12. Microglia numbers is dependent on activity-dependent Activin A release from pyramidal cells

(**A**) Schematic of experimental design. (**B**, **C**) Coronal sections through primary visual cortex of *Nex*$^{Cre/+}$ mice at P15 injected with either *shLacZ-GFP* or *shInhba-GFP* followed by pups reared in standard light-dark cycle (12h light: 12h dark) or in complete darkness (24h dark) immunostained for (**B**, **C**) Iba1 (yellow) and (**C**) P2RY12 (fire lookup table). DAPI is shown for counterstaining (blue). (**B, bottom**): 3D reconstruction of microglia. (**D**, **F**) Quantification of (**D**) Iba1+ cells and (**F**) distribution of Iba1+ cells. (**D**) One-way ANOVA with Tukey multiple comparisons. (F = 11.35, **p = 0.0049 (*shLacZ-GFP* virus injected pups reared in standard light-dark cycle *vs shLacZ-GFP* virus injected pups reared in complete darkness) and **p = 0.0045 (*shLacZ-GFP* virus *vs shInhba-GFP* injected pups reared in complete darkness), (**F**) Two-way ANOVA. ($F_{treatment} = 2.82x10^{-16}$), shLacZ-GFP virus injected pups in standard light-dark cycle: n= 4, all others, n = 5). (**E**, **G**, **H**) Quantification of Iba1+ (**E**) cell volume, (**G**) scholl analysis and (**H**) cumulative distribution of P2RY12 intensity in layer 2/3 in the primary visual cortex at P15. (**E**) One-way ANOVA with Tukey multiple comparisons. (F = 10.56, ***p = 0.0004 (*shLacZ-GFP* virus injected pups reared in standard light-dark cycle *vs shLacZ-GFP* virus injected pups reared in complete darkness) and **p = 0.0029 (*shLacZ-GFP* virus *vs shInhba-GFP* injected pups reared in complete darkness), (**G**) Two-way ANOVA with Tukey multiple comparisons. ($F_{group\ (2,96)} = 20.93$, ***$p < 0.0001$). (**H**) Kruskal Wallis test. *p = 0.047, **p = 0.005 and ***p = 0.0001, shLacZ-GFP virus injected pups in standard light-dark cycle: n = 14 cells, shLacZ-GFP in complete darkness, n= 20 cells and shInhba-GFP in complete darkness, n= 18 cells. Scale bars, 10 µm (C), 50µm (B, bottom) and 100µm (B, top).

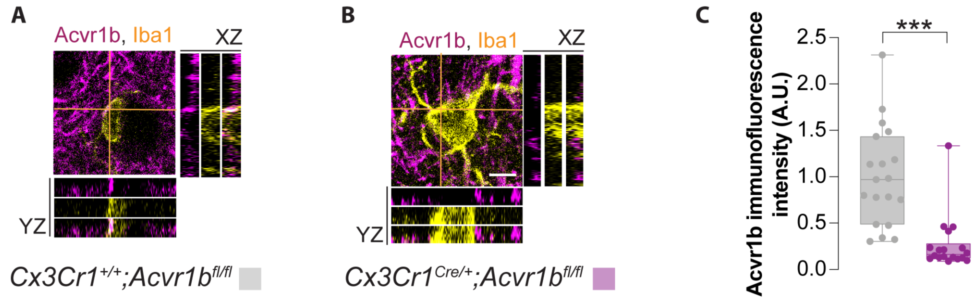
A
Acvr1b, Iba1
XZ
YZ
Cx3Cr1+/+;Acvr1bfl/fl
B
Acvr1b, Iba1
XZ
YZ
Cx3Cr1Cre/+;Acvr1bfl/fl
C
Acvr1b immunofluorescence intensity (A.U.)
***
2.5
2.0
1.5
1.0
0.5
0.0

Figure S13. Validation of *Cx3Cr1*$^{Cre/+}$*;Acvr1b*$^{fl/fl}$ mouse model

(**A**, **B**) Maximum projection intensity of microglia stained with Iba1 (yellow) and Acvr1b (cyan) together with the corresponding orthogonal sections through the stack from (**A**) *Cx3Cr1*$^{+/+}$*;Acvr1b*$^{fl/fl}$ and (**B**) *Cx3Cr1*$^{Cre/+}$*;Acvr1b*$^{fl/fl}$ at P21. (**C**) Immunofluorescence intensity of Acvr1b staining in *Cx3Cr1*$^{+/+}$*;Acvr1b*$^{fl/fl}$ and *Cx3Cr1*$^{Cre/+}$*;Acvr1b*$^{fl/fl}$. Mann-Whitney test, p<0.0001, Control: n = 19 cells, Mutants: n = 18 cells. For box plot, data points indicate the average cell intensity for an individual cell from 4 animals per group. Scale bars, 10 µm.

A

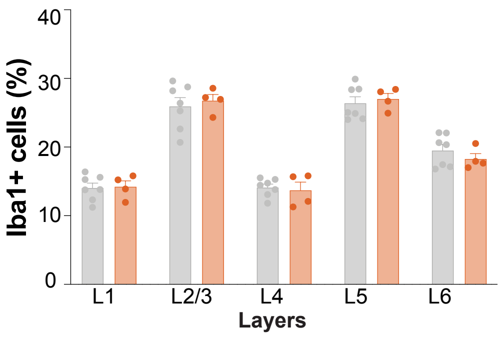

Cx3Cr1$^{+/+}$;*Acvr1b*$^{fl/fl}$ *Cx3Cr1*$^{Cre/+}$;*Acvr1b*$^{fl/fl}$

Figure S14. Altered Activin A signaling in macrophages does not alter microglial distribution in the visual cortex

(**A**) Quantification of distribution of Iba1+ cells in the primary visual cortex in control and *Cx3Cr1*$^{Cre/+}$;*Acvr1b*$^{f/f}$ mice at P21. Two-way ANOVA. ($F_{treatment} = 4.42 \times 10^{-16}$), Control: n = 7, Mutant: n = 4.

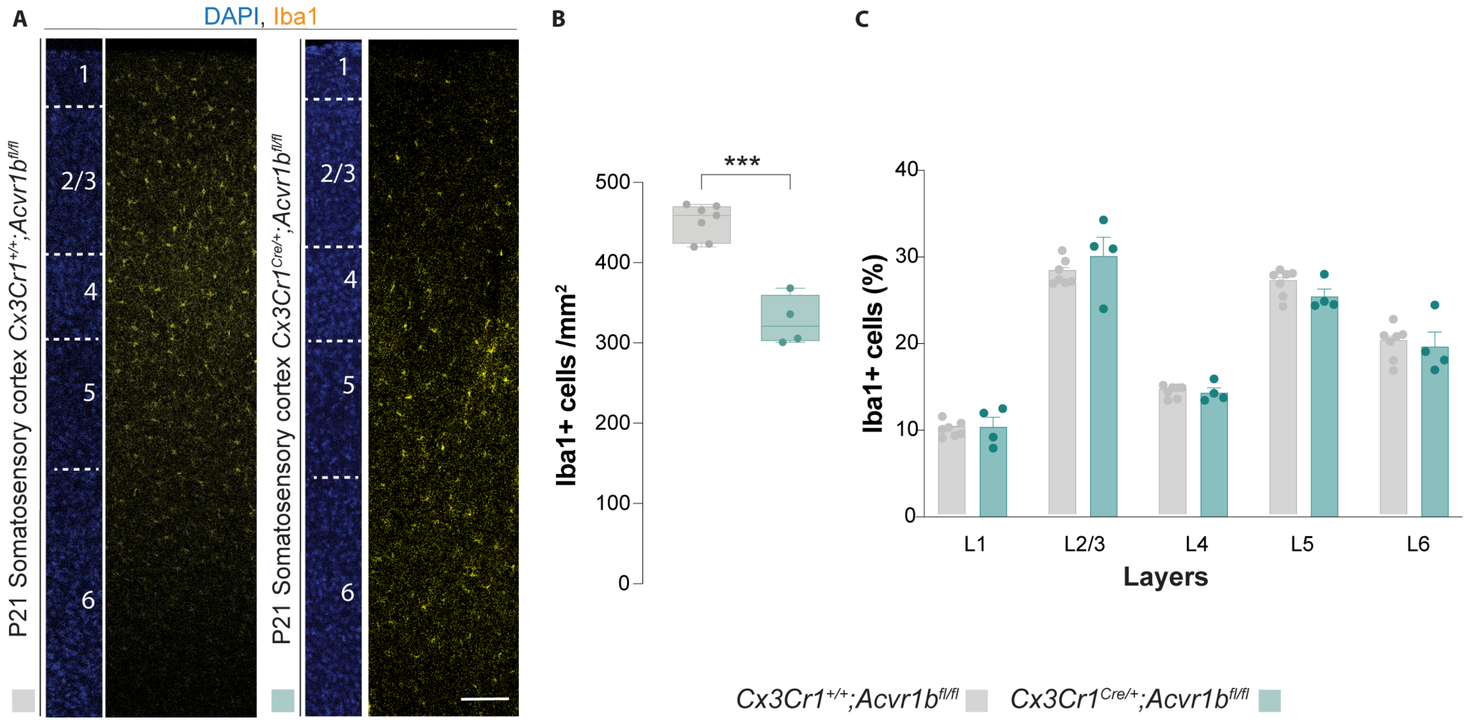
A
DAPI, Iba1
P21 Somatosensory cortex Cx3Cr1+/+;Acvr1bfl/fl
P21 Somatosensory cortex Cx3Cr1Cre/+;Acvr1bfl/fl
1
2/3
4
5
6
B
***
Iba1+ cells /mm2
500
400
300
200
100
0
C
Iba1+ cells (%)
40
30
20
10
0
L1
L2/3
L4
L5
L6
Layers
Cx3Cr1+/+;Acvr1bfl/fl
Cx3Cr1Cre/+;Acvr1bfl/fl

Figure S15. Altered Activin A signalling in macrophages induces changes in microglial numbers in the somatosensory cortex

(**A**) Coronal sections through the S1BF of *Cx3Cr1*$^{+/+}$*;Acvr1b*$^{f/f}$ and *Cx3Cr1*$^{Cre/+}$*;Acvrb*$^{f/f}$ mice at P21 following immunohistochemistry for Iba1 (yellow). DAPI is shown for counterstaining (blue). (**B**) Quantification of Iba1+ cells in the S1BF at P21. 2-tailed unpaired Student's t-test, ****p* = 0.0007, Control: n = 7, Mutant: n = 4. (**C**) Quantification of distribution of Iba1+ cells at P21. Two-way ANOVA. ($F_{treatment} = 3.37x10^{-17}$), Control: n = 7, Mutant: n = 4. For box plot, data points indicate the average cell density in each animal. Scale bar, 100 μm.

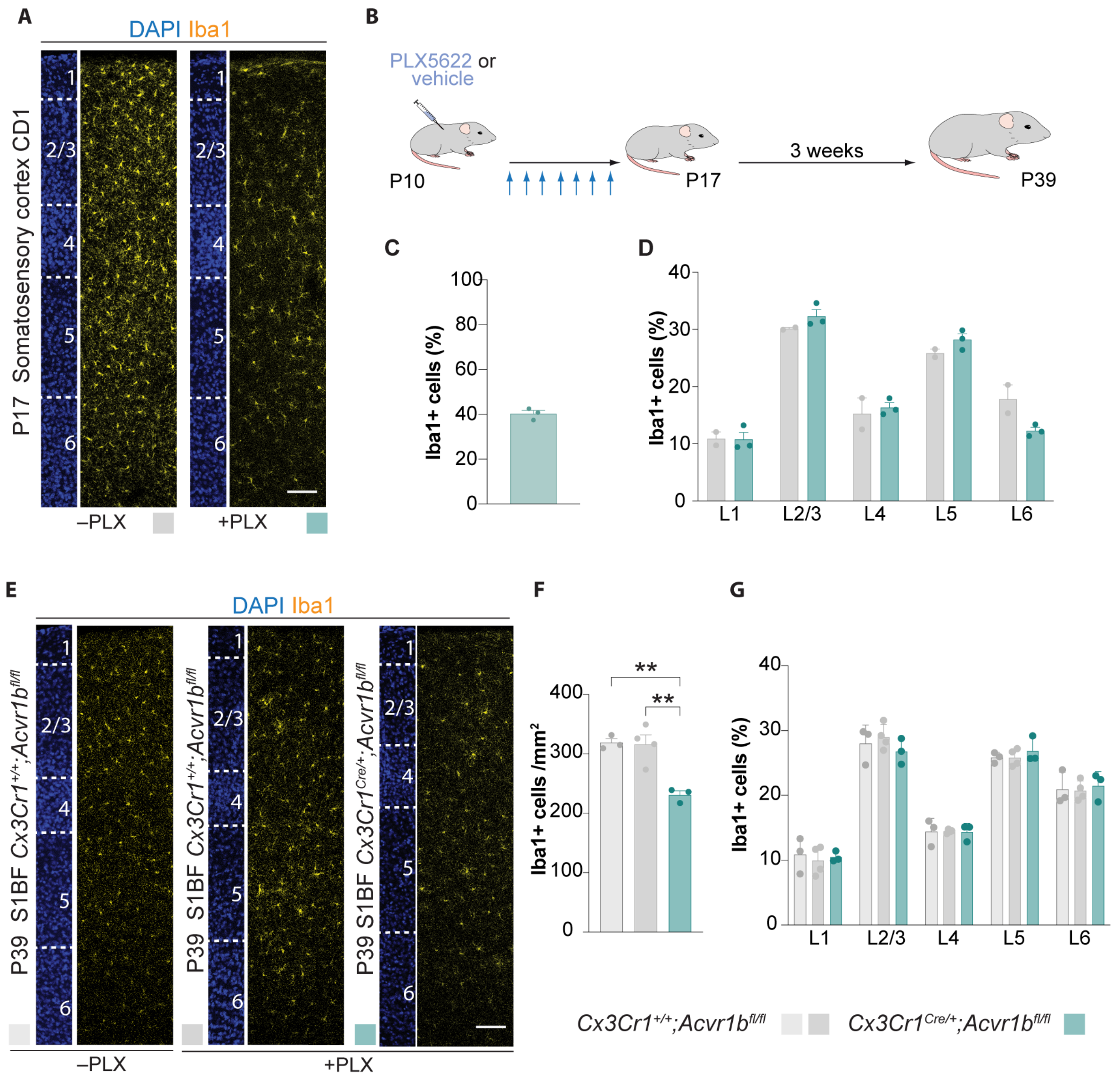

A
DAPI Iba1
P17 Somatosensory cortex CD1
1
2/3
4
5
6
–PLX
+PLX
B
PLX5622 or vehicle
P10
P17
3 weeks
P39
C
Iba1+ cells (%)
D
Iba1+ cells (%)
L1
L2/3
L4
L5
L6
E
DAPI Iba1
P39 S1BF Cx3Cr1+/+;Acvr1bfl/fl
P39 S1BF Cx3Cr1Cre/+;Acvr1bfl/fl
–PLX
+PLX
F
Iba1+ cells /mm²
**
**
G
Iba1+ cells (%)
Cx3Cr1+/+;Acvr1bfl/fl
Cx3Cr1Cre/+;Acvr1bfl/fl

Figure S16. Altered Activin A signaling in macrophages induces changes in microglial numbers in the somatosensory cortex even after microglia depletion

(**A, E**) Coronal sections through the S1BF of *(***A***) CD1* mice at P17 and (**E**) *Cx3Cr1*$^{+/+}$*;Acvr1b*$^{f/f}$ and *Cx3Cr1*$^{Cre/+}$*;Acvrb*$^{f/f}$ mice at P39 treated with PLX5622 or untreated, following immunohistochemistry for Iba1 (yellow). DAPI is shown for counterstaining (blue). (**B**) Schematic of experimental design (**C**) Percentage of microglia after PLX5622 treatment (Control: n = 2, PLX-treated: n = 3) (**D**) Quantification of distribution of Iba1+ cells at P21. Control: n = 2, PLX-treated: n = 3. (**F**) Quantification of Iba1+ cells in the S1BF at P39. 1-way ANOVA (F = 15.45, $**p$ = 0.005 (Control -PLX *vs* Mutant +PLX), $**p$ = 0.004 (Control + PLX *vs* Mutant +PLX), Control -PLX and Mutant +PLX: n = 3, Control +PLX: n = 4. (**G**) Quantification of distribution of Iba1+ cells at P39. Two-way ANOVA. ($F_{interaction}$ = 0.46), Control -PLX and Mutant +PLX: n = 3, Control +PLX: n = 4. For box plot, data points indicate the average cell density in each animal. Scale bars, 100 µm.

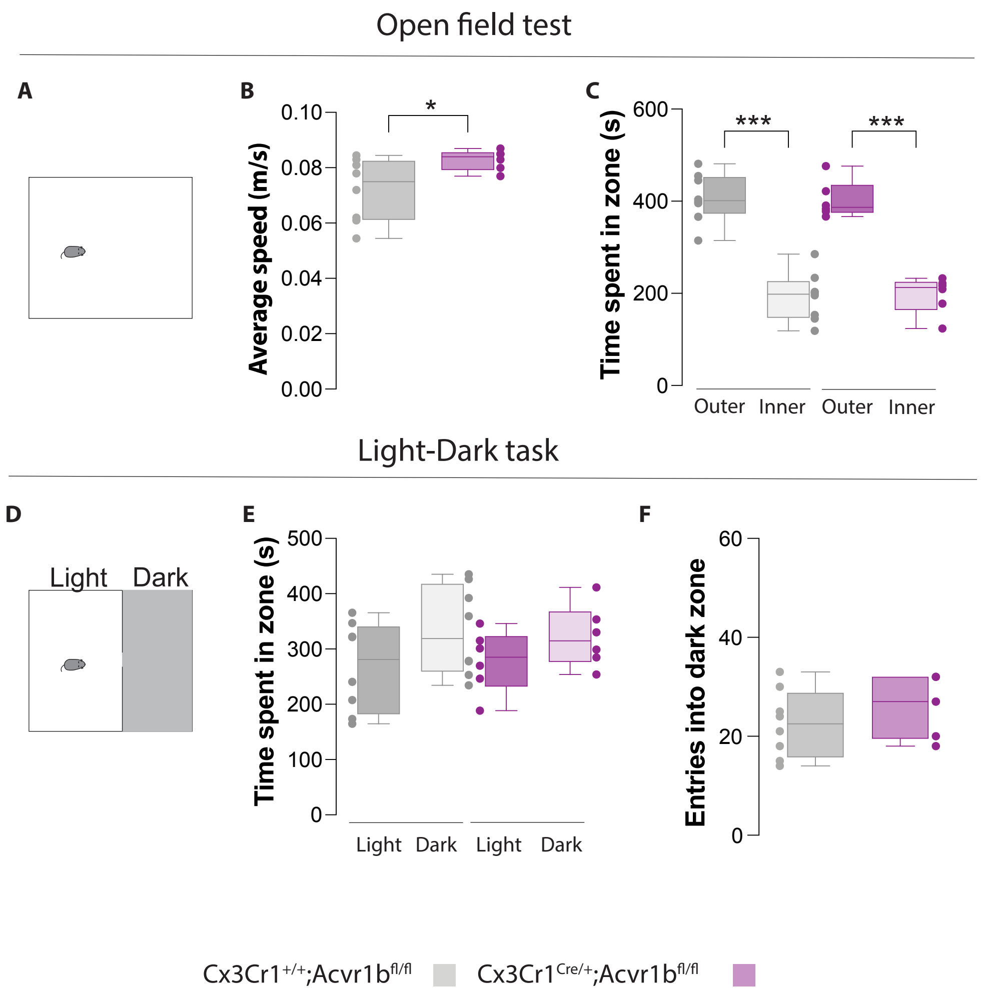
Open field test
A
B
C
Average speed (m/s)
0.10
0.08
0.06
0.04
0.02
0.00
*
Time spent in zone (s)
600
400
200
0
***
***
Outer
Inner
Outer
Inner
Light-Dark task
D
E
F
Light
Dark
Time spent in zone (s)
500
400
300
200
100
0
Light
Dark
Light
Dark
Entries into dark zone
60
40
20
0
Cx3Cr1+/+;Acvr1bfl/fl
Cx3Cr1Cre/+;Acvr1bfl/fl

Figure S17. Dysregulation of pyramidal cell-microglia communication does not alter anxiety-related behaviors

(**A**, **D**) Schematic of experimental design. (**B**) Average speed of *Cx3Cr1*$^{+/+}$*;Acvr1b*$^{f/f}$ and *Cx3Cr1*$^{Cre/+}$*;Acvr1b*$^{f/f}$ mice aged in the open field arena. Mann-Whitney test, *$p = 0.047$, Control: n = 8, Mutant: n = 6. (**C**) Time spent in the outer zone (dark box plots) and inner zone (lighter box plots) by *Cx3Cr1*$^{+/+}$*;Acvr1b*$^{f/f}$ and *Cx3Cr1*$^{Cre/+}$*;Acvr1b*$^{f/f}$. One-way ANOVA with Tukey multiple comparison ($F = 44.95$, ***$p < 0.0001$), Control: n = 8, Mutant: n = 6. (**E**) Time spent in the light zone (dark box plots) and dark zone (lighter box plots) by *Cx3Cr1*$^{+/+}$*;Acvr1b*$^{f/f}$ and *Cx3Cr1*$^{Cre/+}$*;Acvr1b*$^{f/f}$ mice. One-way ANOVA with Tukey multiple comparison ($F = 1.465$), Control: n = 8, Mutant: n = 6. (**F**) Entries into the dark zone by *Cx3Cr1*$^{+/+}$*;Acvr1b*$^{f/f}$ and *Cx3Cr1*$^{Cre/+}$*;Acvr1b*$^{f/f}$ mice in the light-dark arena. Two-tailed unpaired Student's t-test, *$p = 0.022$, Control: n = 8, Mutant: n = 6. For box plots, data points indicate the average measurement for each animal.

A

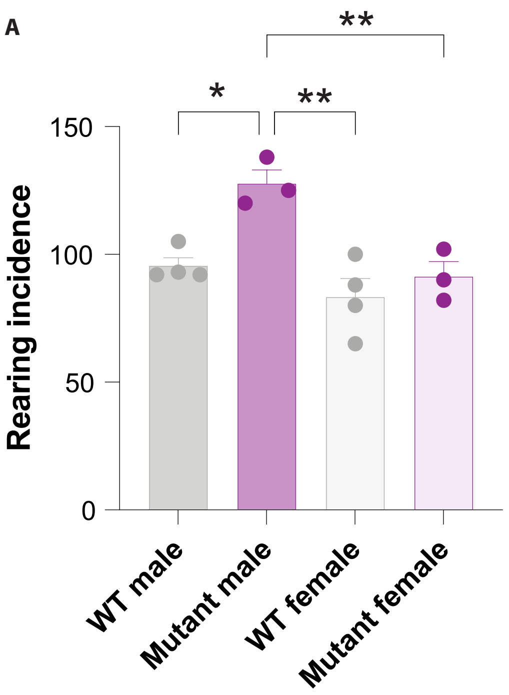
**
*
**
150
100
50
0
Rearing incidence
WT male
Mutant male
WT female
Mutant female

Figure S18. Sex-difference observed in rearing incidences in open-field task

(**A**) Number of incidences of rearing in males (dark box plots) and females (lighter box plots) by *Cx3Cr1*$^{+/+}$*;Acvr1b*$^{f/f}$ (grey box plot, n =8) and *Cx3Cr1*$^{Cre/+}$*;Acvr1b*$^{f/f}$ (magenta box plot, n = 6) aged between 2-4 mice in the open field arena with 10 mins interval. One-way ANOVA with Tukey multiple comparison ($F = 10.70$, *$p = 0.014$, **$p = 0.012$ (conditional mutant males *vs* WT females), **$p = 0.009$ (conditional mutant males *vs* conditional mutant females).

Table S1. Summary of data and statistical analyses, related to Figures 1-6 and Figures S1-S18

**FIGURE 1**	Measurement	Values	N	Statistical	P value
Figure 1B	Ratio of microglia to pyramidal cells (mean ± SEM)	**Motor**: P5: 0.04283 ± 0.0023; P10: 0.16723 ± 0.0028; P15: 0.18928 ± 0.0054; P21: 0.19896 ± 0.0065; P30: 0.14358 ± 0.0064; P42: 0.15873 ± 0.0018; P80: 0.15473 ± 0.00061	[brains] P5: n = 6 (2 males, 4 females), P10: n = 6 (3 males, 3 females), P15: n = 7 (3 males, 4 females), P21: n = 8 (4 males, 4 females), P30: n=4 (2 males, 2 females), P42: n = 8 (3 males, 5 females), P80: n =7 (3 males, 5 females)	1-way Anova	*p* < 0.001 (***)(P5 vs P21, P21 vs P30)
		**Motor (Sex)**: P10 (male) 0.169 ± 0.006 vs P10(Female): 0.165 ± 0.0002, P15(male): 0.196 ± 0.011 vs P15 (Female): ±0.185 ± 0.005, P21 (male): 0.196 ±0.013 vs P21 (female): 0.202 ±0.004, P42 (male): 0.157 ± 0.004 vs P42 (female): 0.160 ±0.001		2-tailed unpaired Student's t-test	NS for all pairs
		**Somatosensory:** P5: 0.04651 ± 0.0024; P10: 0.1728 ± 0.00253; P15: 0.14904 ± 0.0029; P21: 0.15268 ± 0.00307; P30: 0.12830 ± 0.0032; P42: 0.13117 ± 0.0010; P80: 0.12934 ± 0.00154	[brains] P5: n = 6 (2 males, 4 females), P10: n = 6 (3 males, 3 females), P15: n = 7 (3 males, 4 females), P21: n = 8 (4 males, 4 females), P30: n=4 (2 males, 2 females), P42: n = 8 (3 males, 5 females), P80: n =6 (4 males, 2 females)	1-way Anova	*p* < 0.001 (***)(P5 vs P10, P10 vs P30)
		**Somatosensory (Sex)**: P10 (male) 0.177 ± 0.003 vs P10(Female): 0.172 ± 0.0003, P15(male): 0.151 ± 0.005 vs P15 (Female): ±0.148 ± 0.004, P21 (male): 0.148 ±0.004 vs P21 (female): 0.1579 ±0,002, P42 (male): 0.1332 ± 0.002 vs P42 (female): 0.130 ±0.001		2-tailed unpaired Student's t-test	NS for all pairs
		**Auditory:** P5: 0.038287 ± 0.00168; P10: 0.16607 ± 0.00447; P15: 0.18696 ± 0.00235; P21: 0.17154 ± 0.00261; P30: 0.13240 ± 0.00306; P42: 0.13902 ± 0.00306; P80: 0.13709 ± 0.00395	[brains] P5: n = 6 (2 males, 4 females), P10: n = 6 (3 males, 3 females), P15: n = 7 (3 males, 4 females), P21: n = 8 (4 males, 4 females), P30: n=4 (2 males, 2 females), P42: n = 8 (3 males, 5 females), P80: n =6 (4 males, 2 females)	1-way Anova	*p* < 0.001 (***)(P5 vs P15, P15 vs P30)
		**Auditory (Sex)**: P10 (male) 0.165 ± 0.007 vs P10(Female): 0.167 ± 0.007, P15(male): 0.188 ± 0.003 vs P15 (Female): ±0.186 ± 0.003, P21 (male): 0.170 ±0.005 vs P21 (female): 0.173 ±0.002, P42 (male): 0.138 ± 0.002 vs P42 (female): 0.134 ±0.002		2-tailed unpaired Student's t-test	NS for all pairs
		**Visual:** P5: 0.035755 ± 0.00161; P10: 0.11859 ± 0.00404; P15: 0.15984 ± 0.00125; P21: 0.17304 ± 0.00128; P30: 0.12960 ± 0.00337; P42: 0.13114 ± 0.00147; P80: 0.13087 ± 0.00222	[brains] P5: n = 6 (2 males, 4 females), P10: n = 6 (3 males, 3 females), P15: n = 7 (3 males, 4 females), P21: n = 8 (4 males, 4 females), P30: n=4 (2 males, 2 females), P42: n = 6 (3 males, 3 females), P80: n =6 (4 males, 2 females)	1-way Anova	*p* < 0.001 (***)(P5 vs P21, P21vs P30)
		**Visual (Sex)**: P10 (male) 0.116 ± 0.004 vs P10(Female): 0.121 ± 0.007, P15(male): 0.159 ± 0.002 vs P15 (Female): 0.161 ± 0.001, P21 (male): 0.175 ±0.001 vs P21 (female): 0.171 ±0.001, P42 (male): 0.130 ± 0.0006 vs P42 (female): 0.132 ±0.003		2-tailed unpaired Student's t-test	NS for all pairs
Figure 1C	Percentage of ratio variation (mean ± SEM)	**Motor**: P5: =-72.32 ± 1.471; P10: 8.078 ± 1.801; P15: 22.33 ± 3.487; P21: 22.84 ± 5.654; P30: -7.203 ± 4.128; P42: 2.758 ± 1.340; P80: 6.67 x10_9 ± 0.3936	[brains] P5: n = 6, P10: n = 6, P15: n = 7, P21: n = 8, P30: n=4, P42: n = 6, P80: n =6		
		**Somatosensory:** P5: -64.05 ± 1.819; P10: 33.66 ± 1.605; P15: 15.22 ± 2.167; P21: 18.04 ± 2.371; P30: -0.817 ± 2.480; P42: 1.473 ± 0.9306; P80: 01x10_8 ± 1.191	[brains] P5: n = 6, P10: n = 5, P15: n = 7, P21: n = 8, P30: n=4, P42: n = 6, P80: n =6		
		**Auditory:** P5: -72.07 ± 1.229; P10: 21.14 ± 3.262; P15: 36.37 ±1.715; P21: 25.13 ± 1.906; P30: -3.426 ± 2.231; P42: 1.408 ± 1.096; P80: 6.667 x10_9 ± 2.883	[brains] P5: n = 6, P10: n = 6, P15: n = 7, P21: n = 8, P30: n=4, P42: n = 6, P80: n =6		
		**Visual:** P5: -72.68 ± 1.233; P10: -9.382 ± 3.090; P15: 22.36 ± 1.074; P21: 30.04 ± 1.791; P30: -0.972 ± 2.576; P42: 0.2078 ± 1.121; P80: 6.667 x 10_9 ± 1.701	[brains] P5: n = 6, P10: n = 6, P15: n = 7, P21: n = 8, P30: n=4, P42: n = 6, P80: n =6		
**FIGURE 2**	Measurement	Values	N	Statistical	P value
Figure 2E	Iba1+ cells (mean ± SEM)	**Iba1+**: hm3Dq-CNO: 669.03 ± 40.41; hm3Dq+CNO: 812.57 ± 32.93	[brains] n = 8 for both groups	2-tailed unpaired Student's t-test	*p* = 0.016 (*)
Figure 2F	Percentage of Ki67+ Iba1+ cells (mean ± SEM)	**Ki67+ Iba1+**: hm3Dq-CNO: 100 ± 15.53; hm3Dq+CNO: 167.89 ± 9.59	[brains] n = 5 for both groups	2-tailed unpaired Student's t-test	*p* = 0.006 (**)
Figure 2H	Cumulative distribution P2RY12+ Iba1+ cells (mean ± SEM)	**P2RY12+ Iba1+ (fraction)**: hm3Dq-CNO: Bin 0: 0.00, Bin 20: 0.36, Bin 40: 0.63, Bin 60: 0.81, Bin 80: 0.91, Bin 100: 0.96, Bin 120: 0.98, Bin 140: 1.00; hM3Dq+CNO: Bin 0: 0.02, Bin 20: 0.47, Bin 40: 0.83, Bin 60: 0.96, Bin 80: 0.98, Bin 100: 1.00, Bin 120: 1.00, Bin 140:1.00	[brains] n = 6 for both groups	Kolmogorov-Smirnov test	*p* < 0.001 (***)
Figure 2L	Iba1+ cells (mean ± SEM)	**Iba1+**: unplucked contralateral: 694.09 ± 16.05, unplucked ipsilateral : 708.15 ± 16.40; plucked contralateral: 575.95 ± 15.60, unplucked ipsilateral : 690.55 ± 25.97;	[brains] n = 6 for all groups	1-way Anova	*p* = 0.0015 (**) (unplucked contralateral vs plucked contralateral), *p* = 0.0004 (***)(unplucked ipsilateral v plucked contralatera); *p* = 0.0020 (**) (plucked contralateral vs plucked ipsilateral)

Figure 2M	Ki67+ Iba1+ percentage (mean ± SEM)	**Iba1+ Ki67+**: unplucked contralateral: 100 ± 7.31, unplucked ipsilateral : 77.93 ± 9.13; plucked contralateral: 17.90 ± 6.67, unplucked ipsilateral : 70.03 ± 4.92;	[brains] n = 6 for both groups	1-way Anova	*p* < 0.0001 (***) (unplucked contralateral vs plucked contralateral), *p* < 0.0001 (***)(unplucked ipsilateral v plucked contralatera); *p* = 0.0003 (***) (plucked contralateral vs plucked ipsilateral)
**FIGURE 3**	Measurement	Values	N	Statistical	P value
Figure 3C	Iba1+ cells (mean ± SEM)	**Iba1+**: LD: 558.108 ± 11.819; DD: 610.940 ±7.431	[brains] n = 12 for both groups	2-tailed unpaired Student's t-test	*p* = 0.0010 (**)
Figure 3D	Iba1+ cells (mean ± SEM)	**Iba1+**: LD: 708.119 ± 3.096; DD: 722.075 ± 8.023	[brains] n = 4 for both groups	2-tailed unpaired Student's t-test	*NS*
Figure 3F	Iba1+ cells volume (mean ± SEM)	**Iba1+ cell volume**: LD: 1.98 x 10$_5$± 0.123; DD : 1.400 x 105± 0.052	[Cells] LD: n = 20 from 5 mice, DD: n = 19 from 6 mice.	Mann Whitney	*p* = 0.0024 (**)
Figure 3G	Iba1+ scholl analysis (mean ± SEM)	**Interactions**: **LD**: Radius (µm) -, 5: 7.23 ± 0.089, 6:19.47 ± 0.67, 7: 12.23 ± 1.00, 8: 12.83 ± 0.99; 9: 13.92 ± 1.81, 10: 15.02 ± 1.39, 11: 16.20 ± 1.51, 12: 18.35 ± 1.97, 13: 18.43 ± 2.75, 14: 18.98 ± 1.95, 15: 19.4 ± 2.35, 16: 20.97 ± 2.31, 17: 22.58 ± 1.91, 18: 24.85 ± 2.38, 19: 23.83 ± 2.50, 20: 23.82 ± 1.80, 21: 22.67 ± 2.82, 22: 22.53 ± 3.52, 23: 21.17 ± 4.65, 24: 21.17 ± 4.55, 25: 20.12 ± 4.69, 26: 18.05 ± 3.23, 27: 17.3 ± 2.39, 28: 16.78 ± 3.49, 29: 15.55 ± 2.91, 30: 13.4 ± 2.87, 31: 11.83 ± 2.30, 32: 11.43 ± 2.19, 33: 8.67 ± 1.62, 34: 7.2 ± 1.63, 35: 6.82 ± 1.17, 36: 6.98 ± 0.91, 37: 5.7 ± 1.25, 38: 4.52 ± 1.44, 39: 3.48 ± 0.83, 40: 2.4 ± 0.38, 41: 1.62 ± 0.55, 42: 1.55 ± 0.97, 43: 0.72 ± 0.42, 44: 0.43 ± 0.32, 45: 0.62 ± 0.40, 46: 0.35 ± 0.24, 47: 0.05 ± 0.05, 48: 0 ± 0, 49: 0 ± 0, 50: 0 ± 0, 51: 0 ± 0, 52: 0 ± 0, 53: 0 ± 0, 54: 0 ± 0, 55: 0 ± 0 **DD:** 5: 6.88 ± 0.54, 6: 9.26 ± 0.74, 7: 10.78 ± 0.85, 8: 13.10 ± 1.31; 9: 13.22 ± 0.66, 10: 14.10 ± 1.85, 11: 15.03 ± 1.84, 12: 17.44 ± 1.25, 13: 17.41 ± 0.99, 14: 18.43 ± 1.87, 15: 18.97 ± 2.28, 16: 20.68 ± 1.44, 17: 20.87 ± 2.67, 18: 21.76 ± 1.85, 19: 21.17 ± 2.54, 20: 20.10 ± 3.27, 21: 20.60 ± 2.04, 22: 19.94 ± 1.82, 23: 19.4 ± 2.08, 24: 19.78 ± 2.04, 25: 17.23 ± 2.67, 26: 15.44 ± 2.20, 27: 14.08 ± 1.09, 28: 11.35 ± 1.34, 29: 11.69 ± 1.68, 30: 8.93 ± 1.35, 31: 8.51 ± 1.38, 32: 7.03 ± 1.19, 33: 6.56 ± 1.01, 34: 5.97 ± 1.13, 35: 5.41 ± 0.73, 36: 5.63 ± 0.75, 37: 4.59 ± 0.34, 38: 4.60 ± 0.73, 39: 3.28 ± 0.38, 40: 2.75 ± 0.48, 41: 2.60 ± 0.45, 42: 1.4 ± 0.37, 43: 1.3 ± 0.14, 44: 1.36 ± 0.48, 45: 1.15 ± 0.31, 46: 1.01 ± 0.56, 47: 0.83 ± 0.35, 48: 0.65 ± 0.36, 49: 0.33 ± 0.33, 50: 0.083 ± 0.083, 51: 0.42 ± 0.42, 52: 0.33 ± 0.33, 53: 0.5 ± 0.517, 54: 0.13 ± 0.13, 55: 0 ± 0	[brains] n = 4 for both groups	2-way Anova	F_{group} (1,366): 12.36, *p* = 0.0005 (***)
Figure 3H	Percentage of Ki67+ Iba1+ cells (mean ± SEM)	Ki6**7+ Iba1+**: LD: 0.202 ± 0.032; DD: 0.243 ± 0.038	[brains] n = 12 for both groups	2-tailed unpaired Student's t-test	*NS*
Figure 3I	Cumulative distribution P2RY12+ immunofluorescence intensity	**Iba1+**:LD: Bin 0: 0, Bin 30.235, Bin 60: 0.672, Bin 90: 0.873, Bin 120, 0.943, Bin 150: 0.984, Bin 180: 0.993, Bin 210: 1.000, Bin 240: 1.000; DD: Bin 0: 0, Bin 30: 0.322, Bin 60: 0.765, Bin 90: 0.930, Bin 120: 0.975, Bin 150: 0.0987, Bin 180: 0.994, Bin 210, 0.999, Bin 240: 1.000	[Cells] LD: n = 668 from 4 mice, DD: n = 711 from 4 mice.	Kolmogorov-Smirnov test	*p* < 0.001 (***)
**FIGURE 4**	Measurement	Values	N	Statistical	P value
Figure 4D	Cumulative distribution ActivinA immunofluorescence intensity	**ActivinA (fraction)**: **P5**: Bin 100: 0, Bin 200: 0.023, Bin 300: 0.2, Bin 400: 0.43, Bin 500: 0.71, Bin 600: 0.84, Bin 700: 0.93, Bin 800: 0.977, Bin 900: 0.99, Bin 1000: 0.997, Bin 1100: 0.997, Bin 1200: 1, Bin 1300: 1, Bin 1400: 1, Bin 1500: 1; **P10**: Bin 100: 0, Bin 200: 0.005, Bin 300: 0.066, Bin 400: 0.311, Bin 500: 0.56, Bin 600: 0.70, Bin 700: 0.83, Bin 800: 0.917, Bin 900: 0.96, Bin 1000: 0.98, Bin 1100: 0.991, Bin 1200: 0.997, Bin 1300: 0.997, Bin 1400: 1, Bin 1500: 1; **P15**: Bin 100: 0.0039, Bin 200: 0.074, Bin 300: 0.23, Bin 400: 0.46, Bin 500: 0.64, Bin 600: 0.74, Bin 700: 0.82, Bin 800: 0.87, Bin 900: 0.94, Bin 1000: 0.96, Bin 1100: 0.984, Bin 1200: 0.988, Bin 1300: 0.992, Bin 1400: 0.996, Bin 1500: 1; **P21**: Bin 100: 0, Bin 200: 0.025, Bin 300: 0.23, Bin 400: 0.50, Bin 500: 0.81, Bin 600: 0.92, Bin 700: 0.97, Bin 800: 0.99, Bin 900: 1, Bin 1000: 1, Bin 1100: 1, Bin 1200: 1, Bin 1300: 1, Bin 1400: 1, Bin 1500: 1.	[Cells] P5: n = 310 from 4 mice, P10: n = 366 from 4 mice, P15: n = 257 from 4 mice, P21: n =236 from 4 mice	Kruskal Wallis test	*P5 vs P10: p < 0.001 (***); P10 vs P15: p < 0.001 (***); P10 vs P21: p < 0.001 (***)*
Figure 4E	pSMAD3+ Iba1+ cells (mean ± SEM)	**pSMAD3+ Iba1+** : P5: 22.09 ± 5.08; P10: 61.06 ± 5.91; P15: 36.43 ± 7.31; P21: 18.21 ± 4.14	[brains] P5: n = 5; P10: n = 6; P15: n = 8; P21: n = 7 mice	1-way Anova	*p* = 0.0024 (**); *p* = 0.035 (*); *p* = 0.0003 (***)

Figure 4G	Activin level (mean ± SEM)	**Iba1+ soma volume**: LD: 13.17 ± 0.97; DD: 64 ± 8.57	[brains] LD: n = 6; DD: n = 5 mice	Mann Whitney	*p* = 0.0043
Figure 4I	pSMAD3+ Iba1+ cells (mean ± SEM)	**pSMAD3+ Iba1+** : LD: 26.20 ± 4.81; DD: 49.97 ± 6.97	[brains] LD: n = 6; DD: n = 5 mice	2-tailed unpaired Student's t-test	*p* = 0.025 (*)
**FIGURE 5**	Measurement	Values	N	Statistical	P value
Figure 5E	Iba1+ cells (mean ± SEM)	**Iba1+**: Control: 445.95 ± 12.46; Mutant: 354.52 ± 9.15	[brains] Control: n = 7, Mutant: n = 4	2-tailed unpaired Student's t-test	*p* = 0.0007 (***)
Figure 5F	Iba1+ cells volume (mean ± SEM)	**Iba1+ cell volume**: Control: 0.618 x 10_{5}± 0.04; Mutant: 0.893 x 105± 0.056	[Cells] n = 20 from 4 mice for both groups	2-tailed unpaired Student's t-test	*p* = 0.0009 (***)
Figure 5G	Iba1+ scholl analysis (mean ± SEM)	**Interactions**: Control: Radius (µm) -, 5: 7.36 ± 0.69, 6: 9.09 ± 0.75, 7: 9.51 ± 0.71, 8: 10.65 ± 0.5; 9: 10.88 ± 1.16, 10: 12.10 ± 0.72, 11: 13.45 ± 0.34, 12: 13.26 ± 1.30, 13: 14.39 ± 0.9, 14: 13.80 ± 1.41, 15: 14.61 ± 0.36, 16: 14.96 ± 0.53, 17: 14.70 ± 0.41, 18: 14.8 ± 0.82, 19: 12.81 ± 1.33, 20: 12.64 ± 1.98, 21: 12.62 ± 2.10, 22: 11.16 ± 1.93, 23: 9.46 ± 2.00, 24: 8.80 ± 1.93, 25: 8.49 ± 1.344, 26: 7.30 ± 1.54, 27: 6.16 ± 1.42, 28: 5.37 ± 1.11, 29: 4.40 ± 0.87, 30: 3,29 ± 0.96, 31: 3.06 ± 0.96, 32: 3.25 ± 0.52, 33: 2.69 ± 0.61, 34: 2.47 ± 0.51, 35: 1,37 ± 0.46, 36: 1.12 ± 0.58, 37: 0.64 ± 0.27, 38: 0.56 ± 0.20, 39: 0.44 ± 0.17, 40: 0.36 ± 0.18, 41: 0.29 ± 0.12, 42: 0.24 ± 0.14, 43: 0.21 ± 0.12, 44: 0.22 ± 0.14, 45: 0.22 ± 0.14 MUTANT: 5: 6.04 ± 0.38, 6: 9.26 ± 0.32, 7: 10.95 ± 0.42, 8: 12 ± 0.31; 9: 12.54 ± 0.73, 10: 14.38 ± 0.76, 11: 15.63 ± 0.83, 12: 16.04 ± 0.49, 13: 16.67 ± 0.46, 14: 20.08 ± 0.31, 15: 19.92 ± 1.03, 16: 17.21 ± 1.23, 17: 19.08 ± 0.17, 18: 17.29 ± 1.17, 19: 15.96 ± 1.24, 20: 16.13 ± 1.34, 21: 14.96 ± 1.58, 22: 14.54 ± 1.09, 23: 12.58 ± 1.64, 24: 11.92 ± 1.89, 25: 11.33 ± 1.97, 26: 10.71 ± 1.92, 27: 9.54 ± 1.33, 28: 7.96 ± 1.70, 29: 6.71 ± 1.78, 30: 5.63 ± 1.48, 31: 4.5 ± 1.27, 32: 4 ± 1.40, 33: 3.5 ± 1.22, 34: 2.46 ± 0.85, 35: 2 ± 0.71, 36: 1.71 ± 0.63, 37: 1.33 ± 0.55, 38: 1.25 ± 0.41, 39: 1 ± 0.41, 40: 0.71 ± 0.25, 41: 0.29 ± 0.14, 42: 0.167 ± 0.07, 43: 0.13 ± 0.08, 44: 0.083 ± 0.0833, 45: 0.042 ± 0.042, 46: 0.042 ± 0.042, 47:0.042 ± 0.042, 48: 0.042 ± 0.042, 49: 0.042 ± 0.042, 50: 0 ± 0 , 51: 0 ± 0 , 52: 0 ± 0 , 53: 0 ± 0 , 54: 0 ± 0 , 55: 0 ± 0	[brains] n = 4 for both groups	2-way Anova	F_{group} (1,282): 64.42, *p* < 0.0001 (***)
Figure 5H	Percentage of excitatory synapse (mean ± SEM)	**Homer and Bassoon (%)**:Control: 1.033 ± 0.034; Mutant: 1.234 ± 0.096	[brains] Control: n = 7, Mutant: n = 4	2-tailed unpaired Student's t-test	*p* = 0.0391 (*),
Figure 5I	Number of engulfed bassoon puncta (mean ± SEM)	Engulfed bassoon (%) : Control: 0.172 ± 0.2; Mutant: 0.118 ± 0.005	[brains] n = 4 for both groups	2-tailed unpaired Student's t-test	*p* = 0.0374 (*),
**FIGURE 6**	Measurement	Values	N	Statistical	P value
Figure 6B	Total distance travelled (mean ± SEM)	**Distance**: Control: 42.80 ± 2.07; Mutant: 50.26 ± 1.37	[Mice] Control: n = 8, Mutant: n = 6	2-tailed unpaired Student's t-test	*p* = 0.017 (*)
Figure 6C	Rearing incidences (mean ± SEM)	**Rearing incidences**: Control: 89.38± 4.37; Mutant: 109.5 ± 8.86	[Mice] Control: n = 8, Mutant: n = 6	2-tailed unpaired Student's t-test	*p* = 0.048 (*)
Figure 6D	Jumping incidences (mean ± SEM)	**Jumping incidences**: Control: 2.88± 1.51; Mutant: 12.67 ± 6.37	[Mice] Control: n = 8, Mutant: n = 6	Mann Whitney	*p* = 0.1124 (NS)
Figure 6F	Total exploration time (mean ± SEM)	**Time**: Control: 49.52 ± 2.05; Mutant: 49.12 ± 4.61	[Mice] Control: n = 8, Mutant: n = 6	2-tailed unpaired Student's t-test	*p* = 0.9335 (NS)
Figure 6G	Time exploring objects (mean ± SEM)	**Time**: Control-Familiar: 15.68 ± 1.29;Control-Novel: 33.83 ± 1.30; Mutant - Familiar: 20:85 ± 2.62; Mutant -Novel: 28.28 ± 2.10	[Mice] Control: n = 8, Mutant: n = 6	1-way Anova	F = 22.58, *p* < 0.0001 (***)
Figure 6H	Discrimination index (mean ± SEM)	**Discrimination index**: Control: 0.371± 0.034; Mutant: 0.169 ± 0.039	[Mice] Control: n = 8, Mutant: n = 6	2-tailed unpaired Student's t-test	*p* = 0.021 (**)
Figure 6J	Total exploration time (mean ± SEM)	**Time**: Control: 71.84 ± 8.84; Mutant: 49.96 ± 4.63	[Mice] Control: n = 8, Mutant: n = 6	2-tailed unpaired Student's t-test	*p* = 0.071 (NS)
Figure 6K	Time exploring stimuli (mean ± SEM)	**Time**: Control-Object: 16.89 ± 1.76; Control-Mice: 54.95 ± 7.47; Mutant - Object: 18.89 ± 3.25; Mutant -Mice: 31.07 ± 3.20	[Mice] Control: n = 8, Mutant: n = 6	1-way Anova	F = 14.41, *p* < 0.0001 (***)
Figure 6L	Discrimination index (mean ± SEM)	**Discrimination index**: Control: 0.511± 0.044; Mutant: 0.160.252 ± 0.094	[Mice] Control: n = 8, Mutant: n = 6	2-tailed unpaired Student's t-test	*p* = 0.0182 (**)
**FIGURE S1**	Measurement	Values	N	Statistical	P value
		**Motor**: P5: 132.71 ± 7.06; P10: 517.37 ± 23.70; P15: 500.64 ± 13.89; P21: 443.49 ± 11.19; P30: 389.98 ± 12.54; P42: 401.22 ± 7.45; P80: 330.61 ± 10.42	[brains] P5: n = 6 (2 males, 4 females), P10: n = 6 (3 males, 3 females), P15: n = 7 (3 males, 4 females), P21: n = 8 (4 males, 4 females), P30: n=4 (2 males, 2 females), P42: n = 8 (3 males, 5 females), P80: n =7 (3 males, 5 females)	1-way Anova	F = 93.06, *p* < 0.0001 (***)

Figure S1B	Iba1+ cells (mean ± SEM)	**Somatosensory:** P5: 146.90 ± 5.79; P10: 488.99 ± 33.34; P15: 452.45 ± 33.34; P21: 391.97 ± 7.74; P30: 440.02 ± 26.36; P42: 360.46 ± 4.33; P80: 363.48 ± 8.81	[brains] P5: n = 6 (2 males, 4 females), P10: n = 6 (3 males, 3 females), P15: n = 7 (3 males, 4 females), P21: n = 8 (4 males, 4 females), P30: n=4 (2 males, 2 females), P42: n = 8 (3 males, 5 females), P80: n =6 (4 males, 2 females)	1-way Anova	F = 71.91, *p* < 0.0001 (***)
		**Auditory:** P5: 91.41 ± 4.01; P10: 396.28 ± 25.12; P15: 398.76 ± 16.91; P21: 319.27 ± 10.53; P30: 380.96 ± 12.77; P42: 304.76 ± 4.94; P80: 324.36 ± 3.04	[brains] P5: n = 6 (2 males, 4 females), P10: n = 6 (3 males, 3 females), P15: n = 7 (3 males, 4 females), P21: n = 8 (4 males, 4 females), P30: n=4 (2 males, 2 females), P42: n = 8 (3 males, 5 females), P80: n =6 (4 males, 2 females)	1-way Anova	F = 62.60, *p* < 0.0001 (***, P5 vs P10), *p* = 0.0002 (***, P10 vs P42)
		**Visual:** P5: 75.71 ± 3.07; P10: 266.09 ± 13.97; P15: 331.35 ± 10.62; P21: 314.47 ± 7.93; P30: 273.95 ± 8.67; P42: 282.45 ± 4.23; P80: 285.54 ± 13.48	[brains] P5: n = 6 (2 males, 4 females), P10: n = 6 (3 males, 3 females), P15: n = 7 (3 males, 4 females), P21: n = 8 (4 males, 4 females), P30: n=4 (2 males, 2 females), P42: n = 6 (3 males, 3 females), P80: n =6 (4 males, 2 females)	1-way Anova	F = 76.57, p < 0.0001 (***, P5 vs P15), p = 0.0047 (**, P15 vs P42)
Figure S1C	mCherry+ cells (mean ± SEM)	**Motor**: P5: 3086.32 ± 133.69; P10: 2937.78 ± 83.97; P15: 2635.01 ± 74.59; P21: 2218.73 ± 50.62; P30: 2766.69 ± 186.94; P42: 2509.42 ± 52.44; P80: 2129.84 ± 68.57	[brains] P5: n = 6 (2 males, 4 females), P10: n = 6 (3 males, 3 females), P15: n = 7 (3 males, 4 females), P21: n = 8 (4 males, 4 females), P30: n=4 (2 males, 2 females), P42: n = 8 (3 males, 5 females), P80: n =7 (3 males, 5 females)	1-way Anova	F = 16.17, *p* = 0.015 (*)
		**Somatosensory:** P5: 2901.64 ± 156.23; P10: 3077.30 ± 46.43; P15: 2932.72 ± 48.96; P21: 2549.81 ± 43.43; P30: 3388.73 ± 112.88; P42: 2743.06 ± 35.63; P80: 2795.54 ± 66.65	[brains] P5: n = 6 (2 males, 4 females), P10: n = 6 (3 males, 3 females), P15: n = 7 (3 males, 4 females), P21: n = 8 (4 males, 4 females), P30: n=4 (2 males, 2 females), P42: n = 8 (3 males, 5 females), P80: n =6 (4 males, 2 females)	1-way Anova	F = 10.30, *NS*
		**Auditory:** P5: 2373.30 ± 47.19; P10: 2313.71 ± 102.34; P15: 2120.87 ± 75.34; P21: 1850.47 ± 49.27; P30: 2857.48 ± 119.43; P42: 2170.46 ± 19.44; P80: 2367.46 ± 65.30	[brains] P5: n = 6 (2 males, 4 females), P10: n = 6 (3 males, 3 females), P15: n = 7 (3 males, 4 females), P21: n = 8 (4 males, 4 females), P30: n=4 (2 males, 2 females), P42: n = 8 (3 males, 5 females), P80: n =6 (4 males, 2 females)	1-way Anova	F = 18.33, *NS*
		**Visual:** P5: 2062.66 ± 36.08; P10: 2232.73 ± 51.09; P15: 2088.24 ± 57.69; P21: 1807.54 ± 42.64; P30: 2131.64 ± 111.53; P42: 2137.31 ± 16.86; P80: 2177.90 ± 96.94	[brains] P5: n = 6 (2 males, 4 females), P10: n = 6 (3 males, 3 females), P15: n = 7 (3 males, 4 females), P21: n = 8 (4 males, 4 females), P30: n=4 (2 males, 2 females), P42: n = 6 (3 males, 3 females), P80: n =6 (4 males, 2 females)	1-way Anova	F = 6.332, *NS*
**FIGURE S2**	Measurement	Values	N	Statistical	P value
Figure S2A	Percentage of Ki67+ Iba1+ cells (mean ± SEM)	**P5**: Motor: 38.47 ± 3.32; Somatosensory: 56.51 ± 4.57, Auditory: 44.30 ± 1.82; Visual: 43.79 ± 1.24	[brains] n = 6 for all groups	1-way Anova	*p* = 0.0022 (**) (motor vs somatosensory), *p* = 0.0449 (*) (somatonsensory vs auditory), *p* = 0.0351 (*) (somatonsensory vs visual)
		**P10**: Motor: 78.78 ± 5.56; Somatosensory: 42.18 ± 3.28, Auditory: 79.20 ± 7.01; Visual: 68.92 ± 5.10	[brains] n = 8 for both groups	1-way Anova	*p* = 0.0003 (***) (motor vs somatosensory), *p* = 0.0002 (***) (somatonsensory vs auditory), *p* = 0.0081 (**) (somatonsensory vs visual)

		**P15**: Motor: 10.34 ± 1.28; Somatosensory: 1.98 ± 0.20, Auditory: 2.17 ± 0.49; Visual: 19.53 ± 3.10	[brains] n = 4 for both groups	1-way Anova	*p* = 0.0306 (*) (motor vs somatosensory), p = 0.0235 (*) (motor vs auditory), p = 0.0111 (*) (motor vs visual),*p* < 0.0001 (***) (somatonsensory vs visual), *p* < 0.0001 (***) (auditory vs visual),
		**P21:** Motor: 0.52 ± 0.12; Somatosensory: 0.35 ± 0.12, Auditory: 1.04 ± 0.5; Visual: 1.56 ± 0.33	[brains] n = 7 for both groups	1-way Anova	
Figure S2B	Percentage of P2RY12+ Iba1+ cells (mean ± SEM)	**P5**: Motor: 36.92 ± 3.39; Somatosensory: 59.14 ± 3.05, Auditory: 40.71 ± 2.64; Visual: 35.89 ± 4.48	[brains] n = 6 for all groups	1-way Anova	*p* = 0.0010 (**) (motor vs somatosensory), *p* = 0.0061 (**) (somatonsensory vs auditory), *p* = 0.0006 (***) (somatonsensory vs visual),
		**P10**: Motor: 64.75 ± 7.58; Somatosensory: 90.59 ± 2.26, Auditory: 75.06 ± 2.63; Visual: 70.07 ± 2.60	[brains] n = 6 for both groups	1-way Anova	*p* = 0.0024 (**) (motor vs somatosensory) *p* = 0.016 (*) (somatonsensory vs visual),
		**P15**: Motor: 99.63 ± 0.12; Somatosensory: 99.74 ± 0.10, Auditory: 99.44 ± 0.22; Visual: 99.42 ± 0.19	[brains] n = 8 for both groups	1-way Anova	
		**P21:** Motor: 100 ± 0.00; Somatosensory: 99.79 ± 0.16, Auditory: 100 ± 9; Visual: 99.65 ± 0.18	[brains] n = 7 for both groups	1-way Anova	
**FIGURE S3**	Measurement	Values	N	Statistical	P value
Figure S3	Iba1+ cells (mean ± SEM)	**Iba1+**: -CNO: 585.70 ± 3.33; +CNO: 592.13 ± 6.77	[brains] n = 3 for both groups	2-tailed unpaired Student's t-test	NS
**FIGURE S4**	Measurement	Values	N	Statistical	P value
Figure S4B	Iba1+ cells (mean ± SEM)	**Iba1+**: hm3Dq-CNO: L1: 10.88 ± 0.42, L2/3: 26.88 ± 0.58; L4: 14.42 ± 0.28, L5: 26.00 ± 0.57, L6: 21.82 ± 0.73; hm3Dq+CNO: L1: 10.65 ± 0.29, L2/3: 26.11 ± 0.35; L4: 13.86 ± 0.63, L5: 25.25 ± 0.53, L6: 24.02 ± 0.48	[brains] n = 8 for both groups	2-way Anova	*p* = 0.0143 (*)
Figure S4C	Iba1+ cells (% ± SEM)	**Iba1+**: unplucked contralateral: L1: 12.96 ± 0.60, L2/3: 27.19 ± 0.57; L4: 15.89 ± 0.38, L5: 25.24 ± 0.47, L6: 18.73 ± 0.83; plucked contralateral: L1: 12.74 ± 0.58, L2/3: 27.53 ± 0.22; L4: 16.55 ± 0.30, L5: 25.39 ± 0.50, L6: 17.80 ± 0.98	[brains] n = 6 for both groups	2-way Anova	NS
**FIGURE S5**	Measurement	Values	N	Statistical	P value
Figure S5C	Iba1+ cells (mean ± SEM)	**Iba1+**: hm3Dq-CNO: 621.07 ± 16.04; hm3Dq+CNO: 794.35 ± 54.33	[brains] n = 4 for both groups	2-tailed unpaired Student's t-test	*p* = 0.0223 (*)
Figure S5D	Iba1+ cells (% ± SEM)	**Iba1+**: hm3Dq-CNO: L1: 16.85 ± 1.38, L2/3: 21.42 ± 2.27; L4: 12.60 ± 1.32, L5: 24.94 ± 0.85, L6: 24.19 ± 2.22; hm3Dq+CNO: L1: 17.81 ± 1.35, L2/3: 21.13 ± 1.76; L4: 12.97 ± 1.19, L5: 24.79 ± 0.73, L6: 23.31 ± 0.69	[brains] n = 4 for both groups	2-way Anova	NS
**FIGURE S6**	Measurement	Values	N	Statistical	P value
Figure S6B	Sox9+ cells (mean ± SEM)	**Iba1+**: hm3Dq-CNO: 639.14 ± 33.81; hm3Dq+CNO: 786.42 ± 19.36	[brains] n = 4 for both groups	2-tailed unpaired Student's t-test	*p* = 0.0092 (**)
Figure S6C	Iba1+ cells (% ± SEM)	**Iba1+**: hm3Dq-CNO: L1: 14.38 ± 1.23, L2/3: 24.66 ± 1.00; L4: 16.69 ±. 0.67, L5: 23.61 ± 1.02, L6: 20.67 ± 0.5; hm3Dq+CNO: L1: 15.69 ± 1.14, L2/3: 26.64 ± 0.70; L4: 16.41 ± 0.36, L5: 22.56 ± 1.21, L6: 18.71 ± 1.03	[brains] n = 4 for both groups	2-way Anova	NS
**FIGURE S7**	Measurement	Values	N	Statistical	P value
Figure S7D	Iba1+ cells (mean ± SEM)	**Iba1+**: hm3Dq-CNO: 208.27 ± 3.4356; hm3Dq+CNO: 279.14 ± 20.42	[brains] n = 5 for both groups	Mann Whitney	*p* = 0.0079 (**)
Figure S7E	Iba1+ cells (% ± SEM)	**Iba1+**: hm3Dq-CNO: L1: 12.28 ± 0.38, L2/3: 27.89 ± 0.42; L4: 11.50 ±. 0.15, L5: 27.47 ± 0.93, L6: 20.87 ± 0.43; hm3Dq+CNO: L1: 9.66 ± 0.95, L2/3: 29.91 ± 0.50; L4: 13.26 ± 0.45, L5: 25.34 ± 0.38, L6: 21.81 ± 0.79	[brains] n = 5 for both groups	2-way Anova	NS
Figure S7F	Iba1+ cells volume (mean ± SEM)	**Iba1+ cell volume**: hm3Dq-CNO: 1.321 x 10_5± 0.103; hm3Dq+CNO: 0.964 x 105± 0.066	[Cells] hm3Dq-CNO: n = 23 from 5 mice, hm3Dq+CNO: n = 25 from 5 mice.	2-tailed unpaired Student's t-test	*p* = 0.0048 (**)

Figure S7G	Iba1+ scholl analysis (mean ± SEM)	**Interactions**: **hm3Dq-CNO**: Radius (µm) -, 5: 7.33 ± 1.31, 6: 9.77 ± 1.41, 7: 10.58 ± 1.33, 8: 10.45 ± 1.97; 9: 11.75 ± 2.87, 10: 12.90 ± 3.00, 11: 14.88 ± 3.31, 12: 14.89 ± 3.16, 13: 16.69 ± 3.48, 14: 17.52 ± 4.08, 15: 18.29 ± 4.18, 16: 18.10 ± 3.421, 17: 17.71 ± 2.50, 18: 17.96 ± 3.38, 19: 17.27 ± 3.92, 20: 18.44 ± 3.66, 21: 16.63 ± 3.53, 22: 16.94 ± 3.78, 23: 16.45 ± 4.00, 24: 15.60 ± 4.55, 25: 13.81 ± 4.24, 26: 12.19 ± 3.91, 27: 12.88 ± 3.71, 28: 13.15 ± 4.17, 29: 11.63 ± 4.03, 30: 9.69 ± 2.99, 31: 8.00 ± 3.15, 32: 7.88 ± 2.87, 33: 7.67 ± 2.78, 34: 6.13 ± 1.53, 35: 5.85 ± 2.43, 36: 6.40 ± 2.60, 37: 5.60 ± 2.31, 38: 4.71 ± 1.56, 39: 3.96 ± 1.75, 40: 3.92 ± 1.78, 41: 2,85 ± 1.51, 42: 1.83 ± 1.00, 43: 2.42 ± 1.31, 44: 1.75 ± 0.83, 45: 1.50 ± 0.84, 46: 1.25 ± 0.75, 47: 1.17 ± 0.73, 48: 0.83 ± 0.55, 49: 0.67 ± 0.40, 50: 0.50 ± 0.288, 51: 0.25 ± 0.25, 52: 0.08 ± 0.08, 53: 0 ± 0, 54: 0 ± 0, 55: 0 ± 0 **hm3Dq+CNO:** 5: 9.33 ± 1.58, 6: 10.77 ± 2.18, 7: 12.75 ± 2.12, 8: 15.58 ± 2.67; 9: 15.91 ± 2.24, 10: 15,17 ± 2.73, 11: 16.58 ± 2.75, 12: 16.00 ± 3.49, 13: 16.67 ± 2.51, 14: 16.33 ± 2.41, 15: 17.08 ± 3.48, 16: 17.83 ± 3.55, 17: 17.17 ± 1.98, 18: 14.75 ± 3.06, 19: 15.50 ± 3.06, 20: 11.75 ± 2.06, 21: 09.75 ± 1.93, 22: 11.41 ± 2.61, 23: 10.33 ± 3.00, 24: 09.00 ± 1.90, 25: 7.92 ± 1.61, 26: 07.92 ± 1.62, 27: 07.00 ± 1.57, 28: 7.25 ± 2.07, 29: 06.25 ± 2.20, 30: 4.75 ± 1.40, 31: 04.67 ± 1.82, 32: 3.41 ± 1.54, 33: 02.91 ± 1.31, 34: 02.25 ± 0.76, 35: 01.75 ± 0.75, 36: 1.42 ± 0.82, 37: 1.67 ± 1.00, 38: 1.42 ± 0.84, 39: 1.08 ± 0.63, 40: 1.25 ± 0.80, 41: 1.00 ± 0.59, 42: 0.75 ± 0.63, 43: 0.92 ± 0.63, 44: 0.58 ± 0.49, 45: 0.58 ± 0.44, 46: 0.50 ± 0.32, 47: 0.42 ± 0.32, 48: 0.33 ± 0.23, 49: 0.33 ± 0.19, 50: 0.25 ± 0.16, 51: 0.25 ± 0.16, 52: 0.67 ± 0.41, 53: 0.17 ± 0.17, 54: 0 ± 0, 55: 0 ± 0	[brains] n = 4 for both groups	2-way Anova	F_{group} (1,298): 18.64, p < 0.001 (***)
**FIGURE S8**	Measurement	Values	N	Statistical	P value
Figure S8E	Iba1+ cells (mean ± SEM)	**Iba1+**: hm3Dq-CNO: 202.10 ± 5.82; hm3Dq+CNO: 249.06 ± 10.04	[brains] hm3Dq-CNO: n = 3, hm3Dq+CNO: n = 4	2-tailed unpaired Student's t-test	p = 0.015 (*)
Figure S8F	Iba1+ cells (mean ± SEM)	**Iba1+** : hm3Dq-CNO: 241.54 ± 9.08; hm3Dq+CNO: 315.99 ± 25.39	[brains] n = 4 for all groups	2-tailed unpaired Student's t-test	p = 0.033 (*)
Figure S8G	Iba1+ cells (mean ± SEM)	**Iba1+**: hm3Dq-CNO: 247.76 ± 15.65; hm3Dq+CNO: 258.50 ± 11.31	[brains] n = 5 for all groups	2-tailed unpaired Student's t-test	p = 0.5932
Figure S8H	Iba1+ cells (% ± SEM)	**Iba1+**: hm3Dq-CNO: L1: 11.31 ± 0.47, L2/3: 28.86 ± 1.79; L4: 10.61 ±. 0.57, L5: 26.75 ± 1.22, L6: 22.47 ± 1.24; hm3Dq+CNO: L1: 11.34 ± 0.55, L2/3: 29.36 ± 0.47; L4: 11.88 ± 0.45, L5: 24.60 ± 0.58, L6: 22.83 ± 0.68	[brains] hm3Dq-CNO: n = 3, hm3Dq+CNO: n = 4	2-way Anova	NS
Figure S8I	Iba1+ cells (% ± SEM)	**Iba1+**: hm3Dq-CNO: L1: 10.64 ± 0.32, L2/3: 27.01 ± 0.84; L4: 12.63 ±. 0.86, L5: 26.20 ± 1.40, L6: 23.52 ± 0.53; hm3Dq+CNO: L1: 9.90 ± 0.57, L2/3: 26.79 ± 1.27; L4: 13.39 ± 0.36, L5: 26.57 ± 0.30, L6: 23.37 ± 0.84	[brains] n = 4 for all groups	2-way Anova	NS
Figure S8J	Iba1+ cells (% ± SEM)	**Iba1+**: hm3Dq-CNO: L1: 10.20 ± 0.78, L2/3: 27.48 ± 0.96; L4: 12.98 ±. 0.43, L5: 26.44 ± 1.08, L6: 22.90 ± 0.75; hm3Dq+CNO: L1: 11.65 ± 1.01, L2/3: 28.53 ± 0.96; L4: 11.67 ± 0.53, L5: 26.45 ± 0.87, L6: 21.70 ± 1.41	[brains] n = 5 for all groups	2-way Anova	NS
**FIGURE S9**	Measurement	Values	N	Statistical	P value
Figure S9A	Iba1+ cells (% ± SEM)	**Iba1+**: LD: L1: 15.89 ± 0.64, L2/3: 27.52 ± 0.36; L4: 12.40 ±. 0.36, L5: 27.65 ± 0.48, L6: 16.54 ± 0.44; DD: L1: 15.21 ± 0.47, L2/3: 26.01 ± 0.52; L4: 12.75 ± 0.33, L5: 27.44 ± 0.36, L6: 18.59 ± 0.46	[brains] n = 12 for all groups	2-way Anova	NS
Figures S9B	Iba1+ cells (% ± SEM)	Iba1+: LD: 224.35 ± 5.67: DD: 250.13 ± 9.53	[brains] n = 7 for all groups	2-tailed unpaired Student's t-test	p = 0.038 (*)
Figure S9C	Iba1+ cells (% ± SEM)	**Iba1+**: LD: L1: 13.72 ± 0.64, L2/3: 25.41 ± 1.31; L4: 13.86 ±. 0.39, L5: 26.38 ± 1.07, L6: 20.62 ± 0.99; DD: L1: 13.60 ± 0.77, L2/3: 25.46 ± 0.74; L4: 15.50 ± 0.50, L5: 25.54 ± 0.51, L6: 19.89 ± 0.68	[brains] n = 7 for all groups	2-way Anova	NS
**FIGURE S11**	Measurement	Values	N	Statistical	P value
Figure S11C	Cumulative distribution Activin+ immunofluorescence intensity	**Activin+ (fraction):** -CNO: Bin 10000: 0.000, Bin 20000: 0.044, Bin 30000: 0.250, Bin 40000: 0.676, Bin 50000: 0.809, Bin 60000: 0.926, Bin 70000: 0.941, Bin 80000: 0.985, Bin 90000: 1.00; +CNO: Bin 00000: 0.014, Bin 10000: 0.300, Bin 20000: 0.500, Bin 30000: 0.786, Bin 40000: 0.871, Bin 50000: 0.928, Bin 60000: 0.971, Bin 70000: 0.985, Bin 80000: 0.986, Bin 90000: 1.00	[cells]LD: n = 68 from 4 mice, DD: n = 70 from 4 mice	Kolmogrov-Smirnov test	p < 0.001 (***)
**FIGURE S12**	Measurement	Values	N	Statistical	P value
Figure S12D	Iba1+ cells (mean ± SEM)	**Iba1+**: LD + shLacZ: 566.78 ± 20.99; DD + shLacZ: 713.70 ± 28.23; DD + shInhba: 573.71 ± 23.15	[brains] n = 4 for LD + shLacZ ; n = 5 for DD + shInhba and DD + shLacZ groups	1-way Anova	p = 0.0049 (**) (LD+ shLacZ vs DD + shLacZ); p = 0.0045 (DD + shLacZ vs DD +shInhba)
Figure S12E	Iba1+ cells volume (mean ± SEM)	**Iba1+ cell volume**: LD + shLacZ: 2.62 x 10_5± 0.18; DD + shLacZ: 1.65 x 105± 0.09; DD + shInhba: 2.40 x 105± 0.20	[cells]LD + shLacZ: n = 14 from 5 mice, DD + shLacZ: n = 20 from 4 mice, DD + shInhba: b =18 from 4 mice	1-way Anova	p = 0.0003 (***); p = 0.0029 (**)

Figure S12F	Iba1+ cells (% ± SEM)	**Iba1+**:LD + shLacZ:: L1: 16.77 ± 0.29, L2/3: 24.03 ± 1.69; L4: 13.64 ±. 0.90, L5: 25.88 ± 1.35, L6: 19.69 ± 0.84; DD+shLacZ : L1: 17.19 ± 1.10, L2/3: 24.09 ± 1.35; L4: 11.18 ± 0.29, L5: 25.29 ± 0.73, L6: 22.26 ± 1.27; DD+shInhba : L1: 16.39 ± 1.43, L2/3: 24.03 ± 1.69; L4: 13.64 ± 0.90, L5: 26.33 ± 0.94, L6: 19.73 ± 0.92	[cells]LD + shLacZ: n = 14 from 5 mice, DD + shLacZ: n = 20 from 4 mice, DD + shInhba: b =18 from 4 mice	2-way Anova	NS
Figure S12G	Cumulative distribution P2RY12+ immunofluorescence intensity	**P2RY12+ Iba1+ (fraction):** LD + shLacZ: Bin 0: 0, Bin 20: 0.35, Bin 40: 0.70, Bin 60: 0.90, Bin 80: 0.98, Bin 100: 0.99, Bin 120: 1, Bin 140: 1, Bin 160: 1, Bin 180: 1, Bin 200: 1 ; DD+shLacZ : Bin 0: 0.004, Bin 20: 0.53: Bin 40: 0.75, Bin 60: 0.90, Bin 80: 0.96, Bin 100: 0.98, Bin 120: 0.99, Bin 140: 1, Bin 160: 1, Bin 180: 1, Bin 200: 1; DD + shInhba: Bin 0: 0.03, Bin 20: 0.27, Bin 40: 0.59, Bin 60: 0.79, Bin 80: 0.88, Bin 100: 0.93, Bin 120: 0.95, Bin 140: 0.97, Bin 160: 0.98, Bin 180: 0.99, Bin 200: 1	[Cells] LD + shLacZ: n = 243 from 4 mice, DD + shLacZ: n = 249 from 5 mice, DD + shInhba: n = 260 from 5 mice	Kruskal Wallis test	p = 0.005 (**) ; p = 0.047 (*); p < 0.0001 (***)
Figure S12H	Iba1+ scholl analysis (mean ± SEM)	**Interactions** : LD + shLacZ: Bin 5: 7.9 ± 0.35, 10: 15.38 ± 0.30, 15: 16.4 ± 0.49, 20: 17.78 ± 0.97, 25: 12.73 ± 1.56, 30: 8.24 ± 2.44, 35: 3.96 ± 1.07, 40: 1.93 ± 0.68, 45: 0.48 ± 0.08, 50: 0.11 ± 0.11, 55: 0 ± 0; DD + shLacZ: Bin 5: 5.46 ± 1.31, 10: 9.43 ± 0.99, 15: 13.80 ± 2.86, 20: 10.09 ± 0.60, 25: 7.22 ± 1.55, 30: 3.66 ± 0.98, 35: 2.05 ± 0.71, 40: 0.723 ± 0.44, 45: 0.25 ± 0.25, 50: 0 ± 0, 55: 0 ± 0; DD + shInhba: Bin 5: 7.69 ± 1.17, 10: 16.56 ± 1.92, 15: 19.44 ± 0.54, 20: 14.38 ± 1.75, 25: 10.13 ± 1.38, 30: 4.81 ± 0.41, 35: 1.94 ± 0.61, 40: 0.69 ± 0.31, 45: 0.38 ± 0.24, 50: 0.063 ± 0.063, 55: 0 ± 0,	[brains] n = 4 for LD + shLacZ ; n = 5 for DD + shInhba and DD + shLacZ groups	2-way Anova	F_{group} (1,6): 20.93, p < 0.0001 (***)
**FIGURE S13**	Measurement	Values	N	Statistical	P value
Figure S13C	Acvr1b immunofluorescence intensity	Intensity: Control: 1 ± 0.12; Mutant: 0.27 ± 0.07	[Cells] Control: n = 19 from 4 mice, Mutant: n = 18 from 4 mice	Mann Whitney	p < 0.001 (***)
**FIGURE S14**	Measurement	Values	N	Statistical	P value
Figure S14A	Iba1+ cells (% ± SEM)	**Iba1+**: Control: L1: 14.05 ± 0.70, L2/3: 25.94 ± 1.25; L4: 14.1 ±. 0.49, L5: 26.4 ± 0.92, L6: 19.5 ± 0.88; Mutant: L1: 14.22 ± 0.85, L2/3: 26.77 ± 0.89; L4: 13.72 ± 1.18, L5: 27.02 ± 0.79, L6: 18.28 ± 0.77	[Mice] Control: n = 7, Mutant: n = 4	2-way Anova	NS
**FIGURE S15**	Measurement	Values	N	Statistical	P value
Figure S15B	Iba1+ cells (mean ± SEM)	**Iba1+**: Control: 451.43 ± 8.26; Mutant: 327.77 ± 15.60	[brains] Control: n = 7, Mutant: n = 4	2-tailed unpaired Student's t-test	p < 0.001 (***)
Figure S15C	Iba1+ cells (% ± SEM)	**Iba1+**: Control: L1: 10.15 ± 1.10, L2/3: 28.20 ± 0.56; L4: 14.50 ±. 0.26, L5: 27.04 ± 0.60, L6: 20.13 ± 0.75; Mutant: L1: 10.41 ± 1.10, L2/3: 30.13 ± 2.17; L4: 14.35 ± 0.55, L5: 25.45 ± 0.96, L6: 19.66 ± 1.66	[Mice] Control: n = 7, Mutant: n = 4	2-way Anova	NS
**FIGURE S16**	Measurement	Values	N	Statistical	P value
Figure S16C	Iba1+ cells (% ± SEM)	**Iba1+**: 40.27 ± 1.48	[brains] Control: n = 2, Mutant: n = 3		
Figure S16D	Iba1+ cells (% ± SEM)	**Iba1+**: Control: L1: 10.90 ± 1.17, L2/3: 30.15 ± 0.21; L4: 15.27 ±. 2.74, L5: 25.89 ± 0.70, L6: 17.83 ± 2.48; Mutant: L1: 10.78 ± 1.23, L2/3: 32.33 ± 1.15; L4: 16.37 ± 0.84, L5: 28.22 ± 0.99, L6: 12.30 ± 0.60	[brains] Control: n = 2, Mutant: n = 3		
Figure S16F	Iba1+ cells (mean ± SEM)	**Iba1+**: Control - PLX: 318.89 ± 6.70, Control+ PLX: 316.46 ± 13.95;Mutant + PLX: 230.85 ±. 6.90	[brains] Control – PLX: n = 3, Control + PLX: n =4, Mutant + PLX: n = 3	1-way Anova	F =15.45, Control - PLX *vs* Mutant + PLX, *p* =0.005 (**), Control + PLX *vs* Mutant + PLX, *p* = 0.004 (**)
Figure S16G	Iba1+ cells (% ± SEM)	**Iba1+**: Control - PLX: L1: 10.89 ± 1.59, L2/3: 27.99 ± 1.65; L4: 14.40 ±. 1.19, L5: 25.82 ± 0.47, L6: 20.93 ± 1.52; Control+ PLX: L1: 9.96 ± 1.11, L2/3: 29.00 ± 0.97; L4: 14.46 ± 0.14, L5: 25.84 ± 0.66, L6: 20.74 ± 0.75; Mutant+ PLX: L1: 10.50 ± 0.47, L2/3: 26.79 ± 1.15; L4: 14.37 ± 0.76, L5: 26.86 ± 1.15, L6: 21.48 ± 1.26	[brains] Control – PLX: n = 3, Control + PLX: n =4, Mutant + PLX: n = 3	2-way Anova	$F(8,35)_{interaction}$ = 0.46
**FIGURE S17**	Measurement	Values	N	Statistical	P value
Figure S17B	Average speed (mean ± SEM)	**Speed**: Control: 0.072 ± 0.004; Mutant: 0.083 ± 0.001	[Mice] Control: n = 8, Mutant: n = 6	Mann Whitney	*p* = 0.047 (*)
Figure S17C	Time spent in zone (mean ± SEM)	**Time**: Control-Outer: 407.56 ± 18.72;Control-Inner: 191.96 ± 18.76; Mutant - Outer: 402.73 ± 14.32; Mutant - Inner: 196.95 ± 14.29	[Mice] Control: n = 8, Mutant: n = 6	1-way Anova	F = 44.95, *p* < 0.0001 (***)
Figure S17E	Time spent in zone (mean ± SEM)	**Time**: Control-Light: 267.95 ± 28.51;Control-Dark: 332.05 ± 28.51; Mutant - Light: 227.88 ± 28.51; Mutant -Dark: 322.12 ± 22.79	[Mice] Control: n = 8, Mutant: n = 6	1-way Anova	F = 1.46, *p* = NS
Figure S17B	Entries into the dark zone (mean ± SEM)	**Entries:** Control: 22.5 ± 2.41; Mutant: 26 ± 2.41	[Mice] Control: n = 8, Mutant: n = 6	2-tailed unpaired Student's t-test	*p* = 0.3352
**FIGURE S18**	Measurement	Values	N	Statistical	P value

Figure S18A	Rearing incidences (mean ± SEM)	**Rearing incidences**: Control - male: 95.5± 2.25; Control - female: 127.67 ± 3.79;Mutant - female: 83.25 ± 6.37; Mutant - female: 91.34 ± 4.11	[Mice] Control: n = 8, Mutant: n = 6	1-way Anova	F = 10.706, *p* = 0.0124 (*), *p* = 00014 (**, mutant male vs control female) *p* = 0.0088 (*, mutant male vs mutant female)

Table S2. List of receptor-ligand pairs

Receptors	Ligands	Activity-dependent ligand in adult visual cortex[45]	Pyramidal cell expression at P12 FPKM[43]
TYROBP	SEMA6D		
P2RY12	GNAI2		
CSF1R	CSF3, CSF1, CSF2, IL34		
CX3CR1	CX3CL1		
CD81	C3, GPC3, CD99		
CD68	S100A9, S100A8		
SIRPA	VEGFA, VWF, CSF1, SFTPD		
ITGAM	SPON2, KNG1, FGB, FGA, PLG, LPA, ICAM4, HP, CFH, CCN2, CD40LG, MMP9, PLAU, F10, CCN1, ICAM2, PLAT, SELPLG, APOB, PROC,C3, ICAM1		
CD53	IL2, IL4		
LAMP1	FAM3C, AMELX		
CCR5	GNAI2, S100A4, CCL5, CCL14, CCL11, CCL3, CCL7, CCL16, CCL3L3, CCL2, CCL5, ORM1,IL16,CCL4, CCL8		
TGFBR1	TGFB1, GNB3,TGFB3, CGN, TGBFB2, GDF9		
IFNGR1	IFNG		
F11R	CGN, ASIP, AFDN		
CD33	LGALS3BP		
TLR7	HSP90B1, IRAK4		
ITGB5	TGFB3,VTN, PLAU, EDIL3, SORBS1, SPP1,TLN1, COL4A2,CCN1, ADAM9, COL18A1, ITGB3BP, LTBP3, LTBP1, VCL,TGFB1, SERPINE1		
MERTK	GAS6, PROS1		
CANX	HLA-B		
HAVCR2	LGALS9		
CD9	IZUMO1, HBEGF, ADAM2		
IFNGR2	IFNG		
IL10RA	IL22, IL10		
GPR84	ADCYAP1, CGA, SCT, LHB, IAPP, TSHB, PTH2, CRH, PTH, CALCA, GIP, VIP, GHRH, INSL3, AVP, RLN2, ADM2, NPS, POMC, GCG, RLN3, FSHB, ADM, PTHLH, CALCB,	CRH	0 04
ITGB1	LAMA4, TGFB3, LAMA5, LAMC1M THBS1, SEMA7A, TIMP2, LGALS3BP, LAMC2, NID1, F13A1, VCAN, TNC, ADAM2, LUM, VCAM1, COL6A3, CSF2, COL1A2, VEGFD, JAM3, COL6A2, CD14, COL3A1, FGB, FGA, PLG, ADAM17, LAMA3, FBN1, FBLN1, COL4A5, COL7A1, FGG, COL9A3, ICAM4, FN1, COL18A1, ADAM15, COL9A1, LAMC3, COL6A1, TGM2, THBS2, ADAM12, COL11A1, COL5A1, COL9A2, PLAU, COL16A1, HSPG2, COL4A1, COL5A2, AGRN, COL2A1, LAMA1, LAMB3, COL4A6, SPP1, CXCL12, VEGFC, LAMA2, LAMB2, NPNT, DSPP, MDK, COL4A4, RELN, COL4A3, ADAM9, CHAD, ANGPT1, VEGFA, LGALS1, TGFB1, COMP, HGF, LAMB1, COL1A1, VTN		
ADRB2	LHB, APOB, IAPP, TSHB,IL1B, WNT5A, EGF,PTH2, CRH, PTH, ARPC5, GHRH, AVP, PTHLH, TF, POMC, RLN2, HSPA8, ADCYAP1, CALCA, ADM, CGA,GCG,VIP, ADM2, NPS, FSHB, CALCB, ACTR2, RLN3, GIP, INSL3	HSPA8	0
PLXNB2	SEMA4D, SEMA4G, SEMA4C, SEMA4A		
CSF3R	CSF2, CSF3		
ITGA6	ADAM2, FN1, LAMC3, COL6A1, THBS2, LAMA1, LAMB3, MDK, LAMA2, ADAM9, LAMB1, LAMA4, LAMA5, LAMC1, THBS1, LAMC2		
TGFBR2	TGFB2, CGN, TGFB1, TGFB3		
C3AR1	C3, C4A		
TNFRSF21	APP, TNF		
NPTN	PIP		
ATP6AP2	REN, WNT3A,		
P2RX7	CAMP		
TSPAN14	ADAM10		
ITGB2	ICAM1, C3, KNG1, VCAM1, JAM3, CD14, FGB, FGA, PLG, LPA, FGG, ICAM4, HP, CCN2, S100A8, S100A9,CD40LG,MMP9, PLAU, F10, CCN1, SPON2, ICAM2, ICAM3, PLAT, ICAM4, SELPLG, APOB, PROC,		
SLC40A1	CP		
TLR1	BGN, CD14, VCAN, HSP90B1		
ADIPOR1	ADIPOQ		
CMKLR1	RARRES2		
LRP1	F9, PLAT, SERPINE1, APOC3, LTF, TFPI, APOB, APOA1, APP, APOA2, AGRN, GPC3, PDGFB, A2M,APOC2,SERPINA1, SERPINE2, HSPG2, MMP13, APOE,PCSK9, MMP9, HSP90B1, MDK, PLAU, LRPAP1, C3, FCN3, THBS1, HSP90AA1, APOA4, CALR,C1QB, C4BPA, VWF, F8, CCN2, HPX, APP, LPL, LIPC, PF4, PSAP, WNT3A		
HFE	TF, B2M, RGMA		
CSF2RB	IL5, CSF2, IL3		
BMPR2	BMP1, RGMB, BMP8A, C4BPA, BMP5, BMP8B, GDF5, GDF9, BMP7, GDF2, RGMA, BMP4, BMP15, FST, GDF11, GDF7, BMP3, BMP6, GDF6, BMP10		
TLR9	CD14, HSP90B1, HMGB1, MMP9, HRAS		
TMEM219	IL13		
ACVR1	INHBC, BMP7, INHBB, GDF2, BMP2, BMP6, GDF5, TGFB2, AMH, INHBA, INHA	INHBA	11 54
CD63	TIMP1		
TLR6	IRAK4, APOB, CD14		
ITGA9	TNC, VEGFC, ADAM2, PLG, SPP1, F13A1, VEGFD, ADAM12, VCAM1, TGM2, VEGFA, CSF2, ADAM15, FN1		
IL13RA1	IL4, IL13		
AMFR	GPI		
IL21R	IL21, IL22		
CD74	MIF, APP		

CD48	IL18		
SORT1	NTS, GRN, PCSK9, NGF, LRPAP1, BDNF, PSAP	BDNF	17 01
CD47	VTN, COL4A6, LGALS9, COL4A4, COL4A3, THBS2, COL4A1, THBS1, COL4A5,		
LRP6	SOSTDC1, IGFBP4, RSPO1, PTH, WNT1, RSPO3, WNT2, WNT9B, CCN2, SOST, APP, CKLF, WNT5A, DKK1, WNT7A, DKK2, WNT3A, DKK4, WNT3, APOB, BMP4M APOE		
ABCA1	PLTP, APOA1, APOE, RIMS2, MEGF10, LIN7C, SHANK1, FADD		
LRP10	APP		
PTPRA	SPTBN2, NCAM1, CALM1, SPTAN1		
CD151	LAMB3, MMP7, LAMC2		
TLR2	HMGB1, CCN1, HSP90B1, BGN, SAA1, VCAN, ZG16B, HRAS, SFTPA1, RNASE2, APOC3		
TLR4	SFTPD, CCN1, HSPA1A, IRAK4, ZG16B, APOB, S100A1, HSP90B1, HSPA4, DEFB4A, CD14, FGB, FGA, BGN, FGG, HMGB1, HP, S100A8, S100A12, S100A9		
PTPRC	LGALS1		
SMO	WNT4, BMP2, SHH		
GP9	F2		
NCSTN	APP, PSEN1		
LTBR	TNFSF14, LTA, LTB		
ITGAV	L1CAM, TNC, PLAU, EDIL3, COL4A6, COL4A5, SPP1, NID, PDGFB, ICAM4, COL4A4, CALR, CCN1, FGG, FGA, COL4A3, MFGE8, FGB, FBN1, AZGP1, ADAM9, COL1A2, VEGFA, ADAM15, ANGPTL3, COL4A1, FN1, TGFB1, LAMB1, SERPINE1, COL1A1, VTN, IBSP, LAMA4, LAMC1		
TMED5	WNT7B		
TNFRSF11A	TNFSF11		
IL10RB	UCN2, IFNL2, IL22, IFNL1, IL10, IL24, UCN3, IFNL3, IL26		
IFNAR1	IFNA7, IFNW1, IFNA16, IFNA14, IFNA17, IFNA4, IFNA13, IFNA5, IFNA1, IFNB1, IFNA6, IFNA10, IFNA8, IFNE, MMP, IFNA21, IFNA2, IFNA7		
ITGB3	CCN1, VEGFA, TGFB1, COMP, VTN, IBSP, TGFB3, THBS1, VWF, NID1, TNC, MFGE8, COL1A2, FGB, FGA, TLN1, FBN1, HSP90AA1, FGG, DMP1, ICAM4, FN1, COL18A1, ADAM15, COL4A2, TGM2, THBS2, ANGPTL3, SPP1, COL4A3, COL4A4, FBLN2		
SCARB1	SAA1, APOE, THBS1		
KIDINS220	NGF		
RAMP1	IAPP, CALCA, ADM, VIP, ADM2, CALCB		
BMPR1A	GDF9, BMP7, BMP8B, BMP2, GDF5, BMP4, BMP15, GDF11, GDF7, BMP3, BMP6, GDF6, BMP10, BMP1, BMP8A, BMP5		
NOTCH2	DLK1, DLL1, MFNG, PSEN1, ADAM10, DLL3, DLL4, JAG1,		
C5AR1	C5, FN1, GNAI2		
CD82	HBEGF		
KCND1	IL16		
ENG	TGFB2, BMP7, GDF2, BMP2, TGFB1, INHBA, BMP10	INHBA	11 54
CCR1	CCL13, CCL5, CCL14, CCL3, CCL15, CCL23, CCL7, CCL16, CCL4, CCL26, CCL, CCL18, CCL2		
TNFRSF17	TNFSF13, TNFSF13B, CD70		
MRC2	PLAU, LGALS9, CER1		
ADCY7	GNAS, GNAI2		
PLXNA4	SEMA3A, SEMA6A, FARP2		
HRH2	HDC		
CADM1	ADAM10		
LIFR	CTF1, CLCF1, CNTF, OSM, LIF		
TLR5	ZG16B		
APLP2	APP, HLA-A, PCSK9		
ADIPOR2	ADIPOQ		
LRP5	DKK4, APOE, THBS1, CDH1, WNT5A, WNT3A, WNT1, WNT8A, SOST, WNT7B, WNT8B, DKK1		
PECAM1	MMP2, EFNB2		
IL7R	IL7, TSLP		
INSR	CALM2, SORBS1, INS, AHSG, ARF1, GIP, IFG2, HRAS, CALM1, CALM3, IGF1		
TNFRSF14	TNFSF14, LTA, CD160, BTLA, TNFSF13,		
TNFRSF13B	CD70, TNFSF13B, TNFSF13		
GPR35	CXCL17		
PTPRM	CDH1		
KLRD1	HLA-E, HLA-G, B2M, HLA-B		
LMBR1L	SCGB1A1, LCN1		
S1PR1	GNAI2, PDGFB		
IGF1R	CDH1, GPC3, INS, IGF2, GNAI2, IGF1, CAMP		
CD80	CD274		
CALCRL	ADM, CALCB, CALCA, ADM2		
ACVR2A	INHBC, GDF9, BMP7, CFC1, NODAL, BMP15, BMP10, GDF7, LEFTY1, INHA, INHBA, GDF1, INHBB, INHBE, LEFTY2, GDF2, TDGF1, BMP2, BMP6, GDF11, GDF5	INHBA	11 54
ACVRL1	GDF2, TGFB1, TGFB3, BMP10		
LRRC4	NTNG2		
TSPAN5	ADAM1O		
ACVR1B	LEFTY1, CFC1, GDF11, GDF1, INHBA, GDF10, LEFTY2, INHBC, TDGF1, INHBB, NODAL	INHBA	11 54
FZD7	WNT3A, WNT2, WNT11, WNT1, WNT3, WNT5A,		
KREMEN1	DKK1, DKK3		
NOTCH4	HLA-C, DLL3, DLL4, JAG1, PSEN1, JAG2, DLL1, THBS2,		
ITGA5	COL1A1, VTN, TNC, L1CAM, PLAU, SPP1, CCN1, FGG, FGA, VEGFD, CCN2, FGB, FBN1, ANGPT1, COL18A1, ADAM15, ADAM17, ANGPTL3, FN1		
CD200R1	CD200		
KCNJ10	IL16		
